# A facile and practical method for the synthesis of *trans*-(±)-taxifolin and its derivatives via Darzens reaction

**DOI:** 10.3762/bjoc.22.31

**Published:** 2026-03-12

**Authors:** Bo Peng, Panpan Yang, Maaz Khan, Xiaotong Lin, Jiang Wu, Peng Fu, Qingqing Wu

**Affiliations:** 1 School of Pharmaceutical Sciences, Anhui Medical University, Hefei 230032, China; 2 Zhongshan Institute for Drug Discovery, Shanghai Institute of Materia Medica, Chinese Academy of Sciences, Zhongshan 528400, China; 3 College of Pharmacy, Shenzhen Technology University, Shenzhen 518118, China

**Keywords:** Darzens reaction, derivatives, scale-up, synthesis, taxifolin

## Abstract

The synthesis of racemic *trans*-taxifolin (*trans*-(±)-taxifolin) and its derivatives and subsequent chiral separation is the most prevalent chemical method to obtain enantiomerically pure taxifolin and its derivatives. The development of an economical and practical synthetic route to *trans*-(±)-taxifolin, a key precursor to the enantiomerically pure *trans*-taxifolin, is therefore of great importance and significance. In this work, we developed a new synthetic method for *trans*-(±)-taxifolin and its derivatives with 2,4,6-trihydroxyacetophenone as a starting material undergoing hydroxy protection, α-bromination, construction of α,β-epoxy carbonyl products via the Darzens reaction, acid-mediated deprotection, and cyclization to afford the target compounds. This method is highlighted by satisfactory overall yields (20–41%) and proceeds without the use of explosive peroxides (such as H_2_O_2_), which are commonly employed in methods reported earlier. The avoidance of explosive peroxides in the present method enables safe operation, easy scale-up, and also the synthesis of taxifolin derivatives with oxidant-sensitive groups, largely expanding the substituent scope compared with the previous method.

## Introduction

Taxifolin is a sub-member of the flavonoid family with outstanding bioactive performance [1]. Several in vitro and in vivo experiments have demonstrated that taxifolin has unexpected pharmacological properties [2–4] such as antioxidant, anti-inflammatory, anti-apoptosis and neuroprotective effects. Thus, taxifolin exhibits great potential in the treatment of a series of diseases [3–5] involving organ or tissue injury, metabolic diseases, vascular diseases, cancer, neurologic diseases and so on. Notably, taxifolin has been approved as a novel dietary additive by the U.S. Food and Drug Administration (FDA) and is widely used as a food ingredient in various markets. It is to be expected that taxifolin undergoes transformation from dietary food to multifunctional medications in the near future.

**Figure 1:**
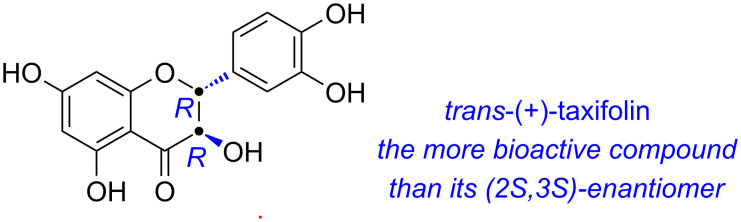
The structure of the highly bioactive *trans*-(+)-taxifolin.

Taxifolin is a chiral compound with two stereocenters and exists as *trans* or *cis* isomers. It is commonly referred to as the *trans* dextral form (*trans*-(+)-taxifolin), which has higher bioactivity than its levorotatory enantiomer [6] (Figure 1). Taxifolin can be obtained by extraction from natural plants [7–9], however, this is not economically viable due to the complicated extraction and purification process and low natural abundance of taxifolin. Enzymatic synthesis [10–14] is another method employed to produce taxifolin but also faces the problems of scale-up and high cost. As a result, the development of a practical synthetic approach is of importance and significance. A chiral synthesis for taxifolin was reported by the use of (+)-catechin as the starting material [15–16], but (+)-catechin is not readily available. Additionally, Xiang et al. [17] and Jew et al. [18] reported asymmetric syntheses of taxifolin, which suffer from long-step operation and the use of complicated and expensive metal-ligand catalysts. Currently, the most widely used chemical method is the synthesis of *trans*-(±)-taxifolin followed by chiral separation [16,19–30], which represents a relatively easily operated and controlled protocol. What is more important for this method is that it enables the synthesis of taxifolin derivatives, facilitating the exploration of their pharmacological properties.

**Scheme 1:**
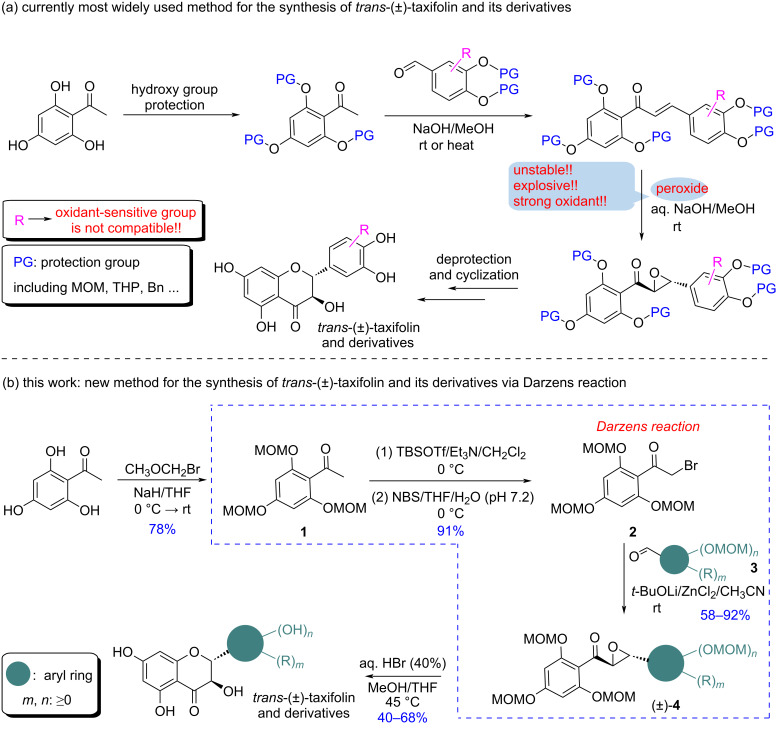
The currently most widely applied synthetic method for *trans*-(±)-taxifolin and its derivatives (a) and the new method via Darzens reaction reported in this work (b).

As the key precursor to enantiomerically pure *trans*-(+)-taxifolin and its derivatives, the development of an economical and practical synthetic route towards racemic *trans*-(±)-taxifolin or its derivatives has consistently attracted the attention of chemists. Currently, the most prevalent synthesis uses hydroxyacetophenones as starting material, whose hydroxy groups are first protected by various protecting groups and then reacted with aromatic aldehydes to form the corresponding chalcone intermediates. The chalcones are then oxidized with peroxides, such as H_2_O_2_, to give the α,β-epoxycarbonyl intermediates. The latter undergo simultaneous deprotection and cyclization by treatment with acid to afford the targeted *trans*-(±)-taxifolin and its derivatives [19–30] (Scheme 1a). Although this synthetic route is efficient, a significant drawback is that the construction of the α,β-epoxycarbonyl intermediates requires peroxides, which are not stable, highly oxidative, and potentially explosive. Therefore, this method suffers from risky operation and challenging scale up.

The Darzens reaction is a classical method to construct α,β-epoxycarbonyl compounds [31–32]. Inspired by this, in the present work, we designed a new synthetic method to *trans*-(±)-taxifolin and its derivatives based on the Darzens reaction (Scheme 1b). Starting from trihydroxyacetophenone, which was protected and α-brominated, the α,β-epoxycarbonyl intermediates were obtained via Darzens reaction. Subsequent acid-mediated deprotection and cyclization then afforded the target compounds. Compared with the widely used method involving peroxides reported earlier, the present method is highlighted with avoidance of the use of peroxides, therefore enabling safe scale-up and expanded synthesis of taxifolin derivatives with oxidant-sensitive substituents.

## Results and Discussion

2,4,6-Trihydroxyacetophenone was used as starting material, and its hydroxy groups were protected as methoxymethyl (MOM) ether by treatment with MOMBr (6.0 equiv) in the presence of NaH (4.0 equiv) to give protected acetophenone **1** in an excellent yield of 78%. Next, the reaction conditions for the following α-bromination of acetophenone **1** to intermediate **2** were screened (Table S1, Supporting Information File 1). The treatment of compound **1** with CuBr_2_ in EtOAc at either room temperature or 60 °C failed because of concomitant protecting group cleavage (Supporting Information File 1, Table S1, entries 1 and 2). Subsequently, a series of common brominating reagents, such as Br_2_, PTT (trimethylphenylammonium tribromide) and NBS were attempted, but unfortunately, cleavage of MOM was still observed (Supporting Information File 1, Table S1, entries 3–5, and 7). When NaHCO_3_ was used as an additive to neutralize HBr in the reaction mixture that was responsible for the cleavage of MOM, bromination with PTT was also unsuccessful in that the bromination occurred at the benzene ring and MOM was partially cleaved (Supporting Information File 1, Table S1, entry 6). To overcome these challenges, we decided to convert **1** to its corresponding silyl enol ether by treatment with TBSOTf in the presence of Et_3_N, and the formed silyl enol ether was in turn treated with NBS in a neutral THF/H_2_O mixture (PBS buffer) to give the desired brominated compound **2** (Supporting Information File 1, Table S1, entry 8). This two-step approach avoided acidic media, producing compound **2** smoothly with excellent yield (91%).

Next, we turned to the Darzens reaction between compound **2** and benzaldehyde **3** as the most crucial step in the synthetic route. Therefore, the reaction conditions of **2** and a selected benzaldehyde **3a** with two MOM-protected OH groups were optimized. Firstly, various organic and inorganic bases were screened (Table 1). The commonly used organic bases, such as Et_3_N, DBU, and pyridine, seemed not effective (Table 1, entries 1–3) in MeCN, giving a very low yield of the desired product (±)-**4a** and large amounts of polar by-products. In contrast, inorganic bases, such as K_2_CO_3_, Cs_2_CO_3_, NaOH, and KOH gave better results (Table 1, entries 4–7) in MeCN, especially using NaOH (62%) and KOH (60%). We then screened a series of metal alkoxides in MeCN (Table 1, entries 8–12), with *t*-BuOLi giving the best yield of 82% in a shortened reaction time (15 h). Furthermore, the solvent effect was studied (Table 1, entries 12–16), and MeCN was found to be the preferred solvent. It was reported that Lewis acids were favorable to catalyze the Darzens reaction [31,33], and some commonly used Lewis acids (Table 1, entries 17–22) were studied. The results showed that the addition of catalytic amounts of Lewis acids remarkably accelerated the reaction which was completed within 4 h while the yield was not affected. Among all the Lewis acids screened, ZnCl_2_ was associated with best yield of 90%.

**Table 1:** Reaction conditions optimization for the Darzens reaction of **2** with **3a**.^a^

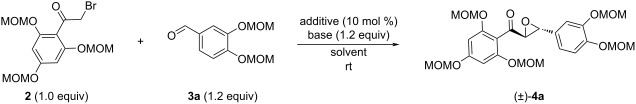					

Entries	Additive	Base	Solvent	Reaction time	Yield of **4a**^b^

1	–	Et_3_N	CH_3_CN	20 h	<10%
2	–	DBU	CH_3_CN	20 h	<10%
3	–	pyridine	CH_3_CN	20 h	20%
4	–	K_2_CO_3_	CH_3_CN	20 h	<10%
5	–	Cs_2_CO_3_	CH_3_CN	20 h	32%
6	–	NaOH	CH_3_CN	20 h	62%
7	–	KOH	CH_3_CN	20 h	60%
8	–	C_2_H_5_ONa	CH_3_CN	20 h	55%
9	–	CH_3_ONa	CH_3_CN	20 h	75%
10	–	*t*-BuOK	CH_3_CN	20 h	40%
11	–	*t*-BuONa	CH_3_CN	20 h	60%
12	–	*t*-BuOLi	CH_3_CN	15 h	82%
13	–	*t*-BuOLi	THF	15 h	73%
14	–	*t*-BuOLi	MeOH	15 h	79%
15	–	*t*-BuOLi	toluene	15 h	57%
16	–	*t*-BuOLi	DMF	15 h	74%
17	MgBr_2_	*t*-BuOLi	CH_3_CN	4 h	85%
18	MgCl_2_	*t*-BuOLi	CH_3_CN	4 h	76%
19	LiCl	*t*-BuOLi	CH_3_CN	4 h	79%
20	CuCl_2_	*t*-BuOLi	CH_3_CN	4 h	67%
21	Cu(OAc)_2_	*t*-BuOLi	CH_3_CN	4 h	83%
22	ZnCl_2_	*t*-BuOLi	CH_3_CN	4 h	90%

^a^Reaction conditions: **2** (0.81 mmol, 1.0 equiv), **3a** (1.2 equiv) and base (1.2 equiv) in solvent (4.5 mL) with or without addition of the additive (10 mol %), stirred at room temperature for the indicated time under N_2_ atmosphere; ^b^isolated yield.

Overall, the optimal conditions for the Darzens reaction involved treatment of **2** (1.0 equiv) with **3a** (1.2 equiv) in MeCN at room temperature, using *t*-BuOLi (1.2 equiv) as the base and ZnCl_2_ (0.1 equiv) as Lewis acid catalyst.

The stereochemical outcome of the Darzens reaction may produce the α,β-epoxycarbonyl products with either the substituents on the same side or opposite side of the epoxy functionality, i.e., as *cis*/*trans* isomers. In this work, the α,β-epoxycarbonyl product contains bulky multiple-substituted benzene moieties (such as bis- or tris-MOMO-substituted phenyl substituents). Due to the steric hindrance, the *trans*-configured product with the phenyl substituents on the opposite side of the epoxy functionality is obtained. Additionally, the clean reaction process shown by TLC and the NMR spectrum of the product further confirmed the formation of a single product.

**Scheme 2:**
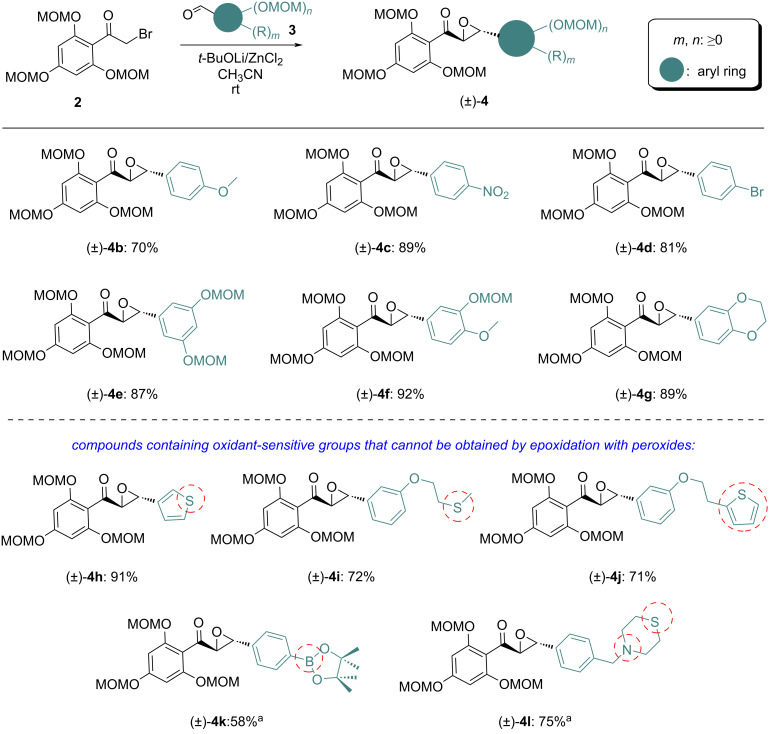
Darzens reaction of **2** with various arylaldehydes under the optimized conditions. Reaction conditions: **2** (0.81 mmol, 1.0 equiv), **3** (1.2 equiv), *t*-BuOLi (1.2 equiv) and ZnCl_2_ (10 mol %) in CH_3_CN (4.5 mL), stirred at room temperature for 4–6 h, N_2_ atmosphere. ^a^ZnCl_2_ was not added and the reaction time was 15 h. Yields are isolated yields.

To demonstrate the generality of the optimized reaction, compound **2** was reacted with a variety of aromatic aldehydes with different substituents (Scheme 2). The results showed that all reactions were efficient affording the α,β-epoxycarbonyl products (±)-**4** with yields in the range of 58–92% when the aromatic aldehyde was substituted with either electron-withdrawing (NO_2_) or electron-donating (alkoxy) groups. It is worth noting that aldehydes bearing oxidant-sensitive substituents, which are incompatible with peroxide-based approaches commonly employed for this type of transformation, were well tolerated under the present conditions. This observation highlights the broader substrate scope and enhanced practical applicability of the developed method.

Finally, deprotection of the products (±)-**4** followed by cyclization with acid yielded the target compounds. Although HCl is the commonly used acid for this step [19–30], we also screened some other acids and the results are collected in Table 2. As can be seen from the table, inorganic acids (Table 2, entries 1 and 2) generally gave better results than organic acids (Table 2, entries 3, 4, and 6), with 40% HBr giving the best yield (68%).

**Table 2:** Acid screening for the deprotection and cyclization of (±)-**4a** to *trans*-(±)-taxifolin.^a^

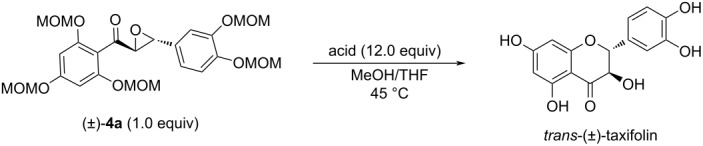			

Entry	Acid	Conversion of **1**	Yield of taxifolin^b^

1	36% HCl	100%	64%
2	40% HBr	100%	68%
3	*p*-TsOH·H_2_O	100%	56%
4	MsOH	100%	33%
5	98% H_2_SO_4_	100%	34%
6	CF_3_CO_2_H	100%	–^c^

^a^Reaction conditions: (±)-**4a** (0.95 mmol, 1.0 equiv) and acid (12.0 equiv) in MeOH (10 mL) and THF (1 mL), stirred at 45 °C for 1.5 h; ^b^isolated yield; ^c^a complex mixture instead of the desired product was obtained.

**Scheme 3:**
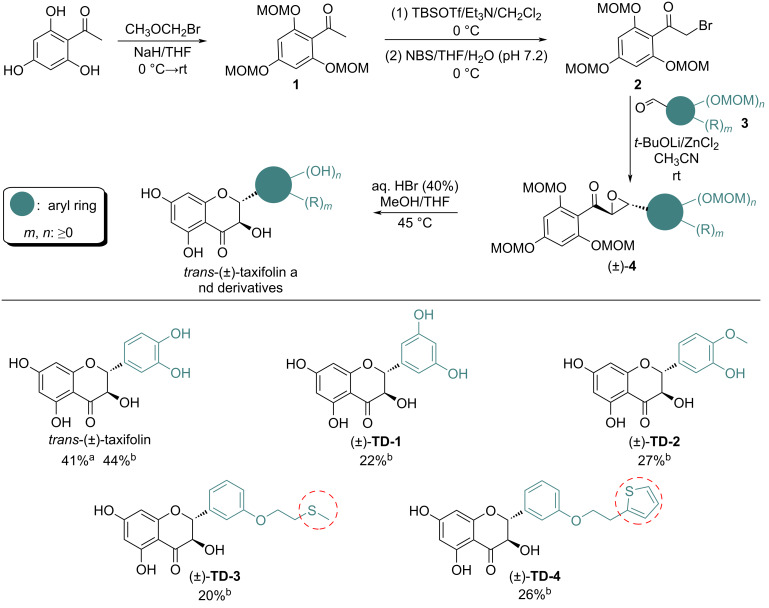
Synthesis of *trans*-(±)**-**taxifolin and its derivatives via the approach developed in this work. Yields are isolated yields; ^a^synthesis on 10 gram scale; ^b^synthesis on 1 gram scale.

After having optimized the conditions for each step, the synthesis of *trans*-(±)**-**taxifolin on a decagram scale (Scheme 3) was carried out to illustrate the practicality and scalability of the designed protocol. Ten grams of trihydroxyacetophenone were reacted with bromomethyl methyl ether (6.0 equiv) using NaH (4.0 equiv) as base in THF to give OH-protected acetophenone **1** with 78% yield. Compound **1** was then converted to the silyl enol ether via reaction with TBSOTf (1.2 equiv) in the presence of Et_3_N (3.0 equiv) in CH_2_Cl_2_ and no further purification was required after post-treatment. The formed silyl enol ether was then reacted with NBS (1.1 equiv) in a mixed solvent of THF and PBS buffer solution (pH 7.2) to give compound **2** with a total yield of 91% over the successive two steps. Darzens reaction of compound **2** (10.05 g) with benzaldehyde **3a** (1.2 equiv) was performed in CH_3_CN, using *t*-BuOLi (1.2 equiv) as base in the presence of a catalytic amount of ZnCl_2_. Due to the high efficiency of the reaction, no purification of product (±)-**4a** by column chromatography was necessary after post-treatment and the product could be directly used in the following step. Lastly, hydroxy deprotection and cyclization of (±)-**4a** using aqueous HBr solution (40%, 12.0 equiv) in MeOH/THF afforded 4.56 g of *trans*-(±)-taxifolin accounting for 41% yield starting from **2**. Overall, the scale-up reaction proceeded smoothly with comparable yield.

In addition to the synthesis of *trans*-(±)-taxifolin, some representative derivatives, such as (±)-**TD-1**, (±)-**TD-2**, (±)-**TD-3**, (±)-**TD-4** (Scheme 3), could also be synthesized readily by this method, highlighting the potential applicability of this method to structural optimization of the taxifolin skeleton. It is worth noting that the introduction of oxidant-sensitive groups, such as thiol, sulfide and conjugated double bonds into compounds is believed to be beneficial to the antioxidant activity. The present synthetic route developed in this work successfully also afforded such compounds as exemplified by the synthesis of (±)-**TD-3** and (±)-**TD-4** (Scheme 3). In contrast, the previously reported approach is not suitable to achieve this goal due to the use of peroxides.

## Conclusion

In conclusion, we developed a new facile and practical approach for the synthesis of *trans*-(±)-taxifolin and its derivatives with the Darzens reaction being the key step. It is highlighted by simple operation, high yields, and most importantly, by the avoidance of the use of peroxides (such as H_2_O_2_), which enables the safe scale-up and synthesis of taxifolin derivatives with oxidant-sensitive functionalities.

## Supporting Information

File 1 Reaction conditions screening, experimental section with characterization data and copies of spectra.

## Data Availability

All data that supports the findings of this study is available in the published article and/or the supporting information of this article.
