# Supplementary material for: A facile and practical method for the synthesis of trans-(±)-taxifolin and its derivatives via Darzens reaction
Source: Beilstein J Org Chem. 2026 Mar 12;22:443–50. doi: 10.3762/bjoc.22.31 (PMC12990444; doi:10.3762/bjoc.22.31)

BEILSTEIN JOURNAL OF ORGANIC CHEMISTRY

Supporting Information

for

A facile and practical method for the synthesis of *trans*-(±)-taxifolin and its derivatives via Darzens reaction

Bo Peng, Panpan Yang, Maaz Khan, Xiaotong Lin, Jiang Wu, Peng Fu and Qingqing Wu

Beilstein J. Org. Chem. **2026,** *22,* 443–450. doi:10.3762/bjoc.22.31

Reaction conditions screening, experimental section with characterization data and copies of spectra

License and Terms: This is a supporting information file under the terms of the Creative Commons Attribution License (https://creativecommons.org/licenses/by/4.0). Please note that the reuse, redistribution and reproduction in particular requires that the author(s) and source are credited and that individual graphics may be subject to special legal provisions.
The license is subject to the *Beilstein Journal of Organic Chemistry* terms and conditions: (https://www.beilstein-journals.org/bjoc/terms)

Table of contents

Table S1: Conditions screening S2

^{1}H NMR spectrum of isolated side products of **2** S3

Experimental section S4

References S15

Copies of NMR and HRMS spectra of the compounds S16

Table S1: Reaction condition screening for the α-bromation of acetophenone **1**

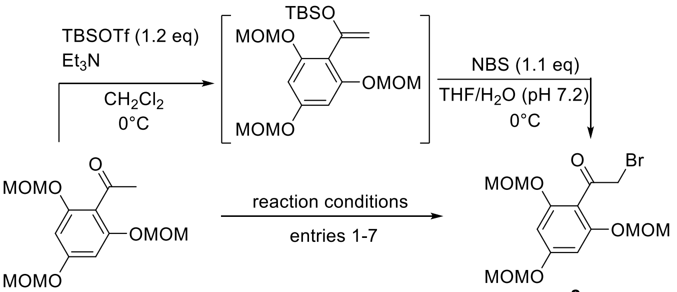

Entries	Brominating reagent	Additives	Solvent	Temperature	Conversion of **1**	Yield of **2**[d]
1	$CuBr_2$ (1.2 equiv)	/	EtOAc	60 °C	100% [b]	0%
2	$CuBr_2$ (1.2 equiv)	/	EtOAc	r.t.	100% [b]	0%
3	Br_2 (1.2 equiv)	/	CH_3OH	r.t.	100% [b]	0%
4	Br_2 (1.2 equiv)	/	AcOH	r.t.	100% [b]	0%
5	PTT [a] (1.2 equiv)	/	THF	r.t.	100% [b]	0%
6	PTT [a] (1.2 equiv)	$NaHCO_3$ (1.3 equiv)	THF	r.t.	100% [c]	0%
7	NBS (1.2 equiv)	*p*-TsOH (cat.)	THF	40 °C	100% [b]	0%
8[e]	NBS (1.1 equiv)	/	THF/H_2O	0 °C	100%	91%

[a] PTT: trimethylphenylammonium tribromide; [b] By-product with cleavage of MOM was obtained; [c] Bromination reaction occurred on the phenyl moiety and MOM was partially cleaved; [d] Isolated yield; [e] **1** was converted to silyl enol ether first.

^{1}H NMR spectrum of the isolated side product of **2** (Table S1, entries 2, 3, and 5).

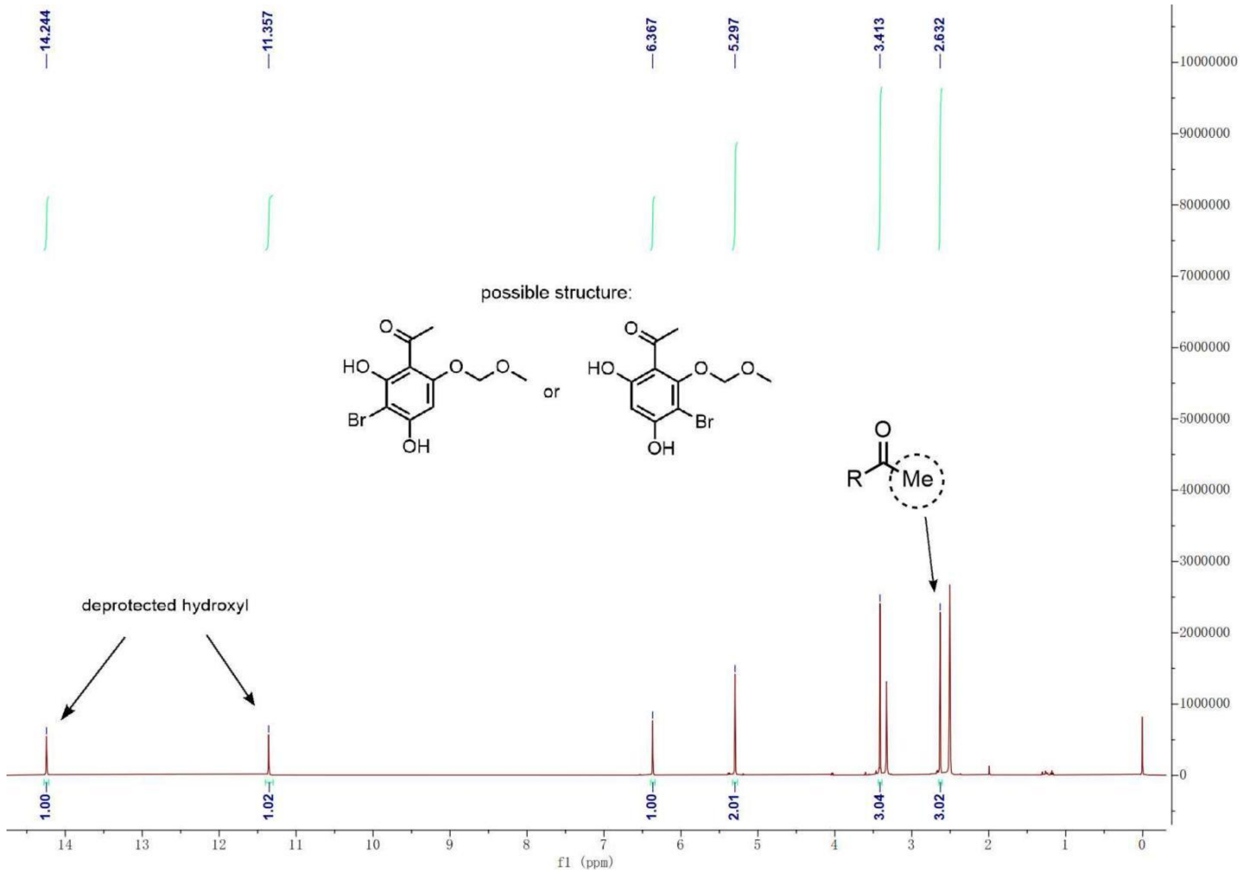

^{1}H NMR spectrum of the isolated side product of **2** (Table S1, entry 6)

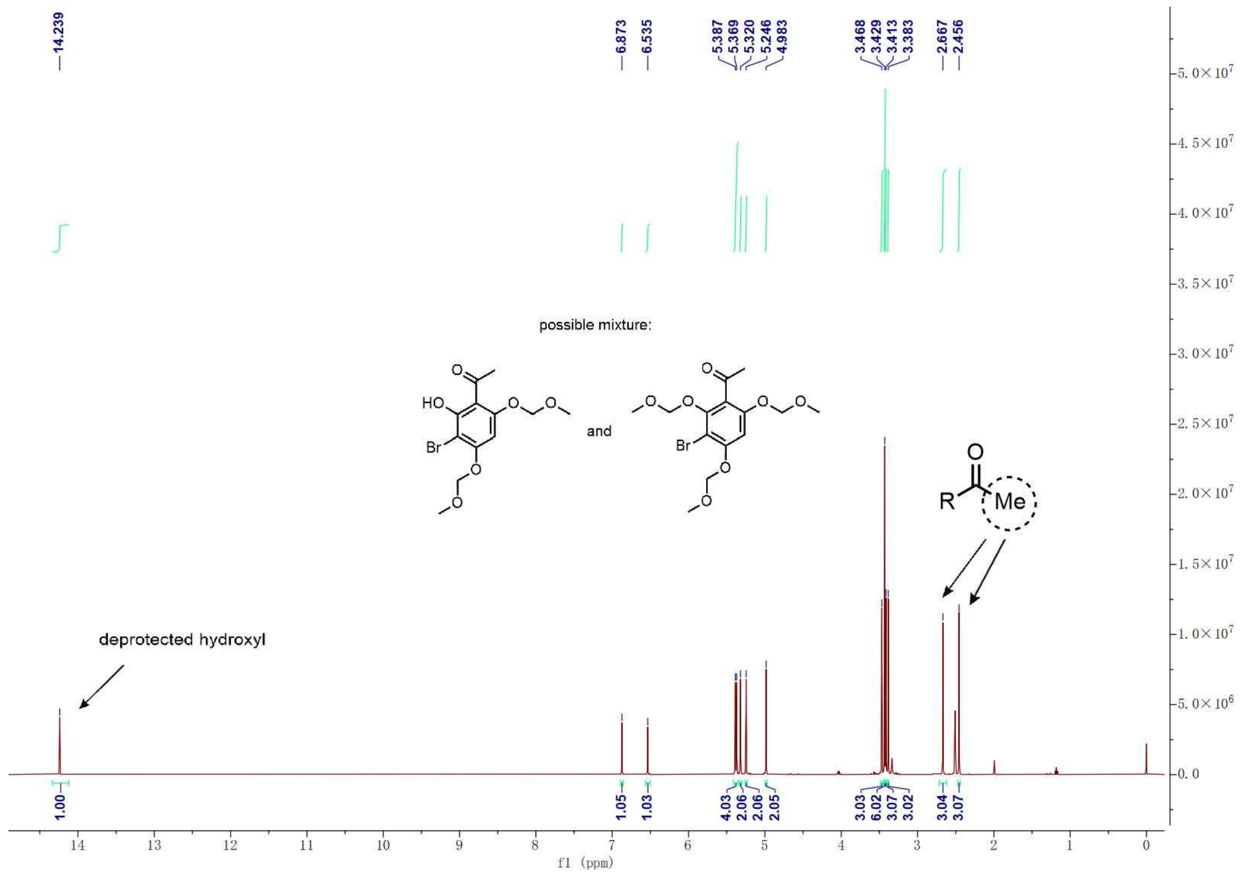

Experimental section

General

Unless stated otherwise, all the chemical reagents were obtained commercially and used without further purification. All the dried solvents were prepared by standard methods. Reaction progress was monitored by thin-layer chromatography (TLC) on commercially available pre-coated TLC silica gel plate, and separation and purification via flash column chromatography was performed on silica gel column (200–300 mesh) eluting with EtOAc/*n*-hexane (v/v). Melting points were measured in open capillaries with an SGW X-4A microscopic melting point apparatus (Shanghai INESA Physico-Optical Instrument Co., Ltd., Shanghai, China) and are uncorrected. NMR spectra were recorded on a Bruker Ascend 500 NMR spectrometer (Bruker Switzerland AG, Fällanden, Switzerland) using $CDCl_3$, DMSO-d_6 as solvent and TMS (for ^{1}H NMR) or known chemical shifts of carbon signals of deuterated solvents (for ^{13}C{^{1}H} NMR) as internal standard. High-resolution mass spectra (HRMS) were determined with a Thermo Scientific Exactive Plus mass spectrometer (Thermo Fisher Scientific, Bremen, Germany) using electrospray ionization (ESI) and Orbitrap techniques. Unless stated otherwise, the concentration or evaporation on a rotary evaporator was conducted at temperatures below 45 °C.

Compound synthesis

Synthesis of 1-(2,4,6-tris(methoxymethoxy)phenyl)ethan-1-one (1)

To a stirred solution of 2,4,6-trihydroxyacetophenone (10.02 g, 0.059 mol) in THF (150 mL) was added NaH (60%, 9.44 g, 0.24 mol) in portions at 0 ℃, and the reaction mixture was further stirred for 0.5 h, followed by addition of bomomethyl methyl ether (44.23 g, 0.35 mol) in a dropwise manner. After addition, the reaction mixture was stirred at room temperature for 12 h. Cold water (150 mL) was added slowly to quench the reaction and the resulting mixture was extracted with EtOAc (100 mL × 2). The combined organic phases were washed with saline (100 mL), dried with $MgSO_4$, and concentrated to afford a residue, which was purified via column chromatography (EtOAc/*n*-hexane 1:5, v/v) to give compound **1** (13.82 g, 78%) as a colorless oil. Rf = 0.32 (EtOAc/*n*-hexane = 1/5 v/v). ^{1}H NMR (500 MHz, $CDCl_3$) δ 6.43 (s, 2H), 5.06 (s, 2H), 5.06 (s, 4H), 3.39 (s, 3H), 3.38 (s, 6H), 2.41 (s, 3H). The ^{1}H NMR data were consistent with those reported [1].

Synthesis of 2-bromo-1-(2,4,6-tris(methoxymethoxy)phenyl)ethan-1-one (2)

To a stirred solution of **1** (13.70 g, 0.046 mol) and Et_3N (14.16 g, 0.14 mol) in CH_2Cl_2 (200 mL) was added TBSOTf (14.53 g, 0.055 mol) dropwise at 0 °C. After addition, the mixture thus obtained was stirred for 1.5 h. The reaction mixture was washed with cold saline (150 mL × 2), dried with $MgSO_4$ and concentrated in vacuo. The obtained residue was dissolved in the mixed solvent of THF (200 mL) and PBS buffer solution (30 mL, pH 7.2) at 0 °C, and *N*-bromosuccinimide (9.01 g, 0.051 mol) was added in portions. After addition, the reaction mixture was stirred for another 1.5 h, and then diluted with EtOAc (200 mL). The resulting mixture was washed successively with saline (120 mL) and saturated aqueous $NaHCO_3$ (100 mL), dried with $MgSO_4$, and concentrated to give a residue, which was purified via column chromatography (EtOAc/*n*-hexane 1:5, v/v) to give compound **2** (15.69 g, 91%) as a white solid. m.p. 42-44 °C. Rf = 0.34 (EtOAc/*n*-hexane = 1/5 v/v). ^1H NMR (500 MHz, DMSO-d_6) δ 6.50 (s, 2H), 5.20 (s, 2H), 5.19 (s, 4H), 4.56 (s, 2H), 3.39 (s, 3H), 3.36 (s, 6H).^{13}C NMR (126 MHz, DMSO-d_6) δ 192.8, 159.7, 155.3 (2C), 112.0, 96.4 (2C), 94.3 (2C), 93.9, 56.0 (2C), 55.8, 38.3. HR-MS, calcd for $C_{14}H_{19}BrO_7$ ($[M+H]^+$) 379.0387, found 379.0397.

Synthesis of 3,5-bis(methoxymethoxy)benzaldehyde (3e)

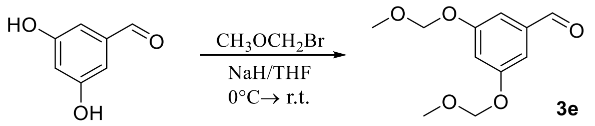

3e

To a stirred solution of 3,5-dihydroxybenzaldehyde (2.02 g, 0.015 mol) in THF (25 mL) at 0–5 °C was added NaH (60%, 3.02 g, 0.072 mol) in portions, and after addition, the resulting mixture was further stirred for 0.5 h, followed by addition of bromomethyl methyl ether (7.24 g, 0.058 mol) in a dropwise manner. After addition, the reaction mixture was stirred at room temperature for 12 h. Cold water (40 mL) was added slowly to quench the reaction, and mixture thus obtained was extracted with EtOAc (30 mL × 2). The combined extracts were washed with saline (50 mL), dried with $MgSO_4$ and concentrated in vacuo to afford a residue, which was purified via column chromatography (EtOAc/*n*-hexane 1:5, v/v) to give **3e** (1.78 g, 52%) as a colorless oil. Rf = 0.31 (EtOAc/*n*-hexane = 1/5 v/v). ^1H NMR (500 MHz, $CDCl_3$) δ 9.83 (s, 1H), 7.13 (d, J = 2.5 Hz, 2H), 6.90-6.89 (m, 1H), 5.13 (s, 4H), 3.41 (s, 6H). The ^1H NMR data are consistent with those reported in the known literature [2].

Synthesis of 4-methoxy-3-(methoxymethoxy)benzaldehyde (3f)

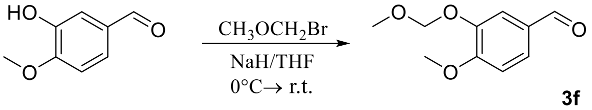

3f

To a stirred solution of 3-hydroxy-4-methoxybenzaldehyde (2.01 g, 0.013 mol) in THF (25 mL) at 0–5 °C was added NaH (60%, 1.42 g, 0.032 mol) in portions, and the reaction mixture was further stirred for 0.5 h, followed by addition of bromomethyl methyl ether (3.30 g, 0.026 mol) in a dropwise manner. After addition, the reaction mixture was stirred at room temperature for 12 h. Cold water (30 mL) was added slowly to quench the reaction, and the resulting mixture was extracted with EtOAc (30 mL × 2). The combined extracts were washed with saline (50 mL), dried with $MgSO_4$, and concentrated to afford a residue, which was purified via column chromatography (EtOAc/*n*-hexane 1:5, v/v) to give **3f** (2.34 g, 91%) as a colorless oil. Rf = 0.35 (EtOAc/*n*-hexane = 1/5 v/v). ^1H NMR (500 MHz, $CDCl_3$) δ 9.74 (s, 1H), 7.55 (d, J = 2.0 Hz, 2H), 7.45-7.43 (m, 1H), 6.92 (d, J = 8.5 Hz, 1H), 5.18 (s, 2H), 3.86 (s, 3H), 3.42 (s, 3H). The ^1H NMR data are consistent with those reported in the known literature [3].

Synthesis of 3-(2-(methylthio)ethoxy)benzaldehyde (3i)

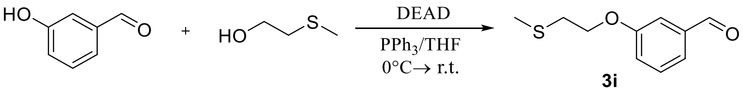

3i

To a stirred solution of 3-hydroxybenzaldehyde (5.01 g, 0.041 mol), 2-(methylthio) ethan-1-ol (4.15 g, 0.045 mol) and triphenylphosphine (11.80 g, 0.045 mol) in THF (100 mL) at 0–5 °C was added diethyl azodicarboxylate (7.85 g, 0.045 mmol) in portions. After addition, the reaction mixture was stirred at room temperature for 18 h. Water (100 mL) was added to quench the reaction, and the resulting mixture was extracted with EtOAc (150 mL × 2). The combined extracts were washed with saline (200 mL), dried with $MgSO_4$ and concentrated to afford a residue, which was purified via column chromatography (EtOAc/*n*-hexane 1:5, v/v) to give **3i** (3.11 g, 39%) as a colorless oil. Rf = 0.37 (EtOAc/*n*-hexane = 1/5 v/v). ^1H NMR (500 MHz, DMSO-d_6) δ 9.99 (s, 1H), 7.55-7.51 (m, 2H), 7.46-7.45 (m, 1H), 7.31-7.29 (m, 1H), 4.24 (t, J = 6.5 Hz, 2H), 2.17 (s, 3H).The ^1H NMR data are consistent with those reported in the known literature [4].

Synthesis of 3-(2-(thiophen-2-yl)ethoxy)benzaldehyde (3j)

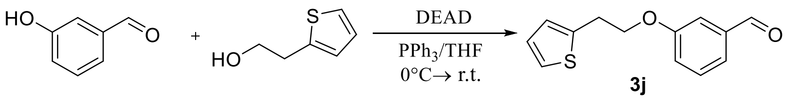

To a stirred solution of 3-hydroxybenzaldehyde (5.04 g, 0.041 mol), 2-(thiophen-2-yl)ethan-1-ol (5.75 g, 0.045 mol) and triphenylphosphine (11.80 g, 0.045 mol) in THF (100 mL) at 0–5 °C was added diethyl azodicarboxylate (7.85 g, 0.045 mol) in portions. After addition, the reaction mixture was stirred at room temperature for 18 h. Water (100 mL) was added to quench the reaction, and the resulting mixture was extracted with EtOAc (150 mL × 2). The combined extracts were washed with saline (200 mL), dried with $MgSO_4$ and concentrated to afford a residue, which was purified via column chromatography (EtOAc/*n*-hexane 1:5, v/v) to give **3j** (2.13 g, 22%) as a colorless oil. Rf = 0.40 (EtOAc/*n*-hexane = 1/5 v/v). ^{1}H NMR (500 MHz, $CDCl_3$) δ 9.96 (s, 1H), 7.47-7.40 (m, 3H), 7.21-7.17 (m, 2H), 6.97-6.93 (m, 2H), 4.25 (t, *J* = 6.5 Hz, 2H), 3.34 (t, *J* = 6.5 Hz, 2H). The ^{1}H NMR data are consistent with those reported in the known literature [5].

Synthesis of 4-(thiomorpholinomethyl)benzaldehyde (3l)

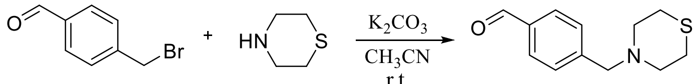

To a stirred solution of 4-(bromomethyl)benzaldehyde (1.00 g, 5.02 mmol) in CH_3CN (15 mL) was added thiomorpholine (1.05 g. 0.010 mol) and K_2CO_3 (1.60 g, 0.012 mol). The reaction mixture was stirred at room temperature for 24 h and then diluted with water (30 mL), the organic components were extracted with EtOAc (30 mL × 2), washed with saline (50 mL), dried with $MgSO_4$ and concentrated to afford a residue, which was purified via column chromatography (EtOAc/*n*-hexane 1:3, v/v) to give **3l** (0.99 g, 89%) as a white solid. m.p. 74-76 °C. Rf = 0.55 (EtOAc/*n*-hexane = 1/3 v/v). ^{1}H NMR (500 MHz, $CDCl_3$) δ 10.00 (s, 1H), 7.84 (d, *J* = 8.0 Hz, 2H), 7.50 (d, *J* = 8.0 Hz, 2H), 3.59 (s, 2H), 2.73-2.68 (m, 8H). The ^{1}H NMR data are consistent with those reported in the known literature [6]

Synthesis of α,β-epoxycarbonyl intermediate (±)-**4**

General procedure: To a stirred solution of **2** (0.30 g, 0.81 mmol) in CH_3CN (4.5 mL) was added arylaldehyde **3** (0.97 mmol), followed by successive additions of *t*-BuOLi (0.077 g, 0.96 mmol) and $ZnCl_2$ (11 mg, 0.081 mmol) at room temperature under N_2 atmosphere. The reaction mixture was stirred at room temperature for 4–6 h until TLC show that consumption of **2** was complete. The reaction mixture was diluted with EtOAc (10 mL), washed with saline (10 mL), dried with $MgSO_4$ and concentrated to afford a residue, which was purified via column chromatography (EtOAc/*n*-hexane 1:3, v/v) to give (±)-**4**.

((*2*S*,*3*R*)-3-(3,4-bis(methoxymethoxy)phenyl)oxiran-2-yl)(2,4,6-tris(methoxymethoxy)phen-yl)methanone ((±**)-4a)** yield: 90%, yellow oil. Rf = 0.31 (EtOAc/*n*-hexane = 1/3 v/v). ^{1}H NMR (500 MHz, DMSO-d_6) δ 7.09 (d, *J* = 8.5 Hz, 1H), 7.06 (d, *J* = 2.5 Hz, 1H), 6.99-6.97 (m, 1H), 6.49 (s, 2H), 5.23-5.11 (m, 10H), 4.07 (d, *J* = 2.0 Hz, 1H), 3.93 (d, *J* = 2.0 Hz, 1H), 3.38 (s, 9H), 3.28 (s, 6H). ^{13}C NMR (126 MHz, DMSO-d_6) δ 195.9, 159.9, 156.0 (2C), 147.3, 146.9, 129.5, 120.3, 116.9, 114.4, 112.1, 96.3 (2C), 94.7, 94.6, 94.2 (2C), 93.9, 63.2, 58.4, 55.9, 55.8 (2C), 55.6, 55.6. HR-MS, calcd for $C_{25}H_{32}O_{12}$ ([M+H]$^+$) 525.1967, found 525.1953.

((2*S,3*R**)-3-(4-Methoxyphenyl)oxiran-2-yl)(2,4,6-tris(methoxymethoxy)phenyl)methanone ((**±**)-4b)** yield: 70%, yellow solid. m.p. 55-57 °C. Rf = 0.34 (EtOAc/*n*-hexane = 1/3 v/v). ^{1}H NMR (500 MHz, DMSO-d_6) δ 7.29 (d, *J* = 8.5, 2H), 6.93 (d, *J* = 8.5 Hz, 2H), 6.49 (s, 2H), 5.20 (s, 2H), 5.16 (d, *J* = 7.0 Hz, 2H), 5.12 (d, *J* = 7.0 Hz, 2H), 4.10 (d, *J* = 2.0 Hz, 1H), 3.94 (d, *J* = 2.0 Hz, 1H), 3.75 (s, 3H), 3.38 (s, 3H), 3.29 (s, 6H). ^{13}C NMR (126 MHz, DMSO-d_6) δ 196.2, 159.9, 159.7, 156.0 (2C), 127.6 (2C), 127.3, 113.9, 112.1 (2C), 96.3 (2C), 94.3 (2C), 93.9, 63.1, 58.5, 55.8, 55.8 (2C), 55.1. HR-MS, calcd for $C_{22}H_{26}O_9$ ([M+H]$^+$) 435.1650, found 435.1638.

((2*S,3*R**)-3-(4-Nitrophenyl)oxiran-2-yl)(2,4,6-tris(methoxymethoxy)phenyl)methanone ((**±**)-4c)** yield: 89%, white solid. m.p. 86-88 ^{o}C. Rf = 0.37 (EtOAc/*n*-hexane = 1/3 v/v). ^{1}H NMR (500 MHz, DMSO-d_6) δ 8.24 (d, *J* = 9.0 Hz, 2H), 7.66 (d, *J* = 9.0 Hz, 2H), 6.50 (s, 2H), 5.20 (s, 2H), 5.16 (d, *J* = 7.0 Hz, 2H), 5.12 (d, *J* = 7.0 Hz, 2H), 4.25 (d, *J* = 2.0 Hz, 1H), 4.21 (d, *J* = 2.0 Hz, 1H), 3.38 (s, 3H), 3.28 (s, 6H). ^{13}C NMR (126 MHz, DMSO-d_6) δ 194.8, 160.2, 156.3 (2C), 147.6, 143.4, 127.3 (2C), 123.6 (2C), 112.0, 96.4 (2C), 94.4 (2C), 93.9, 63.3, 57.4, 55.9 (3C). HR-MS, calcd for $C_{21}H_{23}NO_{10}$ ([M+H]$^+$) 450.1395, found 450.1407.

((2*S,3*R**)-3-(4-Bromophenyl)oxiran-2-yl)(2,4,6-tris(methoxymethoxy)phenyl)methanone ((±)-4d)** yield: 81%, yellow oil. Rf = 0.42 (EtOAc/*n*-hexane = 1/3 v/v). ^{1}H NMR (500 MHz, DMSO-d_6) δ 7.59 (d, *J* = 8.5 Hz, 2H), 7.33 (d, *J* = 8.5 Hz, 2H), 6.49 (s, 2H), 5.20 (s, 2H), 5.16 (d, *J* = 7.0 Hz, 2H), 5.11 (d, *J* = 7.0 Hz, 2H), 4.13 (d, *J* = 2.0 Hz, 1H), 4.04 (d, *J* = 2.0 Hz, 1H), 3.38 (s, 3H), 3.28 (s, 6H). ^{13}C NMR (126 MHz, DMSO-d_6) δ 195.5, 160.0, 156.1 (2C), 135.2, 131.4 (2C), 128.3 (2C), 121.9, 112.0, 96.3 (2C), 94.3 (2C), 93.9, 63.1, 57.9, 55.9, 55.8 (2C). HR-MS, calcd for $C_{21}H_{23}BrO_8$ ([M+H]$^+$) 483.0649, found 483.0662.

((2*S,3*R**)-3-(3,5-Bis(methoxymethoxy)phenyl)oxiran-2-yl)(2,4,6-tris(methoxymethoxy)phenyl)methanone ((±)-4e)** yield: 87%, light yellow solid. m.p. 49-50 °C. Rf = 0.32 (EtOAc/*n*-hexane = 1/3 v/v). ^{1}H NMR (500 MHz, DMSO-d_6) δ 6.67 (d, *J* = 2.0 Hz, 2H), 6.65-6.64 (m, 1H), 6.49 (s, 2H), 5.20-5.11 (m, 10H), 4.06 (d, *J* = 2.0 Hz, 1H), 3.95 (d, *J* = 2.0 Hz, 1H), 3.38 (s, 3H), 3.35 (s, 6H), 3.28 (s, 6H). ^{13}C NMR (126 MHz, DMSO-d_6) δ 195.5, 160.0, 157.9 (2C), 156.1 (2C), 138.2, 112.1, 106.8 (2C), 104.9, 96.3 (2C), 94.3 (2C), 93.9, 93.7 (2C), 63.1, 58.4, 55.9, 55.8 (2C), 55.6 (2C). HR-MS, calcd for $C_{25}H_{32}O_{12}$ ([M+H]$^+$) 525.1967, found 525.1949.

((2*S,3*R**)-3-(4-Methoxy-3-(methoxymethoxy)phenyl)oxiran-2-yl)(2,4,6-tris(methoxymethoxy)phenyl)methanone** ((±)-**4f**) yield: 92%, yellow oil. Rf = 0.32 (EtOAc/*n*-hexane = 1/3 v/v). ^{1}H NMR (500 MHz, DMSO-d_6) δ 7.03-6.99 (m, 3H), 6.49 (s, 2H), 5.20 (s, 2H), 5.19-5.10 (m, 6H), 4.08 (d, *J* = 2.0 Hz, 1H), 3.92 (d, *J* = 2.0 Hz, 1H), 3.77 (s, 3H), 3.38 (d, *J* = 6.0 Hz, 6H), 3.29 (s, 6H). ^{13}C NMR (126 MHz, DMSO-d_6) δ 196.1, 159.9, 156.0 (2C), 150.3, 145.9, 127.7, 120.7, 114.0, 112.2, 112.2, 96.3 (2C), 94.7, 94.2 (2C), 93.9, 63.2, 58.5, 55.9, 55.8 (2C), 55.6, 55.6. HR-MS, calcd for $C_{24}H_{30}O_{11}$ ([M+H]$^+$) 495.1861, found 495.1847.

((2*S,3*R**)-3-(2,3-Dihydrobenzo[*b*][1,4]dioxin-6-yl)oxiran-2-yl)(2,4,6-tris(methoxymethoxy)phen yl)methanone ((±)-4g)** yield: 89%, yellow oil. Rf = 0.35 (EtOAc/*n*-hexane = 1/3 v/v). ^{1}H NMR (500 MHz, DMSO-d_6) δ 6.86-6.82 (m, 3H), 6.49 (s, 2H), 5.20 (s, 2H), 5.16 (d, 2H, *J* = 7.0 Hz), 5.12 (d, *J* = 7.0 Hz, 2H), 4.22 (s, 4H), 4.06 (d, *J* = 2.0 Hz, 1H), 3.89 (d, *J* = 2.0 Hz, 1H), 3.38 (s, 3H), 3.30 (s, 6H). ^{13}C NMR (126 MHz, DMSO-d_6) δ 196.1, 159.9, 156.0 (2C), 143.8, 143.4, 128.5, 119.1, 117.1, 114.8, 112.1, 96.3 (2C), 94.3 (2C), 93.9, 64.0, 63.9, 63.0, 58.3, 55.8, 55.8 (2C). HR-MS, calcd for $C_{23}H_{26}O_{10}$ ([M+H]$^+$) 463.1599, found 463.1609.

((2*S,3*R**)-3-(Thiophen-3-yl)oxiran-2-yl)(2,4,6-tris(methoxymethoxy)phenyl)methanone ((±)-4h)** yield: 91%, yellow oil. Rf = 0.40 (EtOAc/*n*-hexane = 1/3 v/v). ^{1}H NMR (500 MHz, DMSO-d_6) δ 7.73-7.72 (m, 1H), 7.56-7.55 (m, 1H), 7.08 (d, *J* = 5.0 Hz, 1H), 6.50 (s, 2H), 5.20 (s, 2H), 5.16 (d, *J* = 7.0 Hz, 2H), 5.12 (d, *J* = 7.0 Hz, 2H), 4.24 (d, *J* = 2.0 Hz, 1H), 4.05 (d, *J* = 2.0 Hz, 1H), 3.38 (s, 3H), 3.29 (s, 6H). ^{13}C NMR (126 MHz, DMSO-d_6) δ 196.4, 159.9, 156.0 (2C), 137.4, 127.1, 125.3 (2C), 112.0, 96.3 (2C), 94.3 (2C), 93.9, 62.5, 55.8, 55.8 (2C), 55.1. HR-MS, calcd for $C_{19}H_{22}O_8S$ ([M+H]$^+$) 411.1108, found 411.1117.

((2*S,3*R**)-3-(3-(2-(Methylthio)ethoxy)phenyl)oxiran-2-yl)(2,4,6-tris(methoxymethoxy)phenyl)methanone ((±)-4i)** yield: 72%, light yellow solid. m.p. 66-67 °C. Rf = 0.39 (EtOAc/*n*-hexane = 1/3 v/v). ^{1}H NMR (500 MHz, DMSO-d_6) δ 7.30-7.27 (m, 1H), 6.97-6.91 (m, 3H), 6.49 (s, 2H), 5.20 (s, 2H), 5.16 (d, *J* = 7.0 Hz, 2H), 5.12 (d, *J* = 7.0 Hz, 2H), 4.18-4.10 (m, 3H), 3.99 (d, *J* = 2.0 Hz, 1H), 3.38 (s, 3H), 3.29 (s, 6H), 2.83 (t, *J* = 6.5 Hz, 2H), 2.14 (s, 3H) . ^{13}C NMR (126 MHz, DMSO-d_6) δ 195.9, 159.9, 158.4, 156.0 (2C), 137.4, 129.6, 118.6, 115.2, 112.1, 111.6, 96.3 (2C), 94.3 (2C), 93.9, 66.9, 63.1, 58.4, 55.8, 55.8 (2C), 32.1, 15.1. HR-MS, calcd for $C_{24}H_{30}O_9S$ ([M+H]$^+$) 495.1683, found 495.1695.

((2*S,3*R**)-3-(3-(2-(Thiophen-2-yl) ethoxy)phenyl)oxiran-2-yl)(2,4,6-tris(methoxymethoxy)phenyl)methanone ((±)-4j)** yield: 71%, colorless oil. Rf = 0.40 (EtOAc/*n*-hexane = 1/3 v/v). ^{1}H NMR (500 MHz, DMSO-d_6) δ 7.35-7.34 (m, 1H), 7.30-7.27 (m, 1H), 6.97-6.93 (m, 5H), 6.49 (s, 2H), 5.20 (s, 2H) , 5.16 (d, *J* = 7.0 Hz, 2H), 5.11 (d, *J* = 7.0 Hz, 2H), 4.21 - 4.16 (m, 2H), 4.13 (s, 1H), 3.99 (s, 1H), 3.38 (s, 3H), 3.29-3.23 (m, 8H). ^{13}C NMR (126 MHz, DMSO-d_6) δ 195.8, 159.9, 158.4, 156.0, 140.3, 137.4, 129.6, 126.7, 125.6, 124.2, 118.6, 115.2, 112.1, 111.7, 96.3, 94.3, 93.9, 67.9, 63.1, 58.4, 55.8, 55.8 (2C), 29.1. HR-MS, calcd for $C_{24}H_{30}O_9S$ ([M+H]$^+$) 531.1683, found 531.1680.

((2*S,3*R**)-3-(4-(4,4,5,5-Tetramethyl-1,3,2-dioxaborolan-2-yl)phenyl)oxiran-2-yl)(2,4,6-tris(methoxymethoxy)phenyl)methanone ((±)-4k)** *t*-BuOLi reacted with **2** for 15 min before the addition of aryl aldehyde, $ZnCl_2$ was not added and reaction time was 15 h, yield: 58%, white solid. m.p. 84-85 °C. Rf = 0.45 (EtOAc/*n*-hexane = 1/3 v/v). ^{1}H NMR (500 MHz, DMSO-d_6) δ 7.67 (d, *J* = 8.0 Hz, 2H), 7.37 (d, *J* = 8.0 Hz, 2H), 6.49 (s, 2H), 5.20 (s, 2H), 5.16 (d, *J* = 7.0 Hz, 2H), 5.12 (d, *J* = 7.0 Hz, 2H), 4.08 (d, *J* = 1.5 Hz, 1H), 4.03 (d, *J* = 1.5 Hz, 1H), 3.38 (s, 3H), 3.28 (s, 6H), 1.29 (s, 12H). ^{13}C NMR (126 MHz, DMSO-d_6) δ 195.6, 160.0, 156.1 (2C), 139.0, 134.5 (2C), 125.4 (2C), 112.0, 96.3 (2C), 94.3 (2C), 93.9, 83.7 (2C), 63.3,

58.3, 55.8, 55.8 (2C), 24.6 (2C), 24.5 (2C). HR-MS, calcd for $C_{27}H_{35}BO_{10}$ ($[M+H]^+$) 531.2396, found 531.2390.

((2*S,3*R**)-3-(4-(Thiomorpholinomethyl)phenyl)oxiran-2-yl)(2,4,6-tris(methoxymethoxy)phenyl)methanone (**(±)-**4l)** $ZnCl_2$ was not added and reaction time was 15 h, yield: 75%, white solid. m.p. 93-95 °C. Rf = 0.31 (EtOAc/*n*-hexane = 1/3 v/v). ^1H NMR (500 MHz, DMSO-d_6) δ 7.32-7.28 (m, 4H), 6.49 (s, 2H), 5.19 (s, 2H), 5.17 (d, *J* = 7.0 Hz, 2H), 5.11 (d, *J* = 7.0 Hz, 2H), 4.09 (d, *J* = 2.0 Hz, 1H), 3.98 (d, *J* = 2.0 Hz, 1H), 3.48 (s, 2H), 3.38 (s, 3H), 3.27 (s, 6H), 2.59 (s, 8H). ^{13}C NMR (126 MHz, DMSO-d_6) δ 195.9, 159.9, 156.0 (2C), 138.8, 134.3, 128.9 (2C), 125.9 (2C), 112.1, 96.3 (2C), 94.3 (2C), 93.9, 63.2, 62.1, 58.4, 55.8, 55.8 (2C), 54.2 (2C), 27.1 (2C). HR-MS, calcd for $C_{26}H_{33}NO_8S$ ($[M+H]^+$) 520.2000, found 520.1997.

Synthesis of (*2R,*3R**)-2-(3,4-dihydroxyphenyl)-3,5,7-trihydroxychroman-4-one (*trans*-(±)-taxifolin)**

To a solution of (±)-**4a** (0.50 g, 0.95 mmol) in the mixed solvent of MeOH (10 mL) and THF (1 mL) was added 40% HBr (2.43 g, 12 mmol), and the reaction mixture was stirred at 45 °C for 1.5 h. On cooling to room temperature, the reaction mixture was diluted with EtOAc (20 mL), washed with saline (20 mL × 2), dried with $MgSO_4$, and concentrated to afford a residue, which was purified via column chromatography (EtOAc/*n*-hexane 1:2, v/v) to give *trans*-(±)-taxifolin (0.20 g, 68%) as white solid. m.p. 227-230 °C. Rf = 0.29 (EtOAc/*n*-hexane = 1/2 v/v). ^1H NMR (500 MHz, DMSO-d_6) δ 11.90 (s, 1H), 10.82 (s, 1H), 9.02 (s, 1H), 8.97 (s, 1H), 6.87 (s, 1H), 6.76-6.72 (m, 2H), 5.90 (d, *J* = 2.0 Hz, 1H), 5.85 (d, *J* = 2.0 Hz, 1H), 5.75 (d, *J* =6.0 Hz, 1H), 4.97 (d, *J* = 11.5 Hz, 1H), 4.51-4.48 (m, 1H). The ^1H NMR data were consistent with those reported [7].

Synthesis of *trans*-(±)-taxifolin on 1-gram scale

Synthesis of the intermediates **1** and **2** have been described above.

To a stirred solution of **2** (1.00 g, 2.60 mmol) in CH_3CN (15 mL) at room temperature under N_2 atmosphere was added **3a** (0.73 g, 3.24 mmol), followed by the successive additions of *t*-BuOLi (0.26 g, 3.24 mmol) and $ZnCl_2$ (37 mg, 0.27 mmol). The reaction mixture was further stirred at room temperature until TLC showed that consumption of **2** was complete. The reaction mixture was diluted with EtOAc (30 mL), washed with saline solution (30 mL), dried with $MgSO_4$ and concentrated. The residue thus obtained was dissolved in the mixed solvent of MeOH (26 mL) and THF (2.6 mL), followed by the addition of 40% HBr (5.22 g). The resulting mixture was then stirred at 45 °C for 1.5 h. On cooling to room temperature, the solution was diluted with EtOAc (50 mL), washed with saline (40 mL × 2), dried with $MgSO_4$, and concentrated to afford a residue, which was purified via column chromatography (EtOAc/*n*-hexane 1:2, v/v) to give *trans*-(±)-taxifolin (0.49 g, total yield from **2**: 44%).

Synthesis of *trans*-(±)-taxifolin on 10-gram scale

Synthesis of the intermediates **1** and **2** have been described above.

To a stirred solution of **2** (10.05 g, 0.026 mol) in CH_3CN (150 mL) at room temperature under N_2 atmosphere was added **3a** (7.33 g, 0.032 mol), followed by the successive additions of *t*-BuOLi (2.61 g, 0.032 mol) and $ZnCl_2$ (0.37 g, 2.70 mmol). The reaction mixture was further stirred at room temperature until TLC show that consumption of **2** was complete. The reaction mixture was diluted with EtOAc (300 mL), washed with saline solution (300 mL), dried with $MgSO_4$ and concentrated. The residue thus obtained was dissolved in the mixed solvent of MeOH (260 mL) and THF (26 mL), followed by the addition of 40% HBr (52.12 g). The resulting mixture was then stirred at 45 °C for 1.5 h. On cooling to room temperature, the solution was diluted with EtOAc (500 mL), washed with saline (400 mL × 2), dried with $MgSO_4$, and concentrated to afford a residue, which was purified via column chromatography (EtOAc/*n*-hexane = 1/2 v/v) to give *trans*-(±)-taxifolin (4.56 g, total yield from **2**: 41%).

Synthesis of *trans*-(±)-taxifolin derivatives on 1-gram scale

Synthesis of the intermediates **1** and **2** have been described above.

General procedure: To a stirred solution of **2** (1.00 g, 2.60 mmol) in CH_3CN (15 mL) at room temperature under N_2 atmosphere was added various aryl aldehydes (3.24 mmol), followed by the successive additions of *t*-BuOLi (0.26 g, 3.24 mmol) and $ZnCl_2$ (37 mg, 0.27 mmol). The reaction solution was stirred at room temperature for 4–6 h until TLC show that consumption of **2** was complete. The reaction mixture was diluted with EtOAc (30 mL), washed with saline (30 mL), dried with $MgSO_4$, and concentrated in vacuo to afford a residue, which was purified via column chromatography (EtOAc/*n*-hexane 1:3, by v/v) to give α,β-epoxycarbonyl intermediates (yield: 70–90%). The intermediate was dissolved in the mixed solvent of MeOH/THF 10:1 (v/v), followed by the addition of 40% HBr (12.0 equiv). The resulting mixture was then stirred at 45 °C for 1.5 h. On cooling to room temperature, the reaction mixture was diluted with EtOAc (40–60 mL), washed with saline (40 mL × 2), dried with $MgSO_4$, and concentrated in vacuo to afford a residue, which was purified via column chromatography (EtOAc/*n*-hexane 1:2, v/v) to give the target compounds (yield: 40–60%).

(2*R,3*R**)-2-(3,5-Dihydroxyphenyl)-3,5,7-trihydroxychroman-4-one ((**±**)-TD-1)** total yield of the whole route: 22%, white solid. m.p. 264-265 °C. Rf = 0.31 (EtOAc/*n*-hexane = 1/2 v/v). ^{1}H NMR (500 MHz, DMSO-d_6) δ 11.87 (s, 1H), 10.84 (s, 1H), 9.30 (s, 2H), 6.33 (d, *J* =2.0 Hz, 2H), 6.20 (t, *J* = 2.0 Hz, 1H), 5.91 (d, *J* = 2.0 Hz, 1H), 5.88 (d, *J* = 2.0 Hz, 1H), 5.83 (d, *J* = 6.5 Hz, 1H), 4.98 (d, *J* = 11.0 Hz, 1H), 4.45-4.41 (m, 1H). The ^{1}H NMR data were consistent with those reported [8].

(2*R,3*R**)-3,5,7-Trihydroxy-2-(3-hydroxy-4-methoxyphenyl)chroman-4-one ((**±**)-TD-2)** total yield of the whole route: 27%, white solid. m.p. 135-137 °C. Rf = 0.30 (EtOAc/*n*-hexane = 1/2 v/v). ^{1}H NMR (500 MHz, DMSO-d_6) δ 11.89 (s, 1H), 10.82 (s, 1H), 9.04 (s, 1H), 6.94-6.92 (m, 2H), 6.89-6.87 (m, 1H), 5.91 (d, *J* = 2.0 Hz, 1H), 5.87 (d, *J* = 2.0 Hz, 1H), 5.78 (d, *J* = 6.0 Hz, 1H), 5.03 (d, *J* = 11.0 Hz, 1H), 4.53-4.50 (m, 1H), 3.78 (s, 3H). The ^{1}H NMR data were consistent with those reported [9].

(2*R,3*R**)-3,5,7-Trihydroxy-2-(3-(2-(methylthio)ethoxy)phenyl)chroman-4-one ((**±**)-TD-3)** total yield of the whole route: 20%, white solid. m.p. 94-95 °C. Rf = 0.33 (EtOAc/*n*-hexane = 1/2 v/v). ^{1}H NMR (500 MHz, DMSO-d_6) δ 11.89 (s, 1H), 10.86 (s, 1H), 7.32 (t, *J* = 7.8 Hz, 1H), 7.12-7.08 (m, 2H), 6.98-6.96 (m, 1H), 5.93 (d, *J* = 2.0 Hz, 1H), 5.89 (d, *J* = 2.0 Hz, 1H), 5.84 (d, *J* = 6.0 Hz, 1H), 5.15 (d, *J* = 12.0 Hz, 1H),

4.66-4.63 (m, 1H), 4.17, (t, *J* = 6.5 Hz, 2H), 2.86 (t, *J* = 6.5 Hz, 2H), 2.16 (s, 3H). ^{13}C NMR (126 MHz, DMSO-*d*$_6$) δ 197.5, 166.7, 163.2, 162.3, 156.0, 138.7, 129.2, 120.4, 114.5, 114.2, 100.3, 96.0, 95.0, 82.6, 71.3, 66.9, 32.1, 15.1. HR-MS, calcd for $C_{18}H_{18}O_6S$ ($[M+H]^+$) 363.0897, found 363.0905.

(2*R,3*R**)-3,5,7-Trihydroxy-2-(3-(2-(thiophen-2-yl)ethoxy)phenyl)chroman-4-one ((±)-TD-4)** total yield of the whole route: 26%, white solid. m.p. 116-118 ^{o}C. Rf = 0.35 (EtOAc/*n*-hexane = 1/2 v/v). ^{1}H NMR (500 MHz, DMSO-*d*$_6$) δ 11.89 (s, 1H), 10.85 (s, 1H), 7.36-7.31 (m, 2H), 7.12 (s, 1H), 7.09 (d, *J* = 8.0 Hz, 1H), 6.99-6.96 (m, 3H), 5.93 (d, *J* = 2.0 Hz, 1H), 5.89 (d, *J* = 2.0 Hz, 1H), 5.84 (d, *J* = 6.0 Hz, 1H), 5.15 (d, *J* = 11.0 Hz, 1H), 4.66-4.62 (m, 1H), 4.20 (t, *J* = 6.5 Hz, 2H), 3.27 (t, *J* = 6.5 Hz, 2H). ^{13}C NMR (126 MHz, DMSO-*d*$_6$) δ 197.5, 166.7, 163.2, 162.3, 158.1, 140.4, 138.7, 129.2, 126.8, 125.6, 124.2, 120.5, 114.5, 114.2, 100.4, 96.0, 95.0, 82.7, 71.3, 68.0, 29.2. HR-MS, calcd for $C_{21}H_{18}O_6S$ ($[M+H]^+$) 399.0897, found 399.0893.

References

1. Jr, B. R.; Fink, R. C.; Mcmichael, M. D.; Li, D.; Alberte, R. S. *Phytochemistry*. **2009**, *70*, 1255-1261. doi: 10.1016/j.phytochem.2009.06.003.
2. Kim, B. T.; O. K. J.; Chun, J. C.; Hwang, K. J. *Bulletin of the Korean Chemical Society*. **2008**, *29*, 1125-1130. doi: 10.5012/bkcs.2008.29.6.1125.
3. Zhang, M.; Jr, G. E. J.; Zandt, M. V.; Beckett, P.; Schroeter, H. *Tetrahedron: Asymmetry*. **2013**, *24*, 362-373. doi: 10.1016/j.tetasy.2013.02.012.
4. Altmann, E.; Beerli, R.; Gerspacher, M.; Renaud, J.; Weiler, S.; Widler, L. Aryl-quinazoline/aryl-2amino-phenyl methanone derivatives. WO Patent: 2004/056365 A2, July 8, **2004**.
5. Chaturvedi, R. N.; Arish, M.; Kashif, M.; Kumar, V.; Reenu.; Pendem, K.; Rub, A.; Malhotra, S. *ChemistrySelect*. **2018**, *3*, 2756-2762. doi: 10.1002/slct.201800056.
6. Hodík, T.; Lamač, M.; Lucie Červenková Šťastná, L. Č.; Cuřínová, P.; Karban, J.; Skoupilová, H.; Hrstka, R.; Císařová, I.; Gyepes, R.; Pinkas, J. *J. Organomet. Chem.* **2017**, *846*, 141-151. doi: 10.1016/j.jorganchem.2017.06.005.
7. Kim, N. C.; Graf, T. N.; Sparacino, C. M.; Wani, M. C.; Wall, M. E. *Org. Biomol. Chem*. **2003**, *1*, 1684-1689. doi: 10.1039/B300099K.
8. Zhang, Y.; Que, S.; Yang, X.; Wang, B.; Qiao, L.; Zhao, Y. *Magn. Reson. Chem*. **2007**, *45*, 909-916. doi: 10.1002/mrc.2051.
9. Nessa, F.; Ismail, Z.; Mohamed, N.; Haris, M. *Food Chem*. **2004**, *88*, 243-252. doi: 10.1016/j.foodchem.2004.01.041.

^{1}H NMR spectrum of compound **3e**

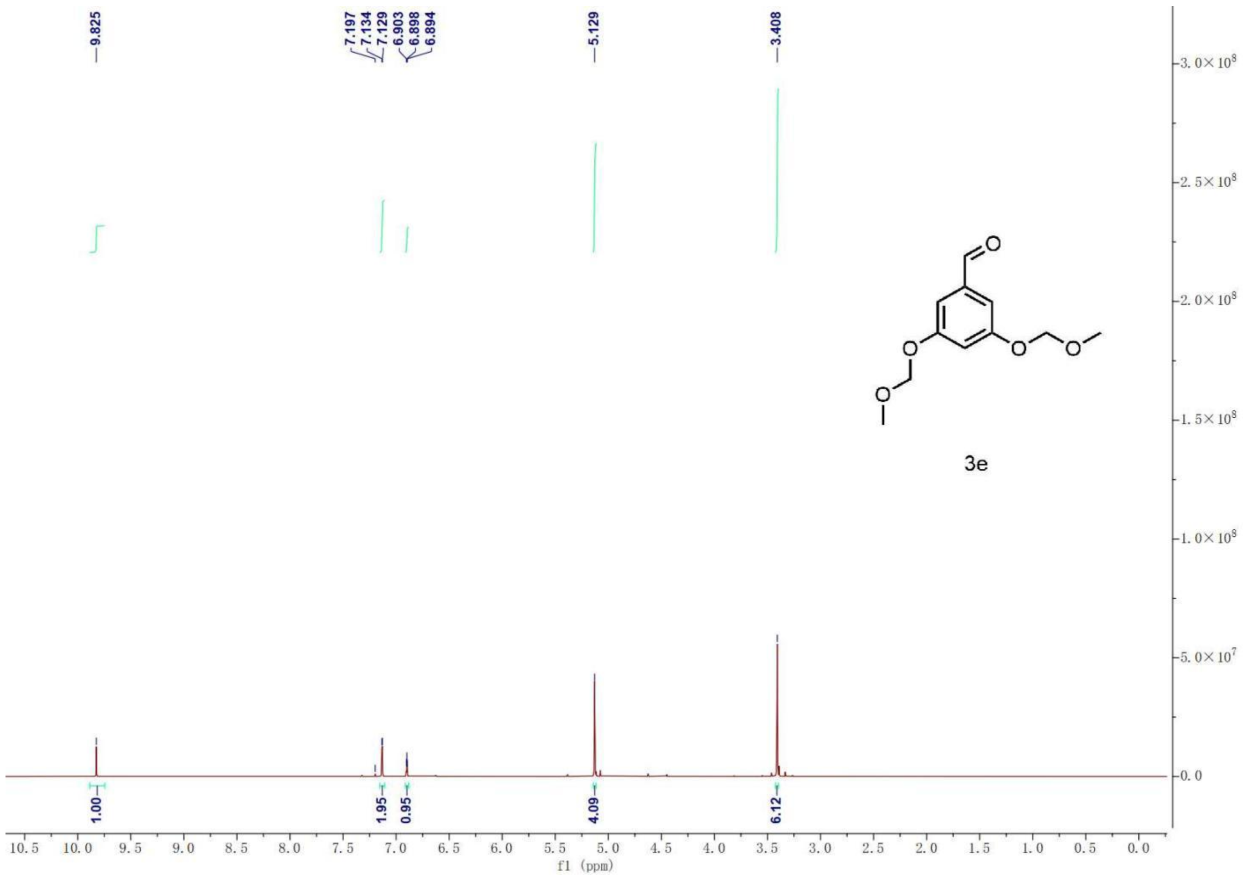

^{1}H NMR spectrum of compound **3f**

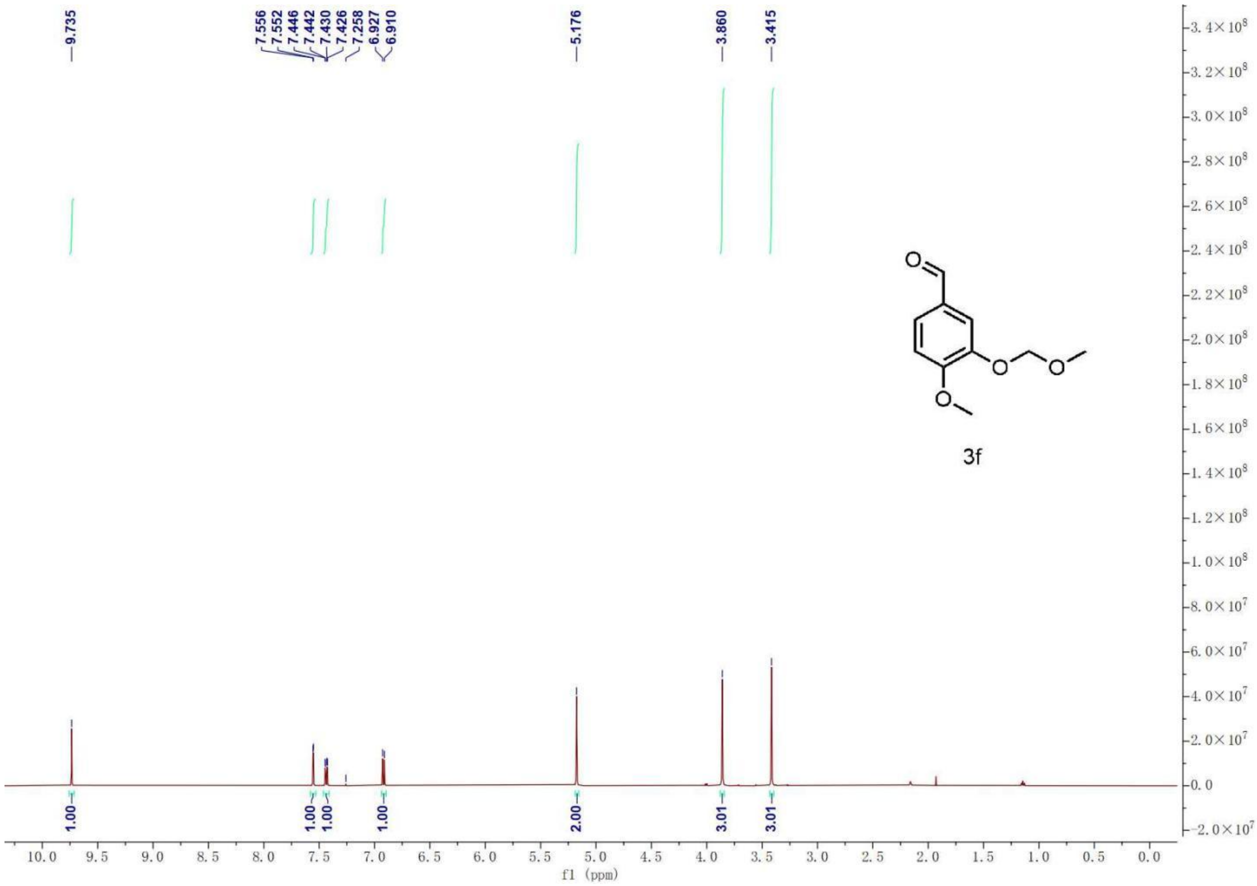

^{1}H NMR spectrum of compound **3i**

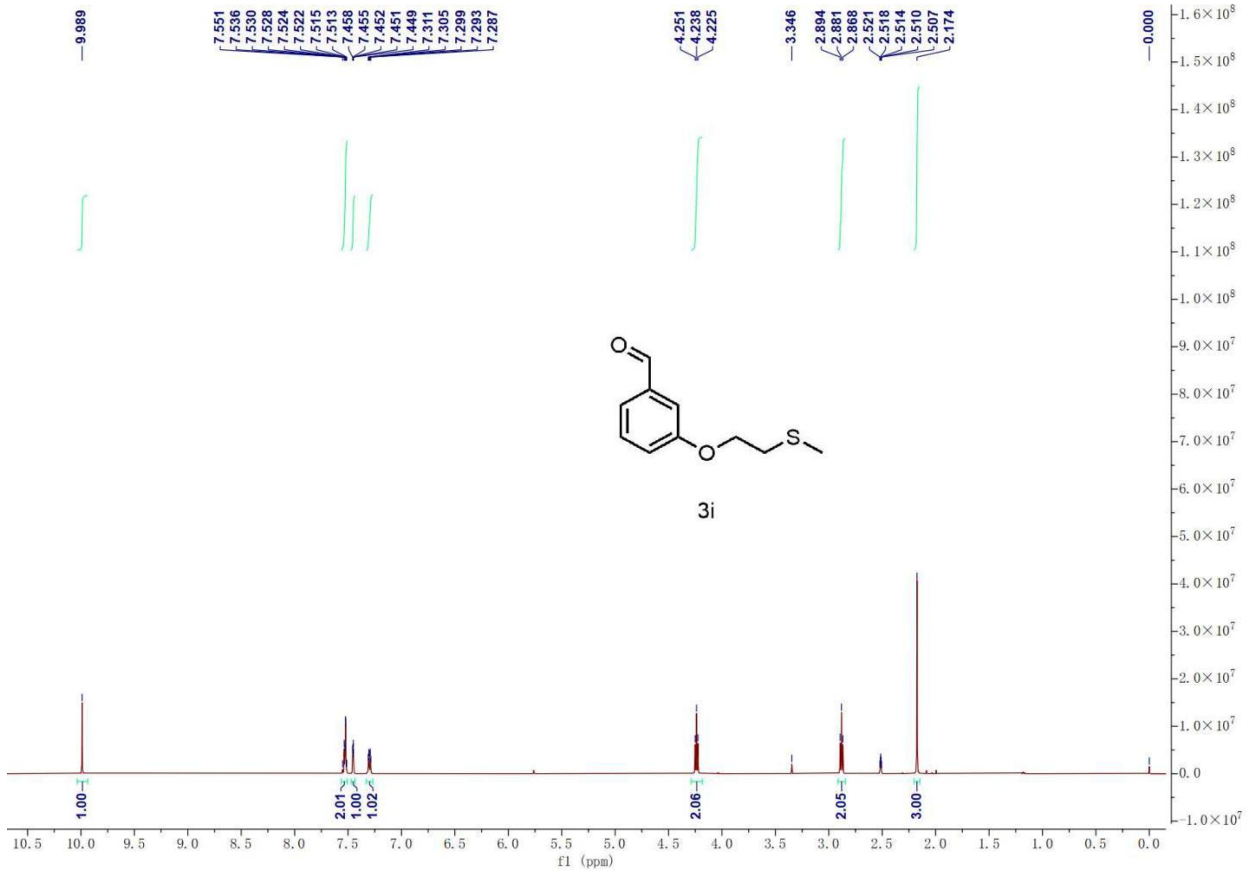

^{1}H NMR spectrum of compound **3j**

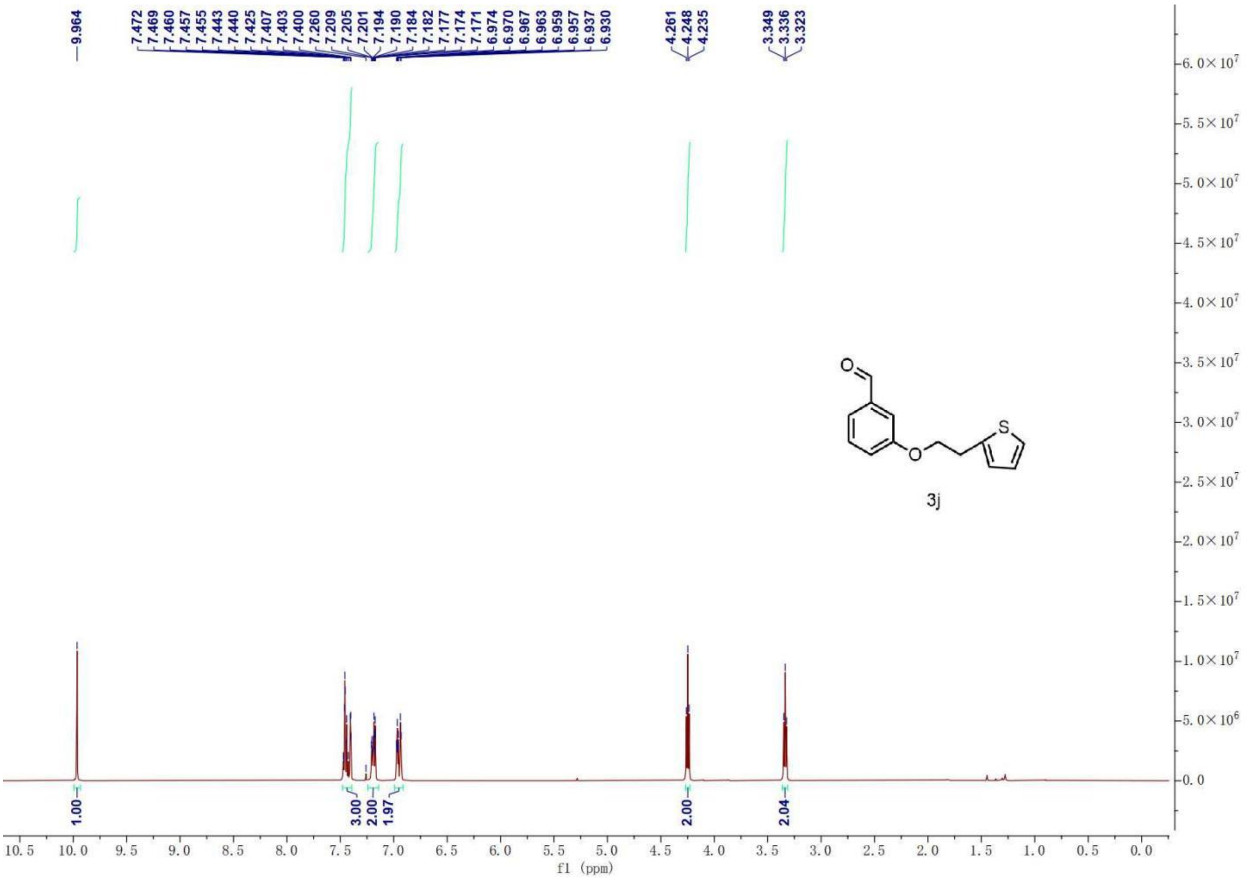

^{1}H NMR spectrum of compound **3l**

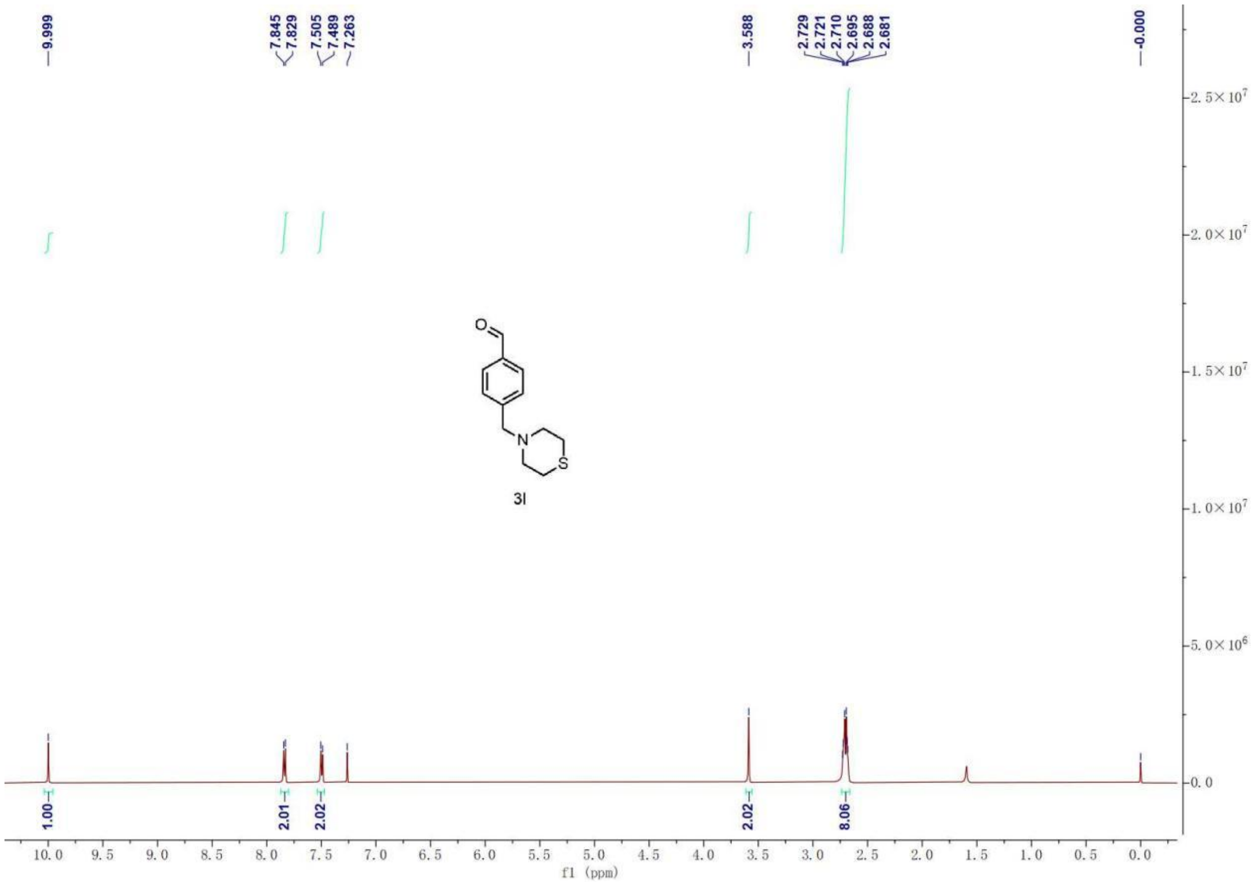

^{1}H NMR spectrum of compound **1**

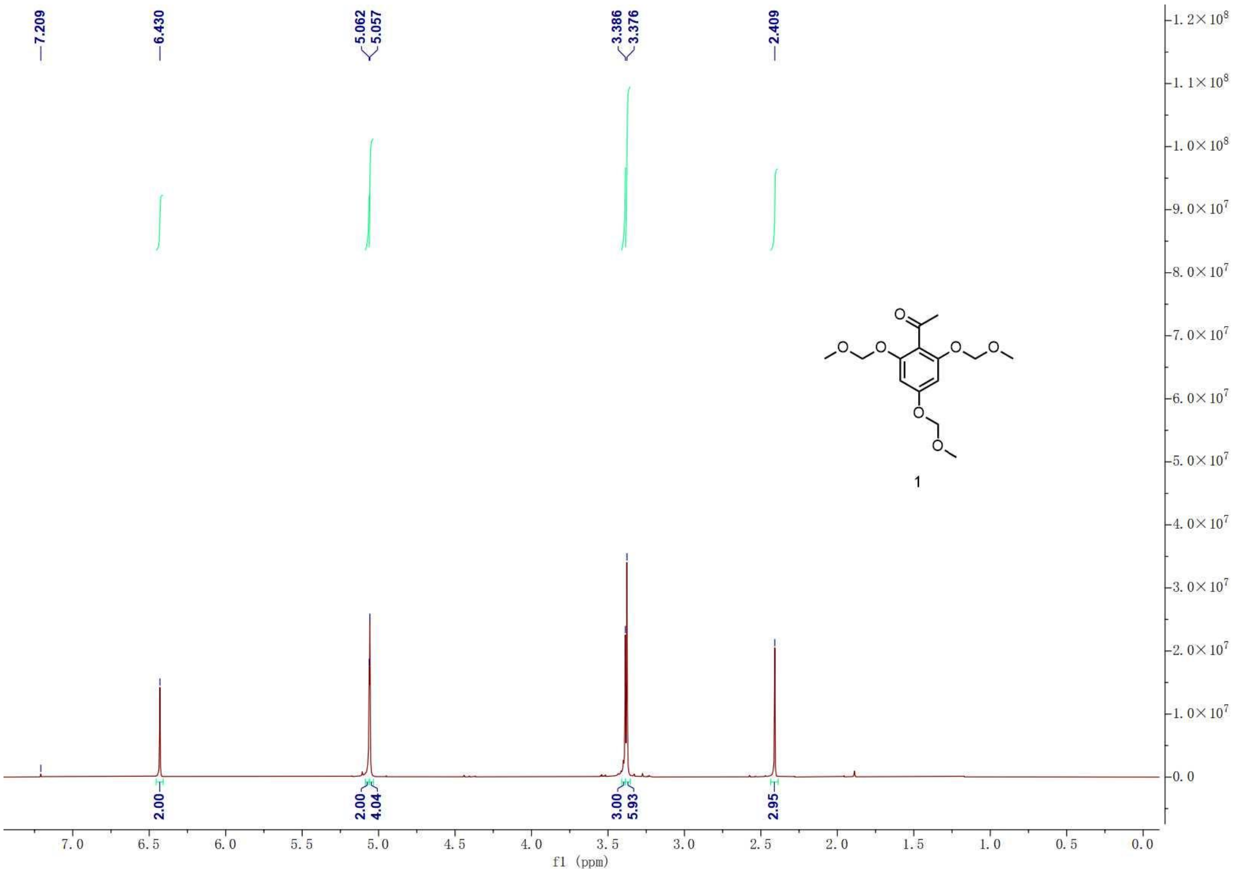

^{1}H NMR spectrum of compound **2**

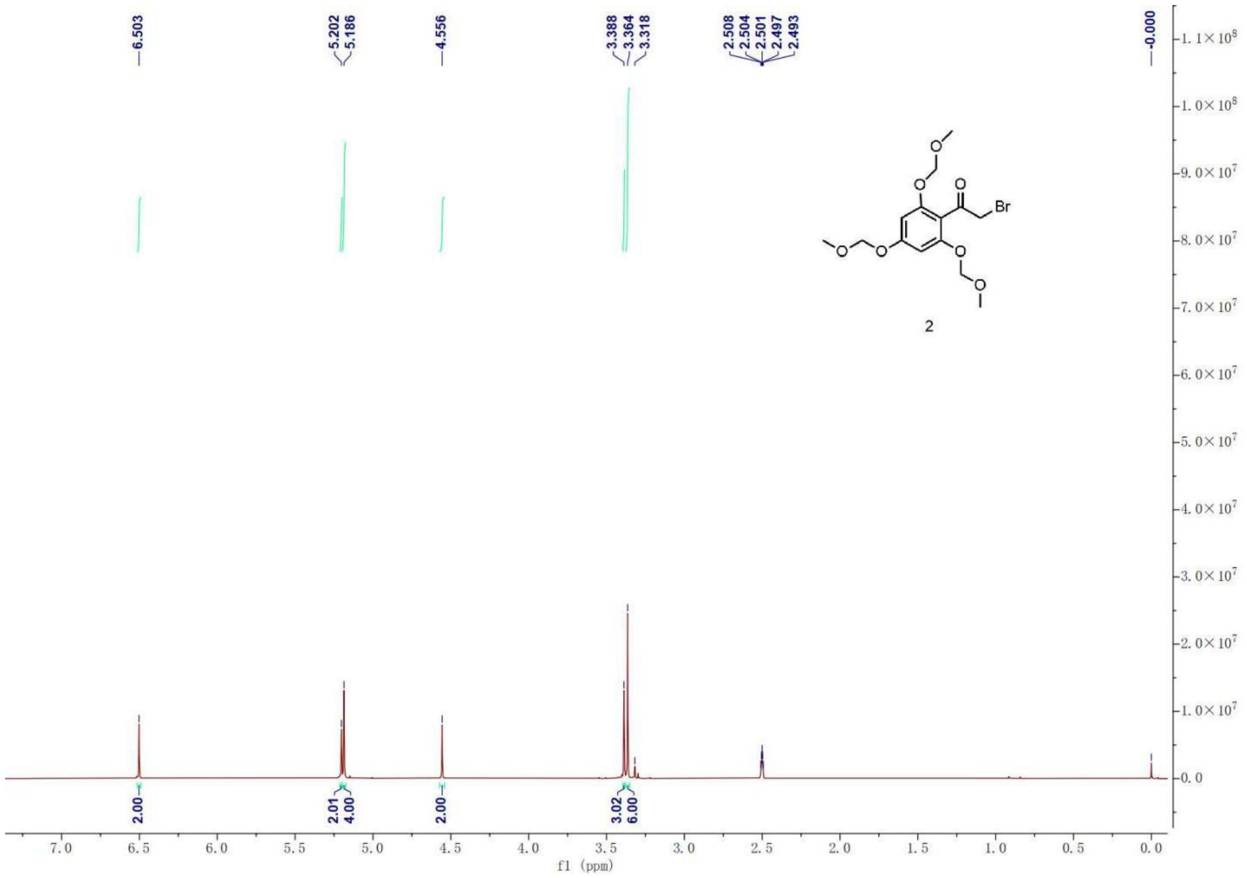

^{13}C NMR spectrum of compound **2**

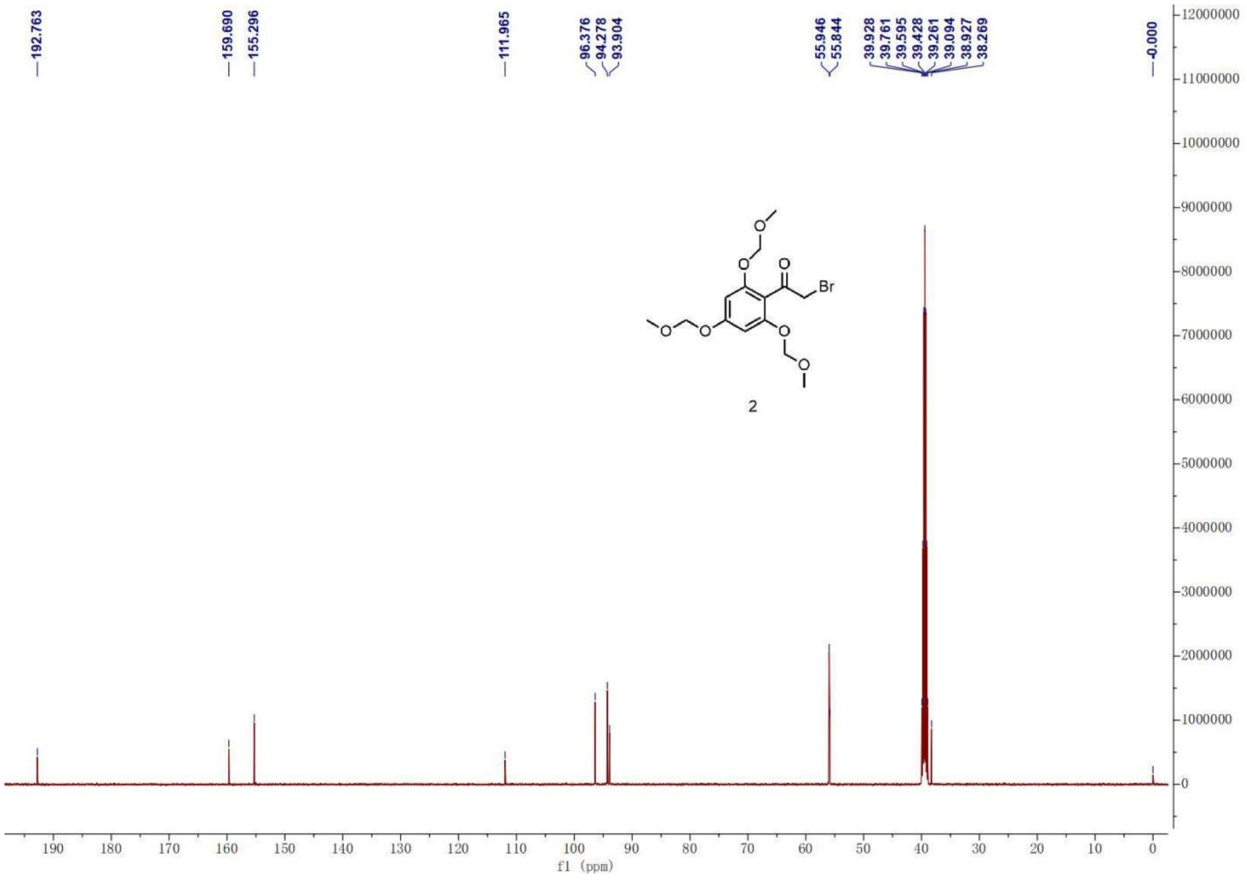

HRMS spectrum of compound **2**

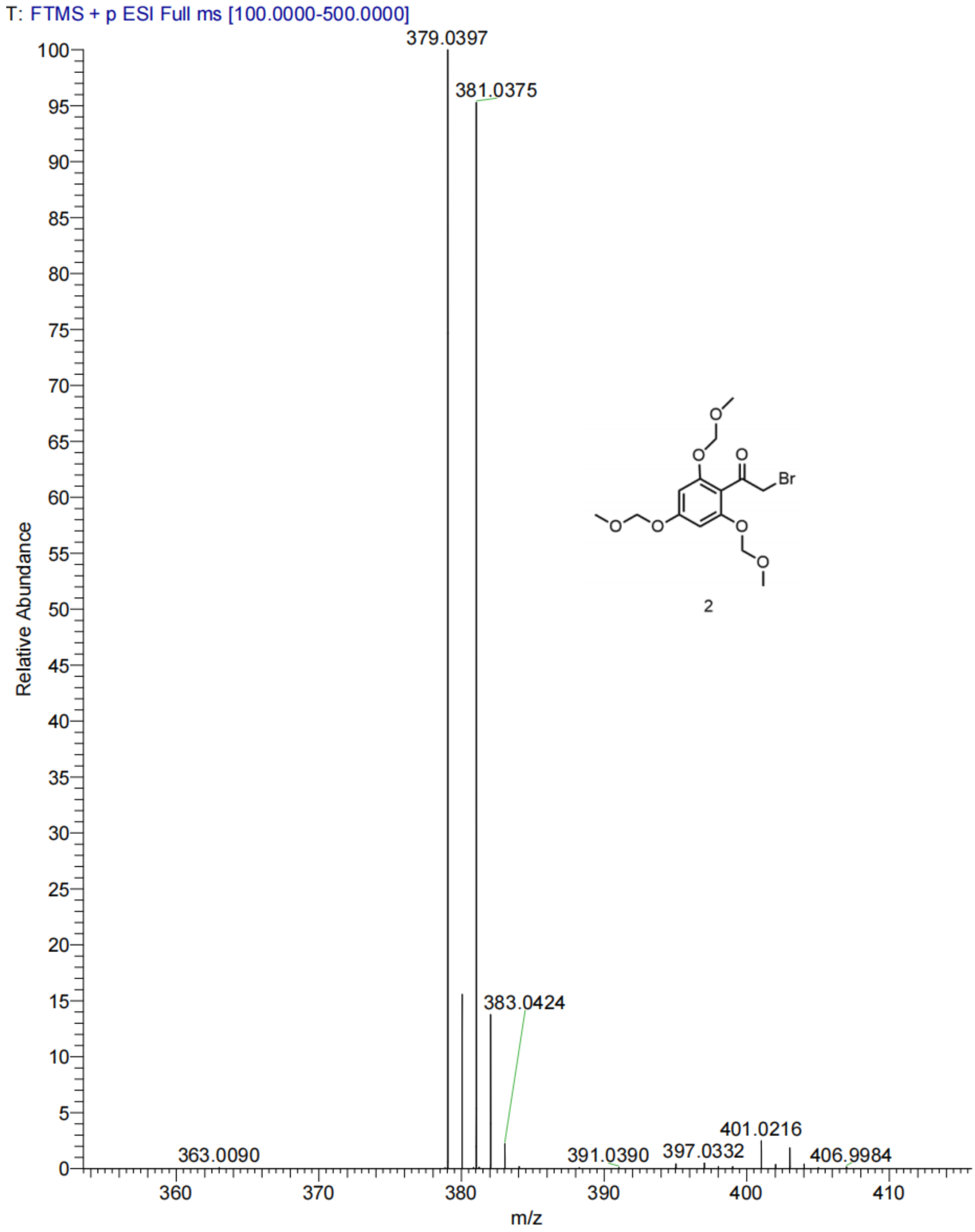

^{1}H NMR spectrum of compound (±)-**4a**

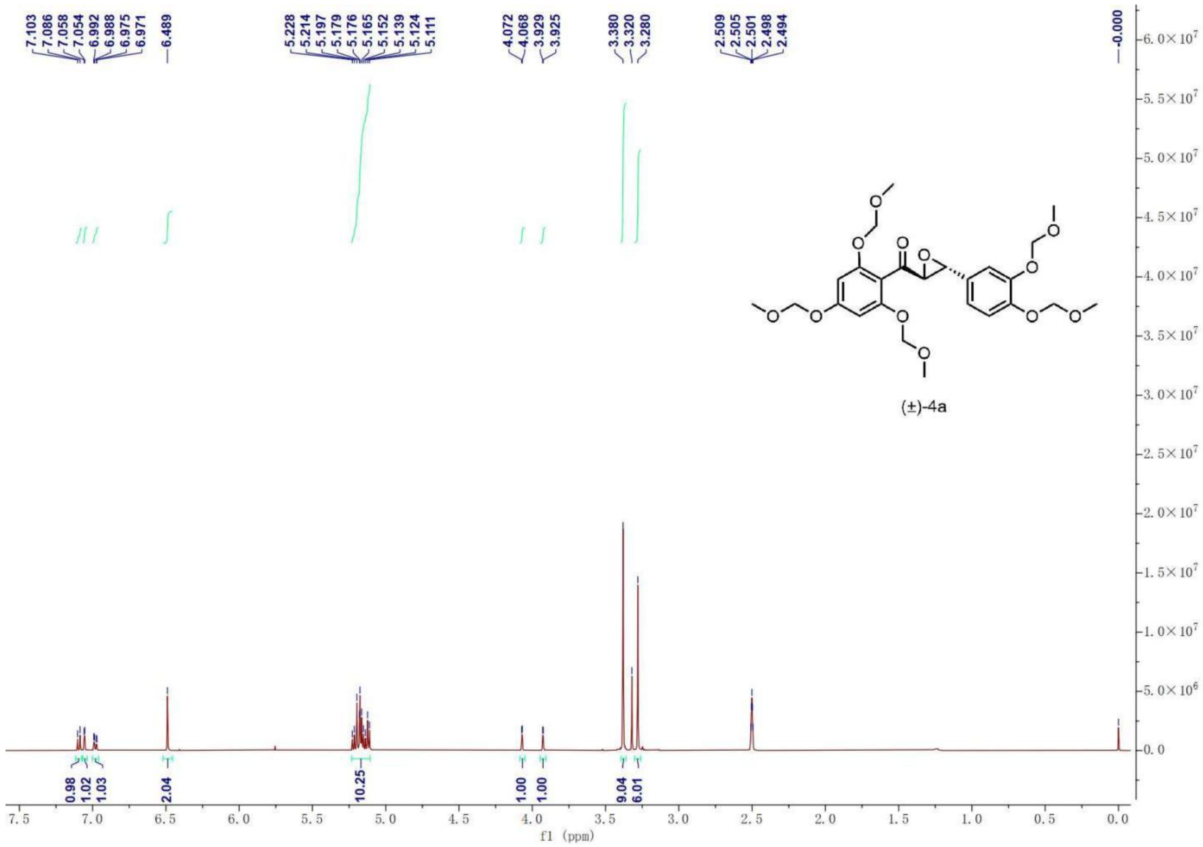

^{13}C NMR spectrum of compound (±)-**4a**

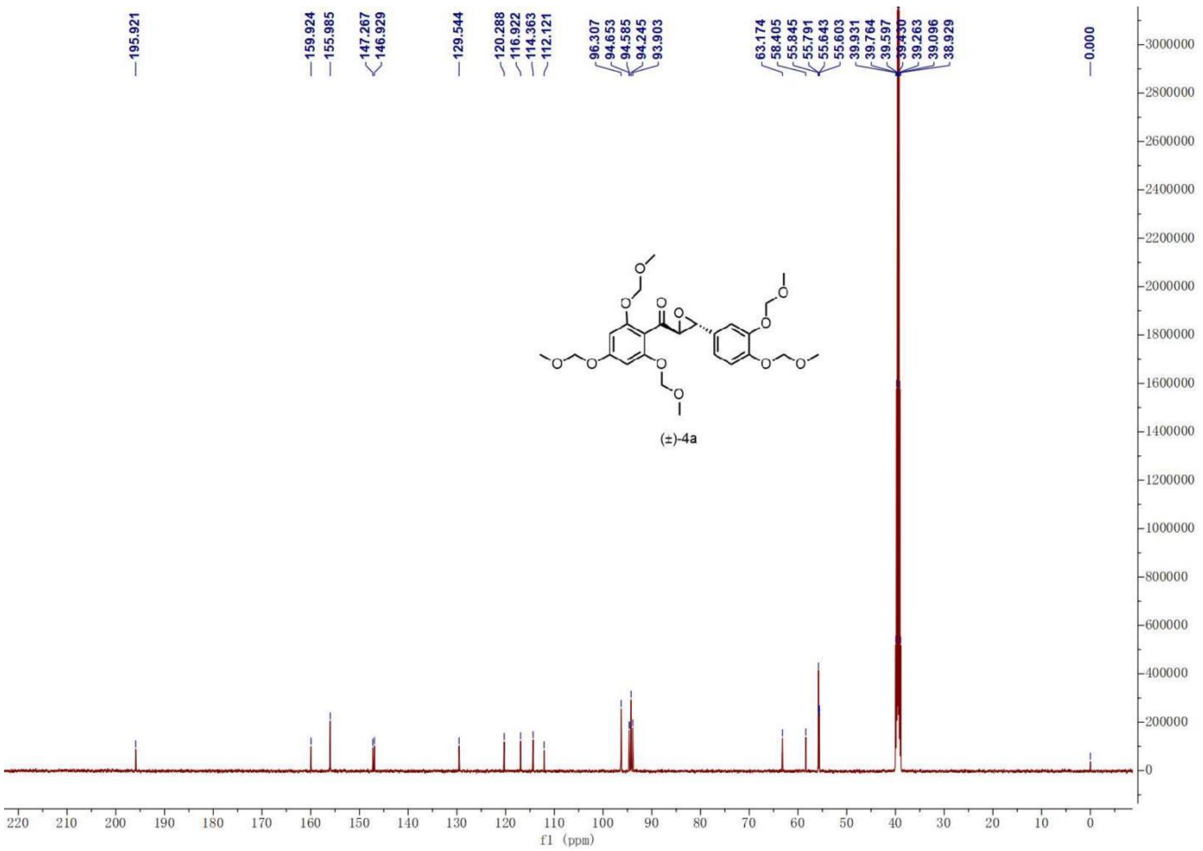

HRMS spectrum of compound (±)-**4a**

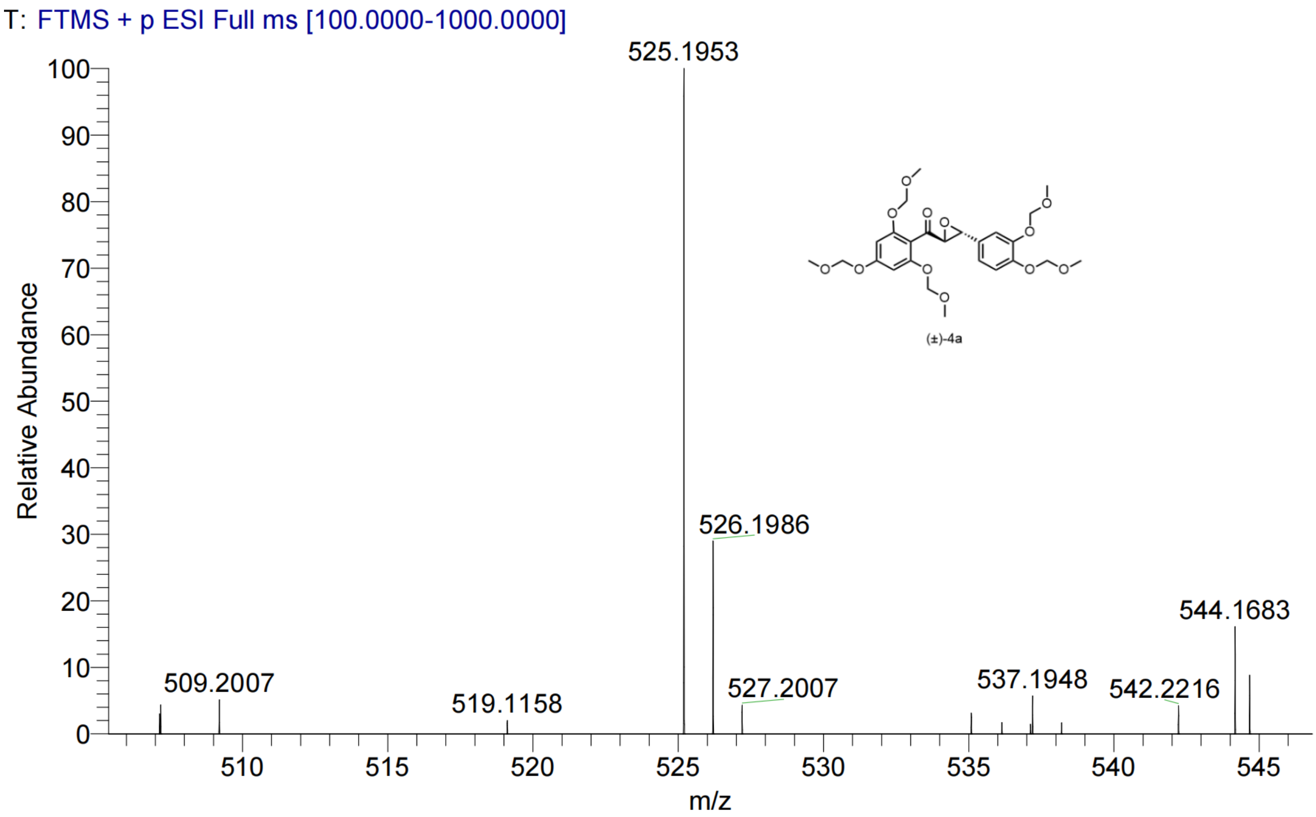

^{1}H NMR spectrum of compound (±)-**4b**

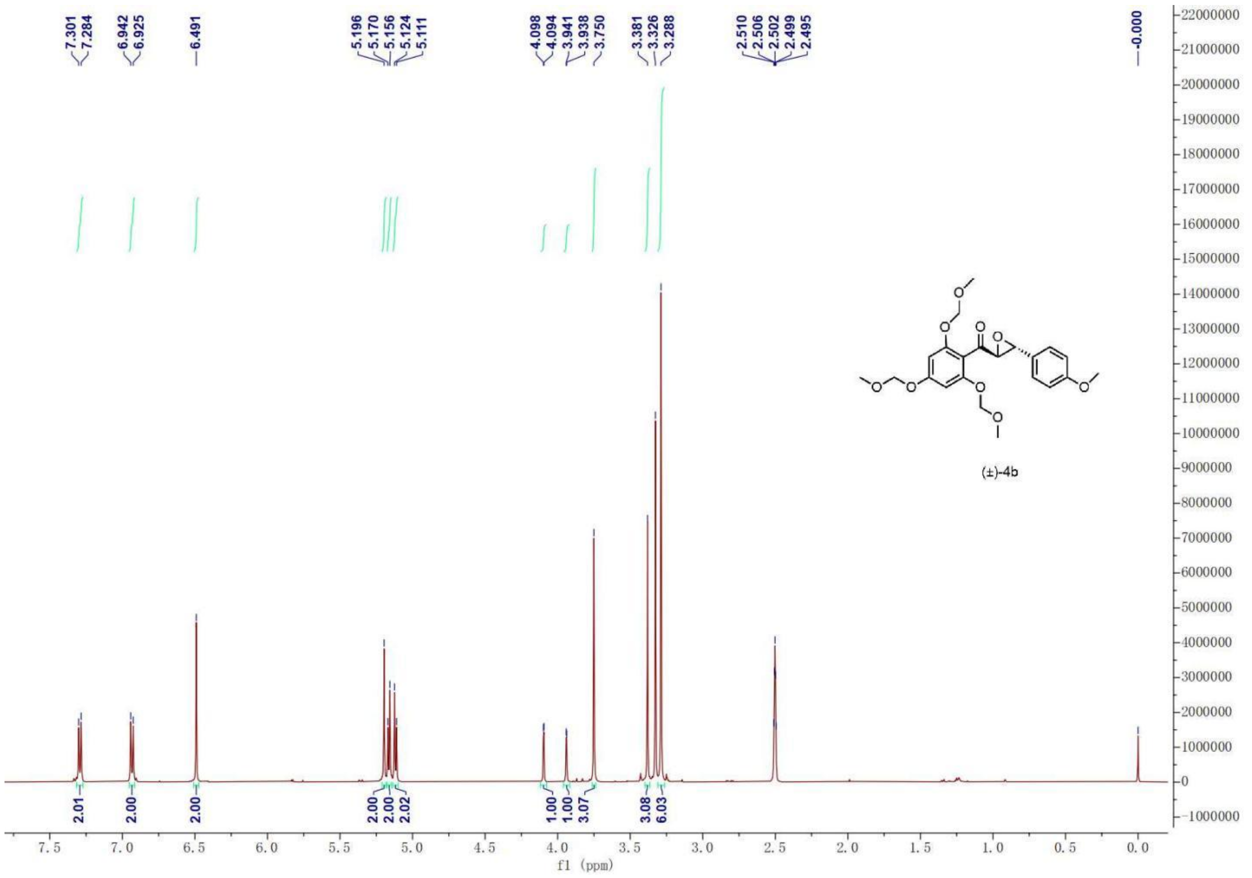

^{13}C NMR spectrum of compound (±)-**4b**

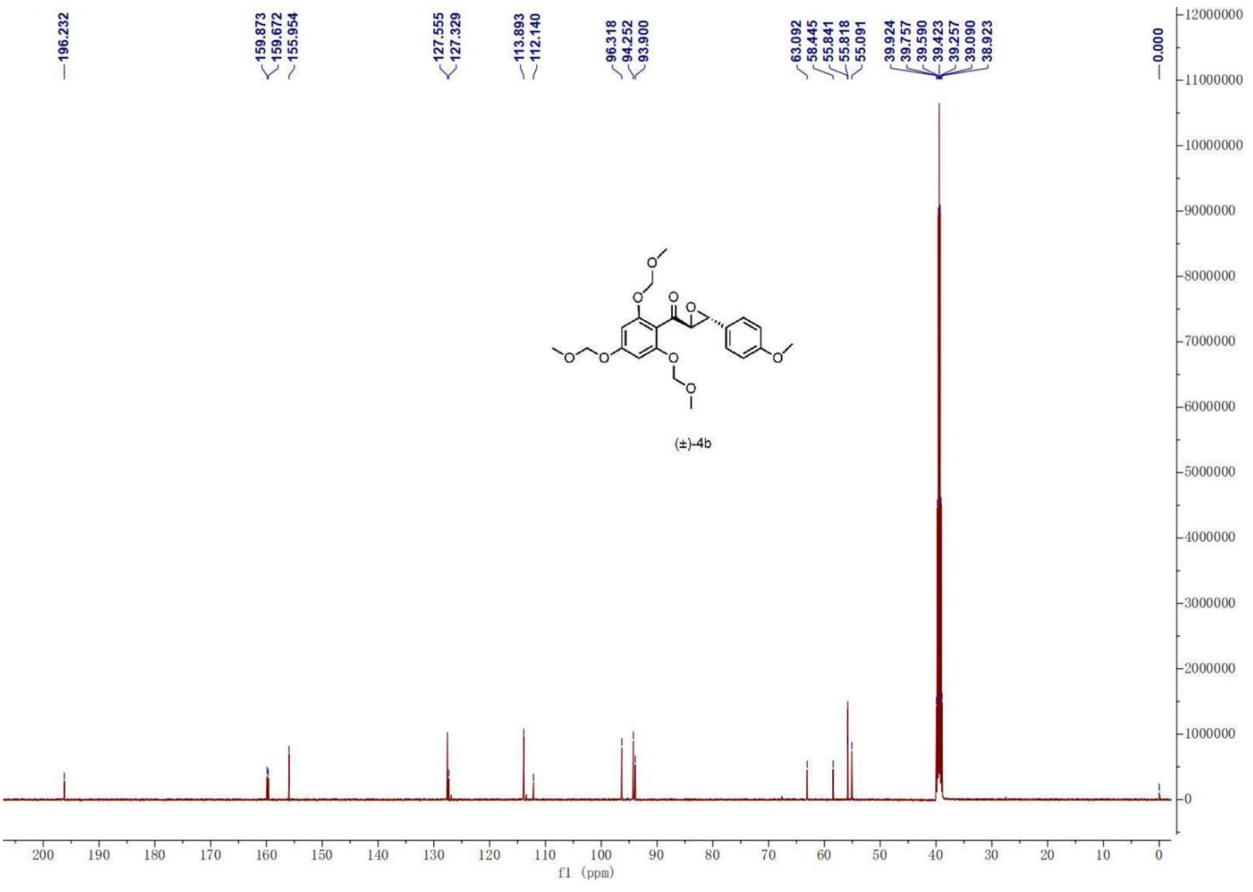

HRMS spectrum of compound (±)-**4b**

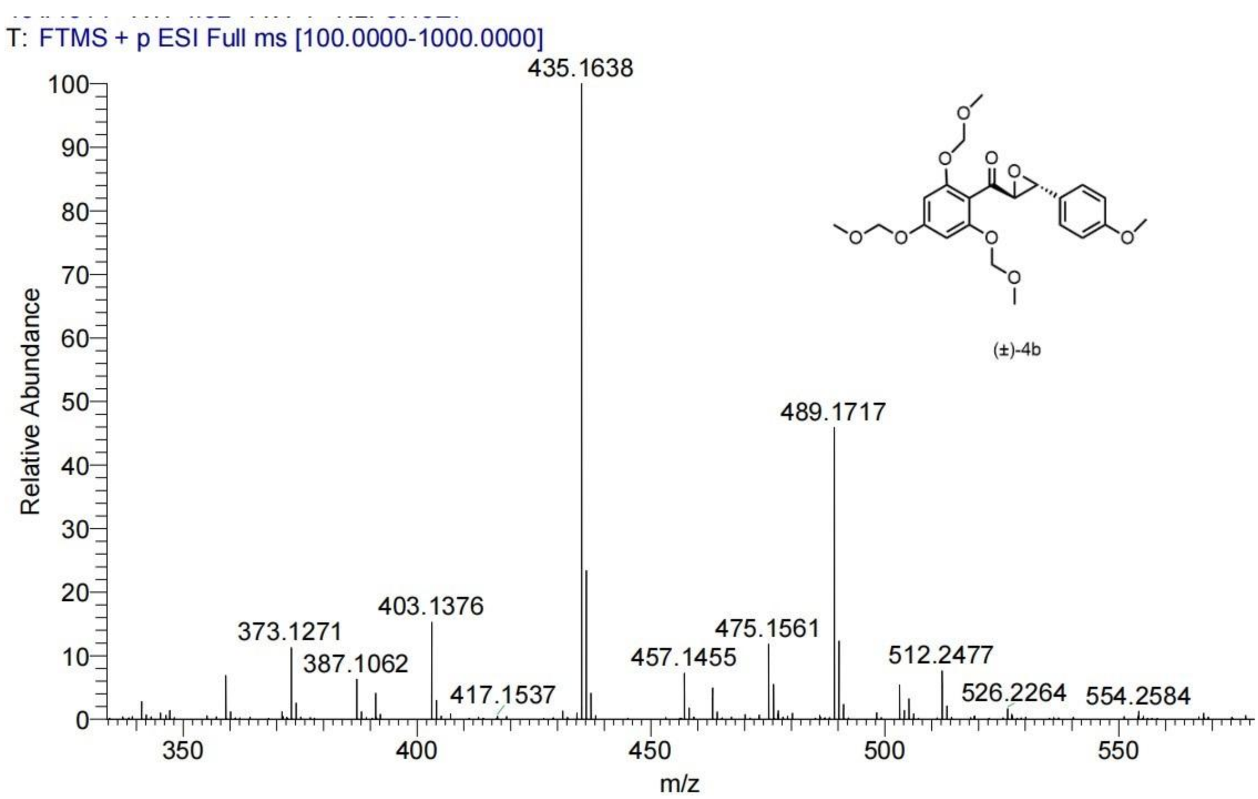

^{1}H NMR spectrum of compound (±)-**4c**

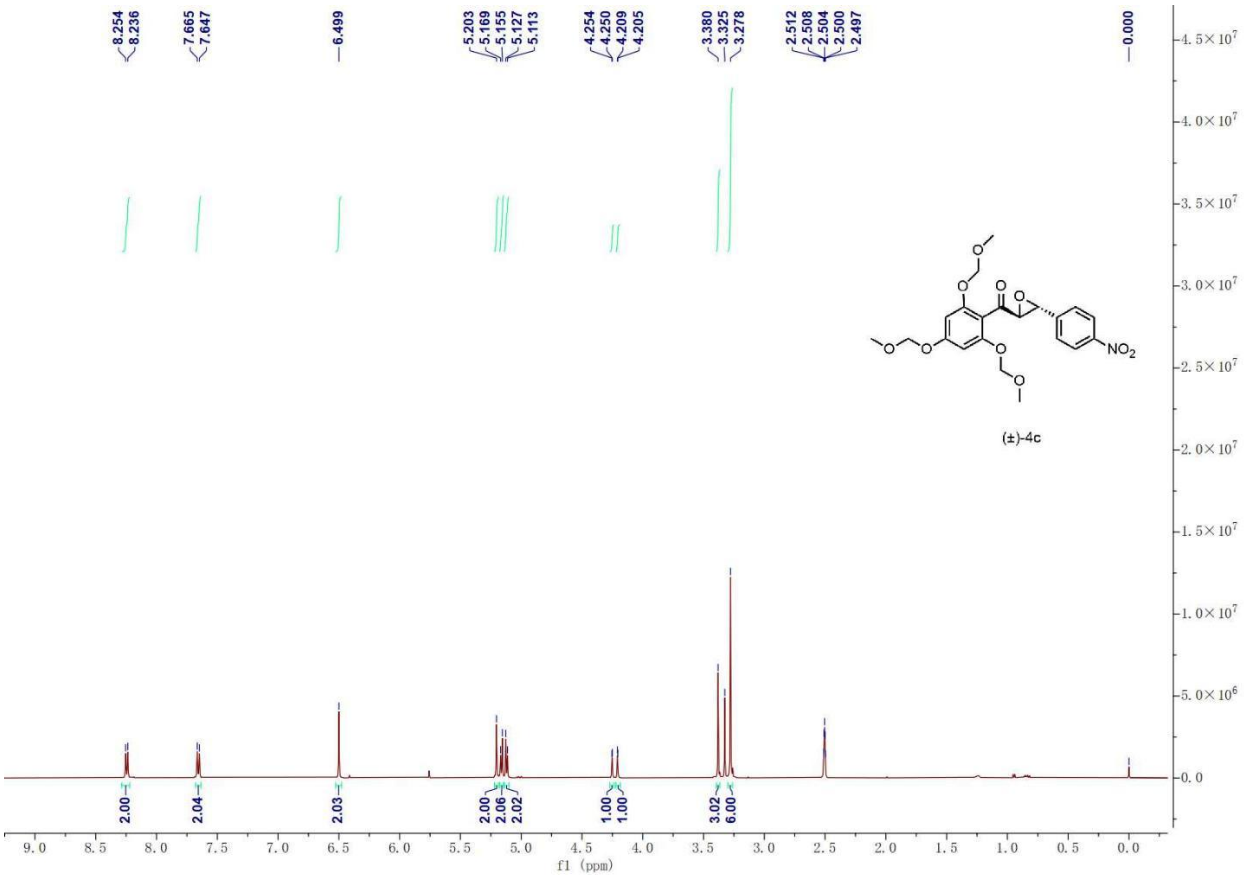

^{13}C NMR spectrum of compound (±)-**4c**

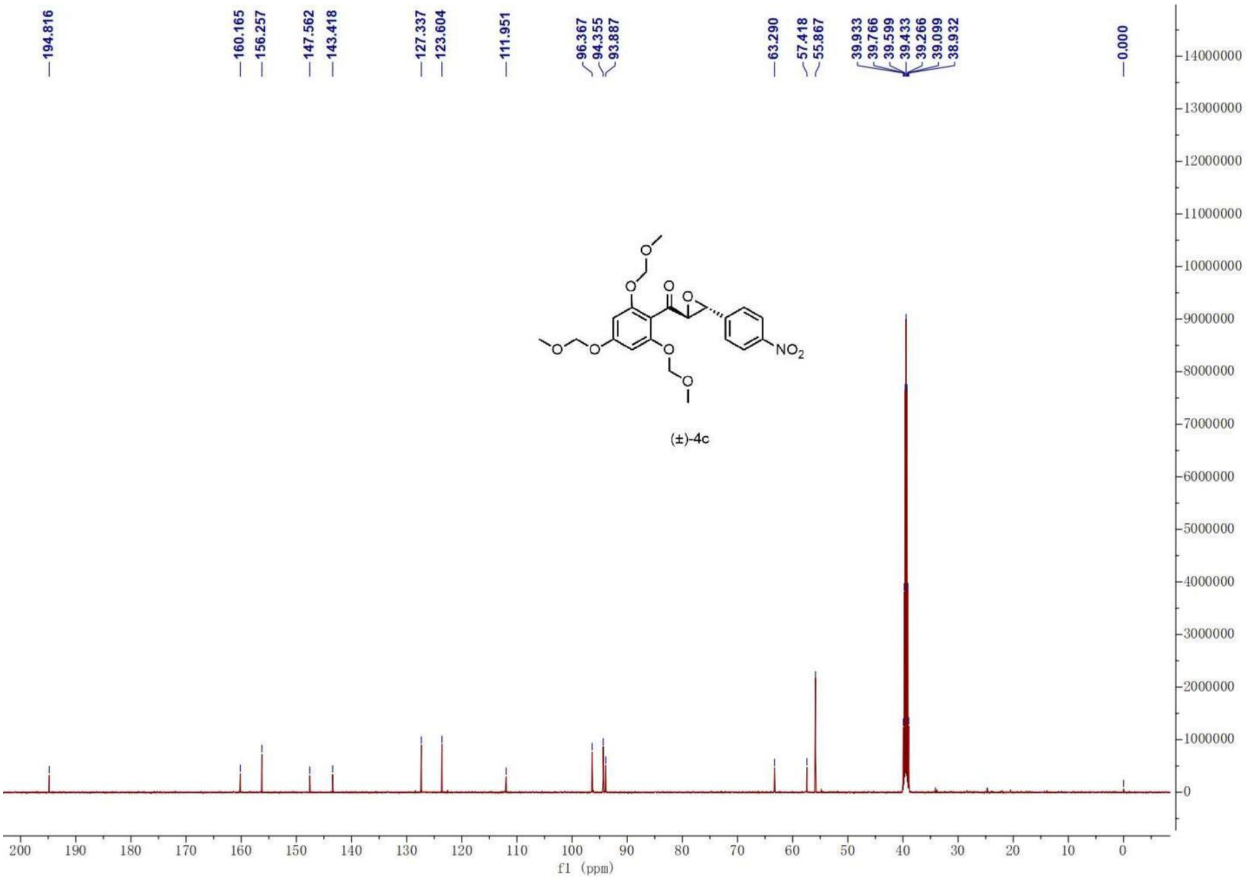

HRMS spectrum of compound (±)-**4c**

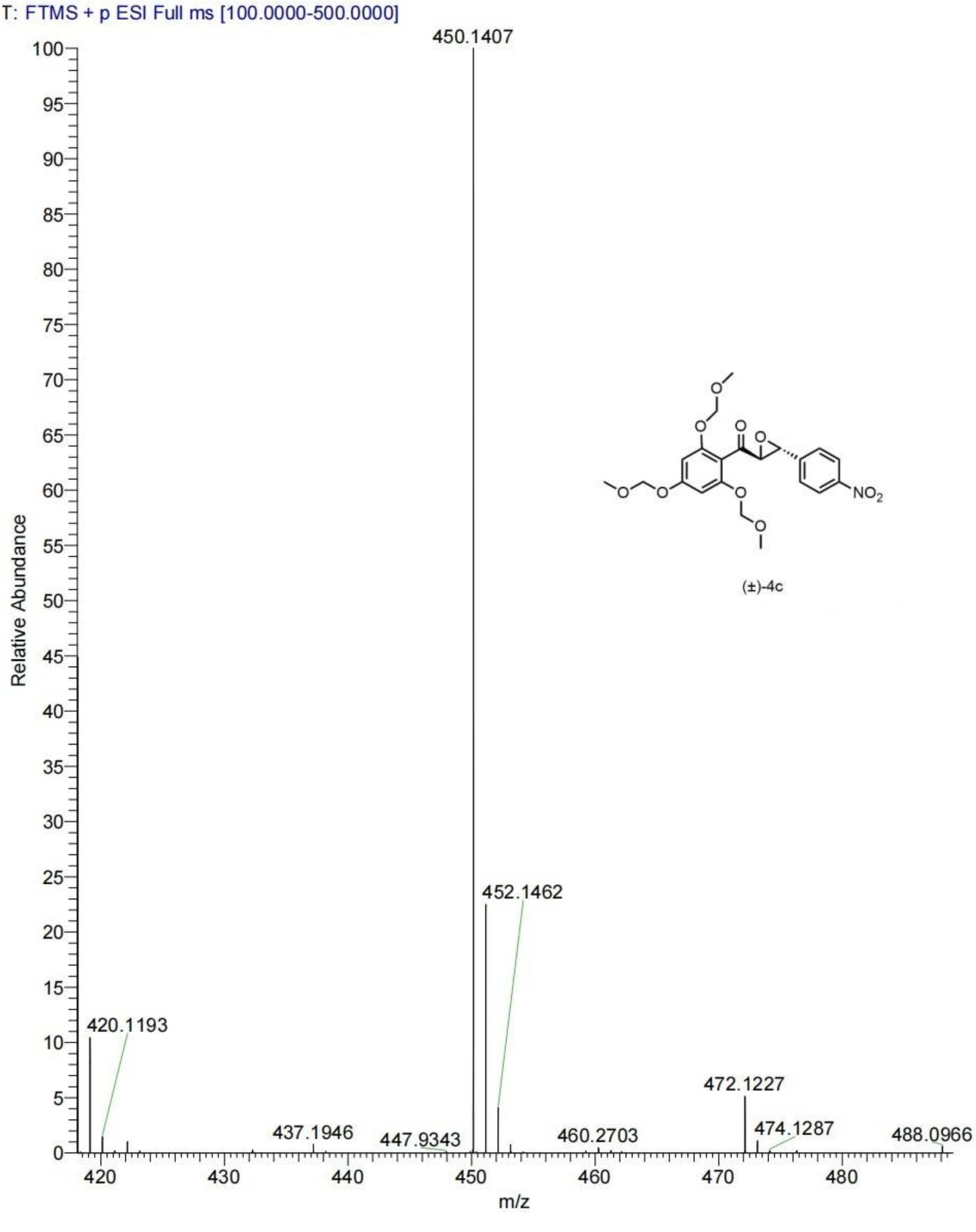

^{1}H NMR spectrum of compound (±)-**4d**

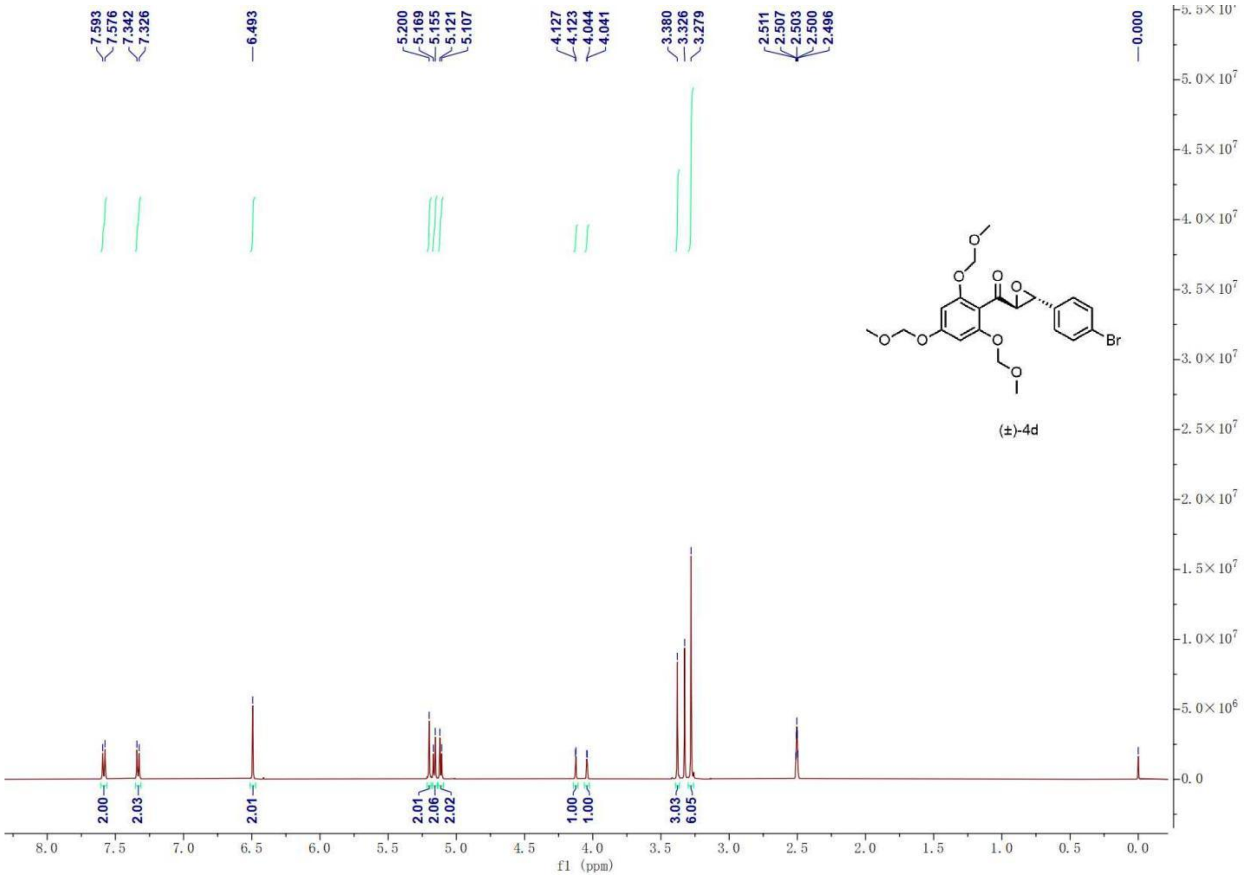

^{13}C NMR spectrum of compound (±)-**4d**

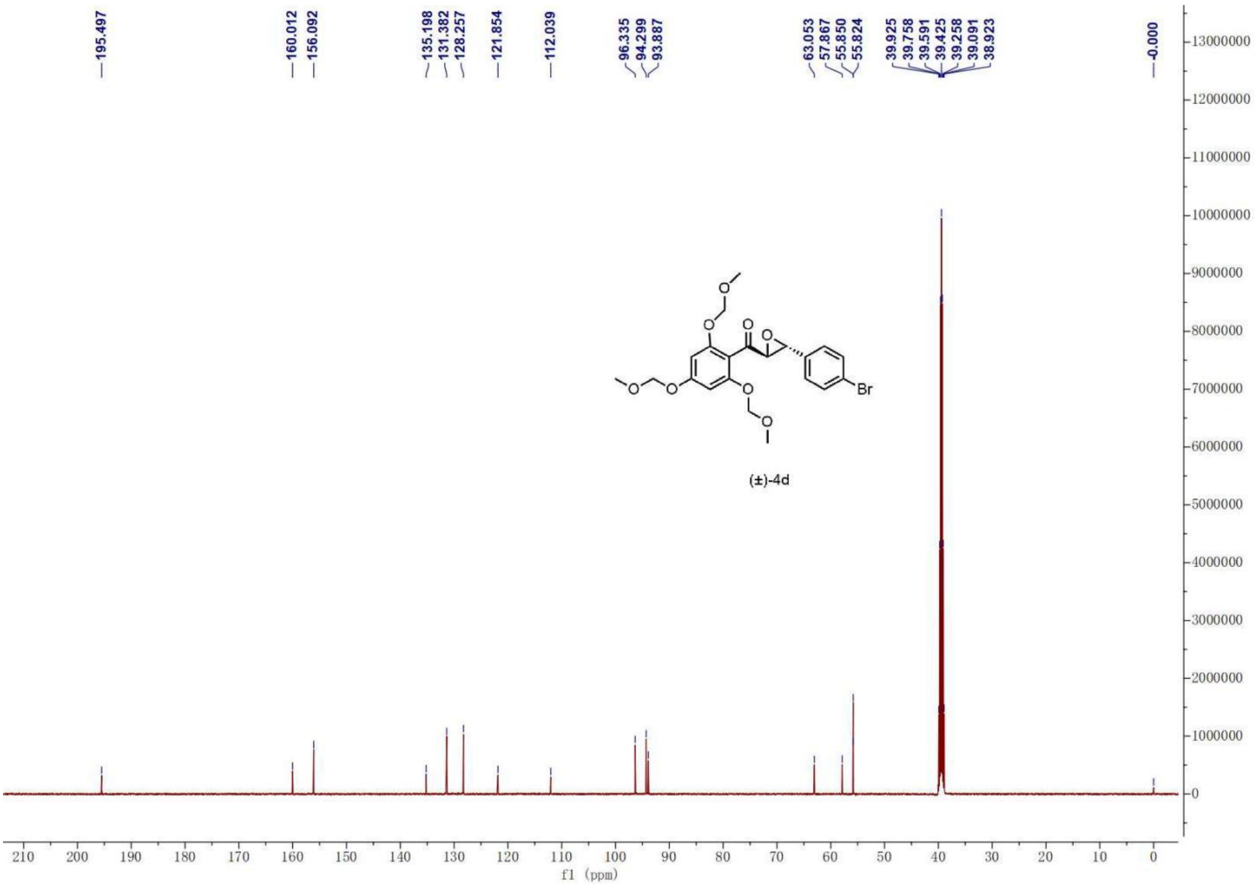

HRMS spectrum of compound (±)-**4d**

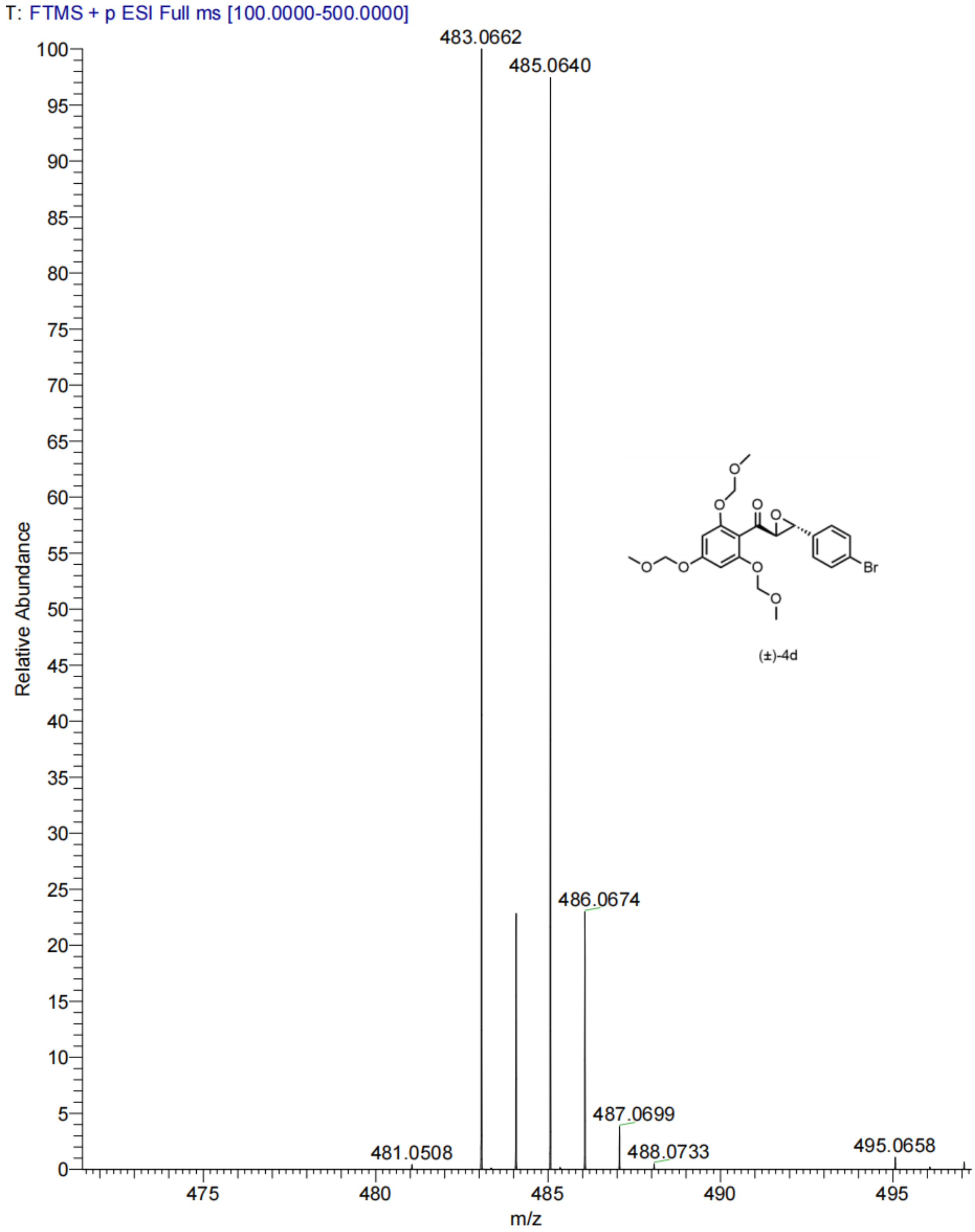

^{1}H NMR spectrum of compound (±)-**4e**

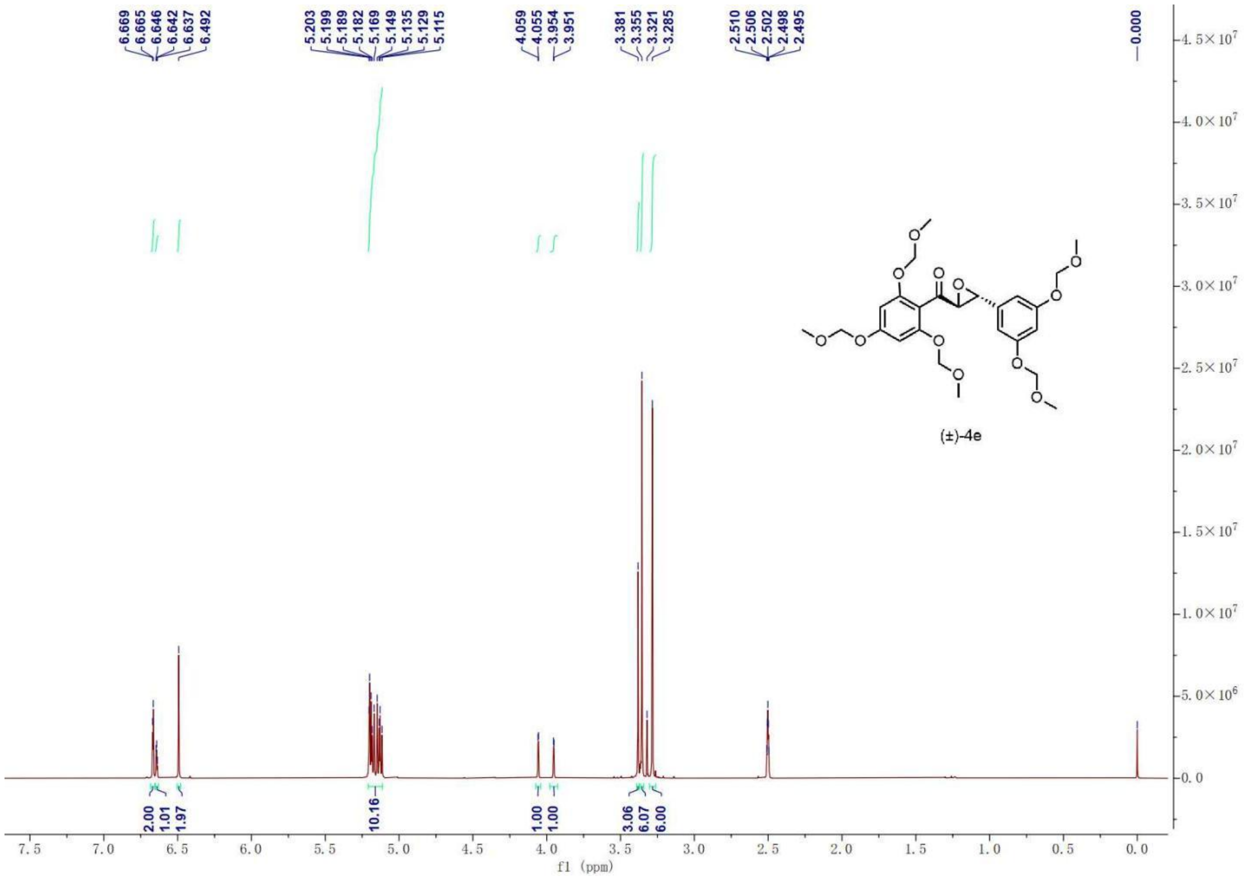

^{13}C NMR spectrum of compound (±)-**4e**

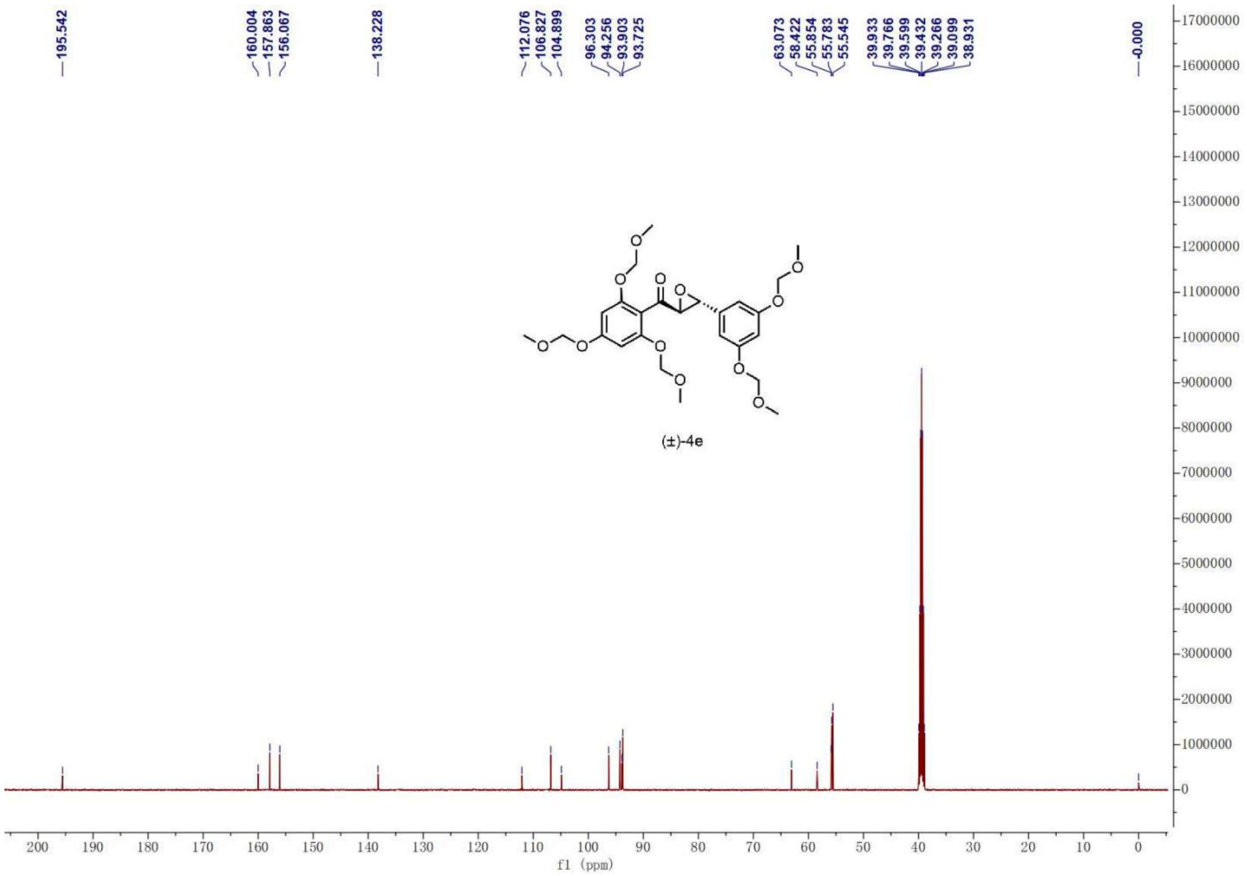

HRMS spectrum of compound (±)-**4e**

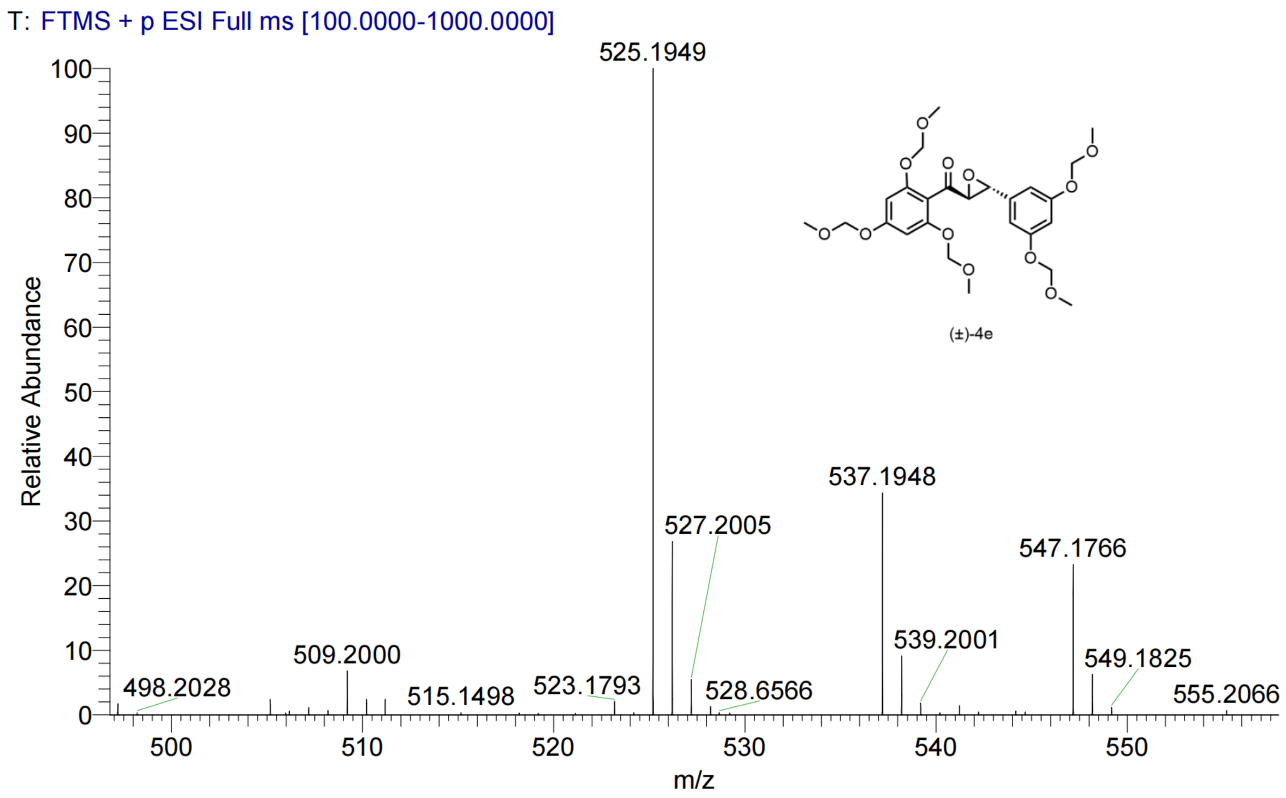

^{1}H NMR spectrum of compound (±)-**4f**

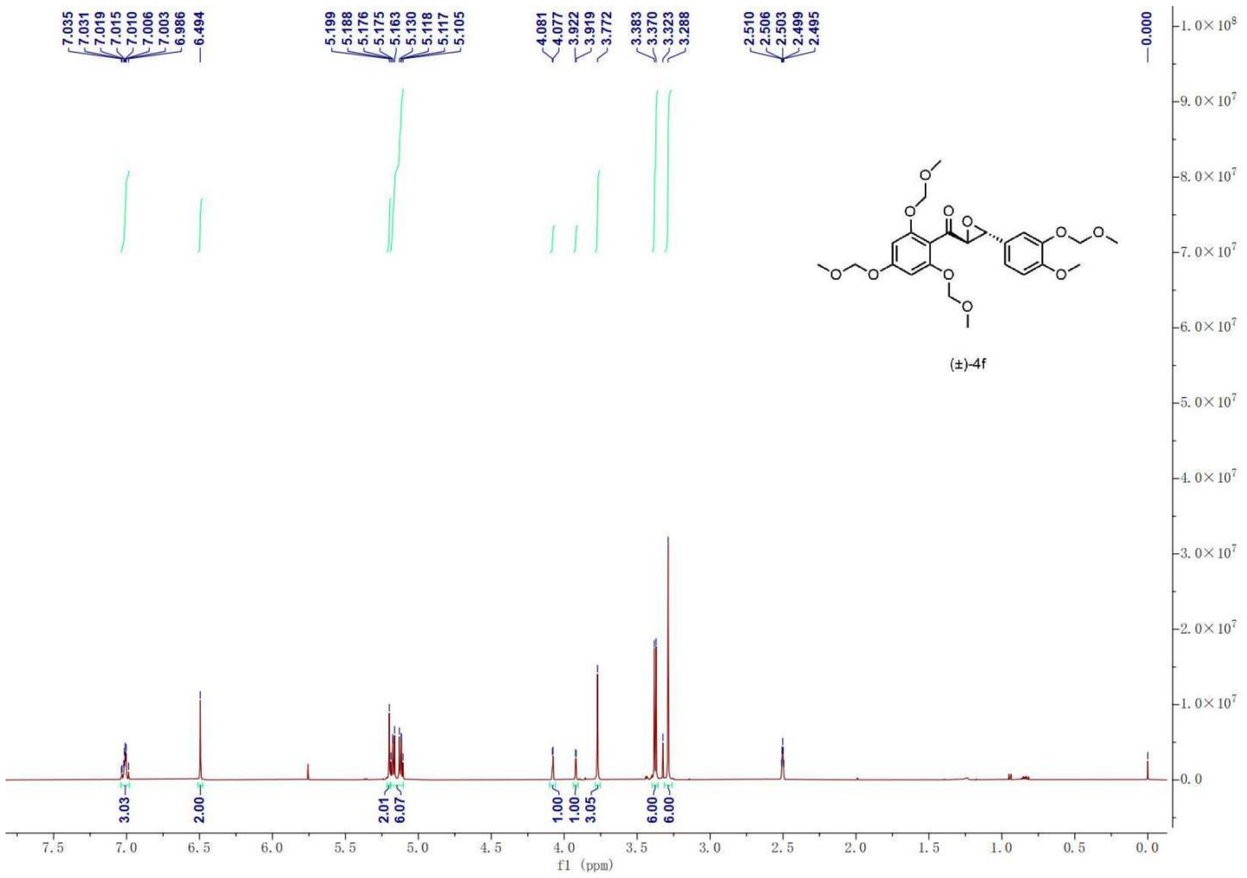

^{13}C NMR spectrum of compound (±)-**4f**

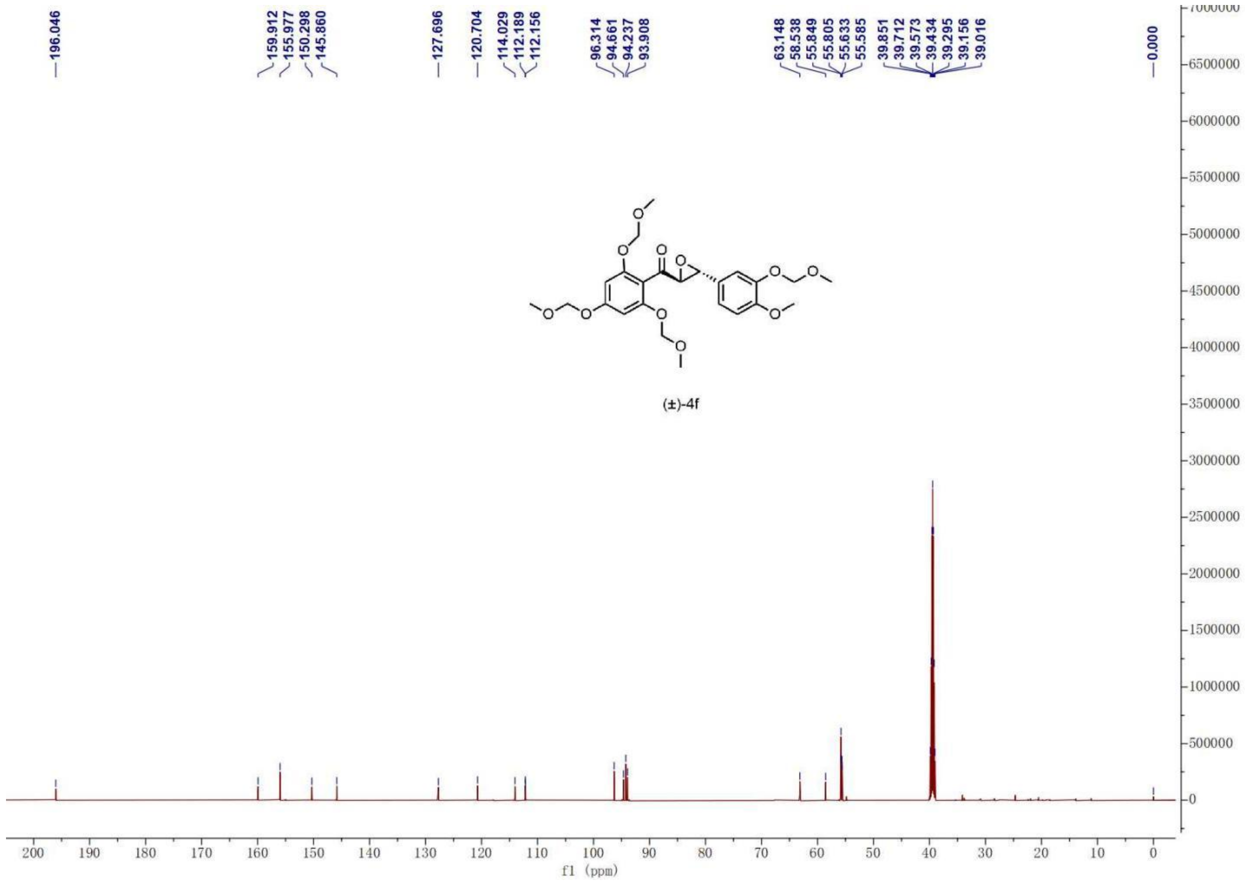

HRMS spectrum of compound (±)-**4f**

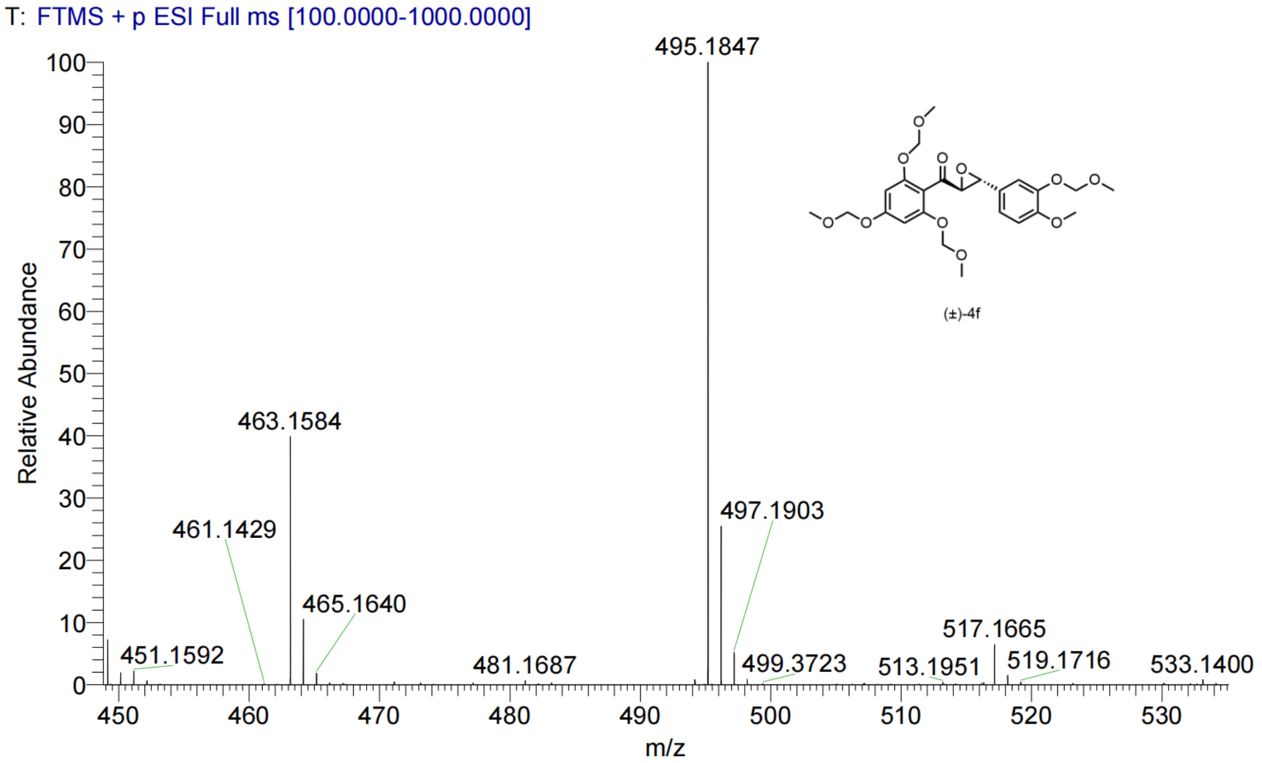

^{1}H NMR spectrum of compound (±)-**4g**

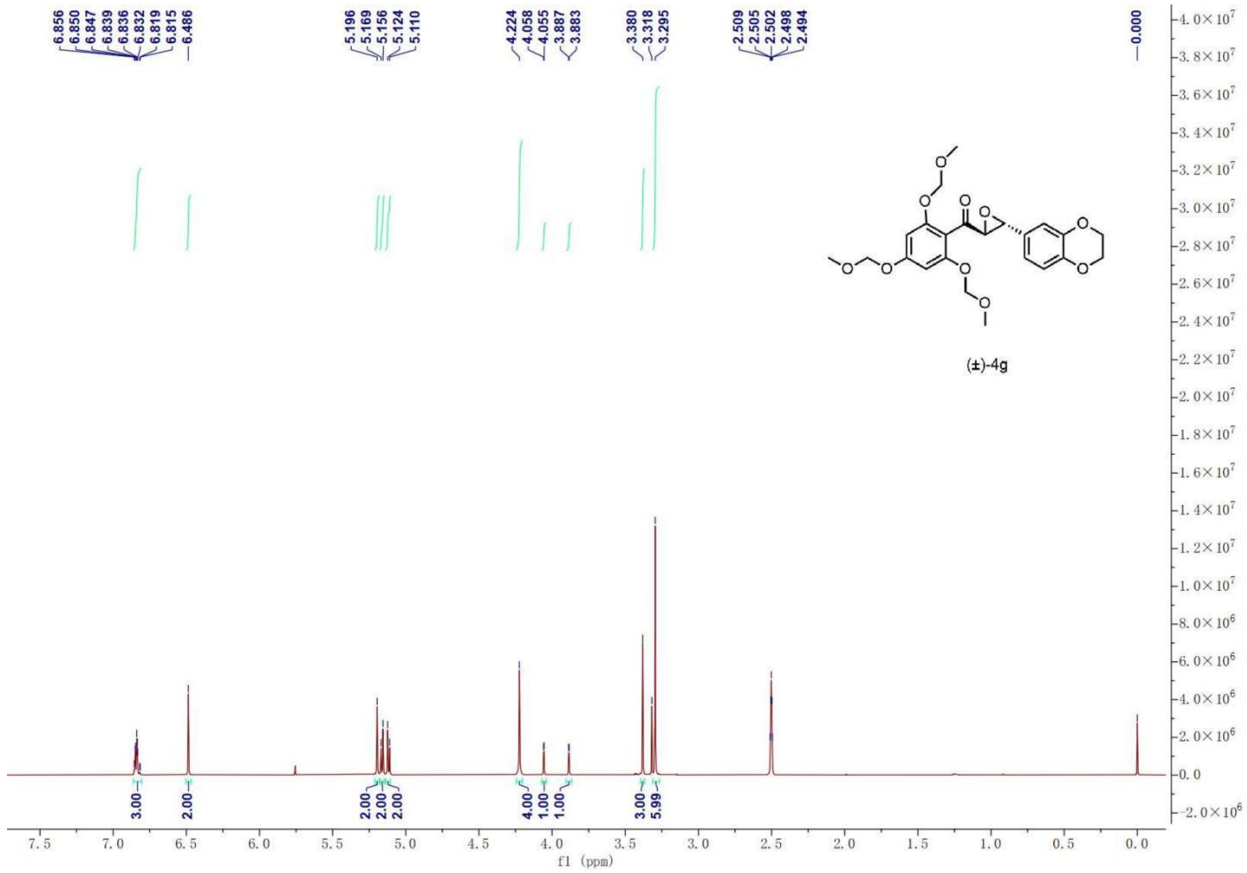

^{13}C NMR spectrum of compound (±)-**4g**

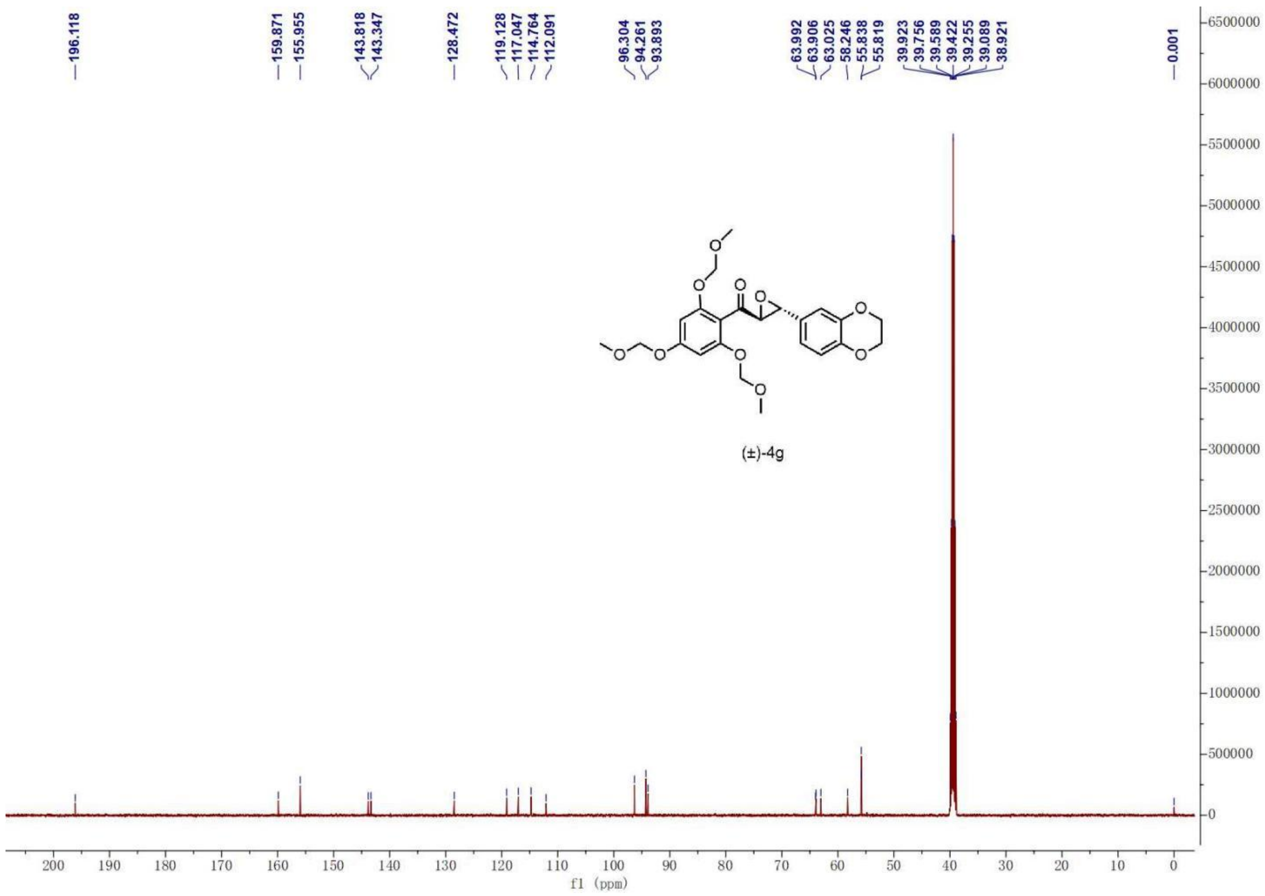

HRMS spectrum of compound (±)-**4g**

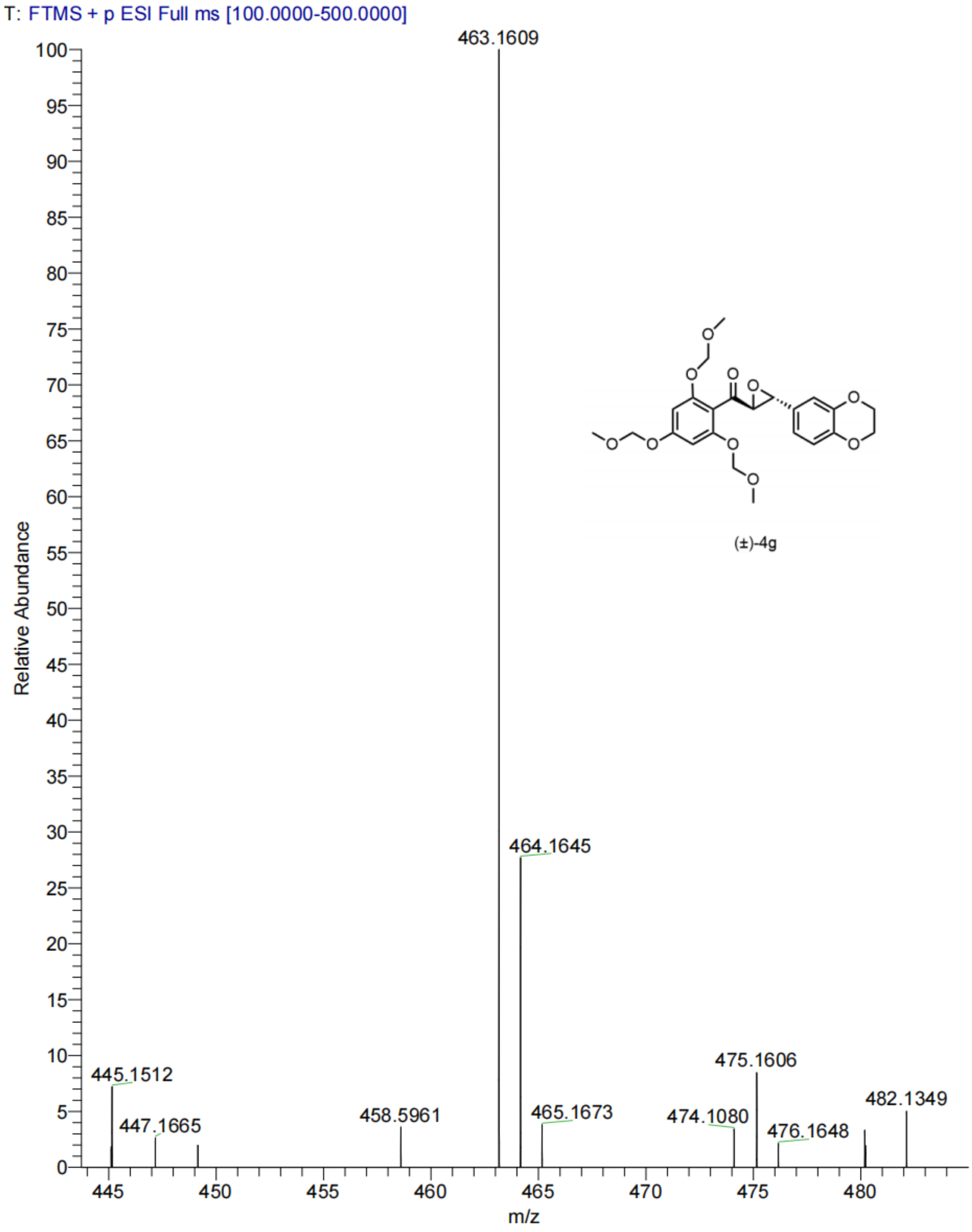

^{1}H NMR spectrum of compound (±)-**4h**

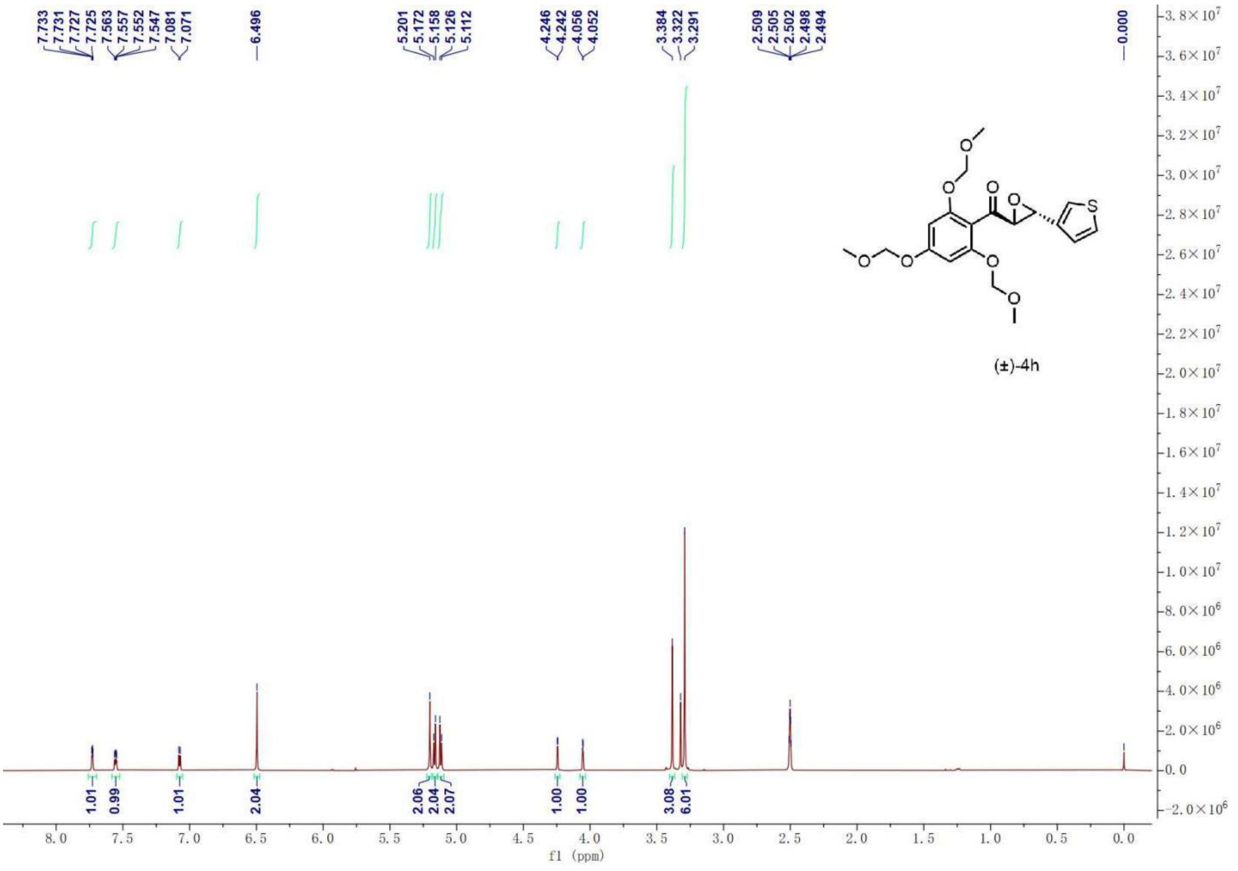

^{13}C NMR spectrum of compound (±)-**4h**

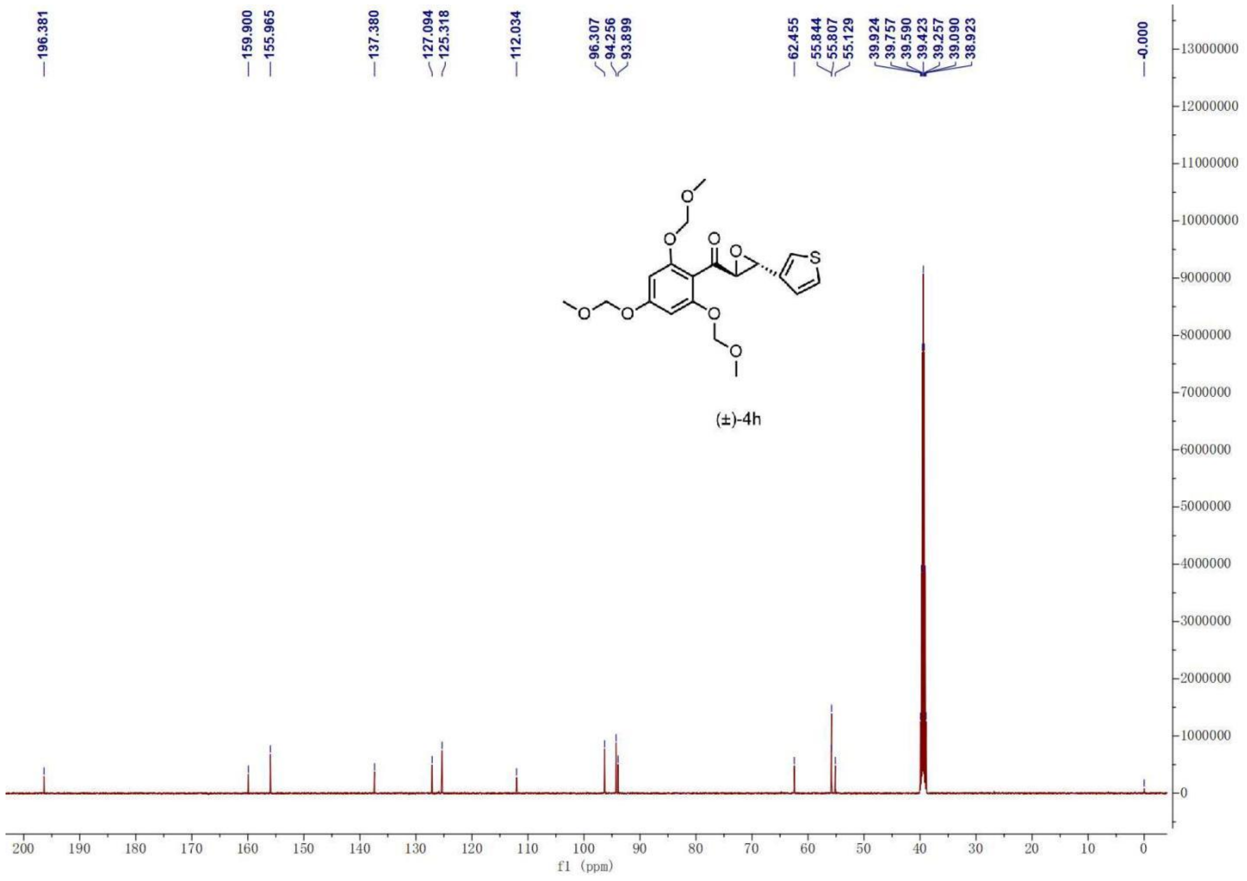

HRMS spectrum of compound (±)-**4h**

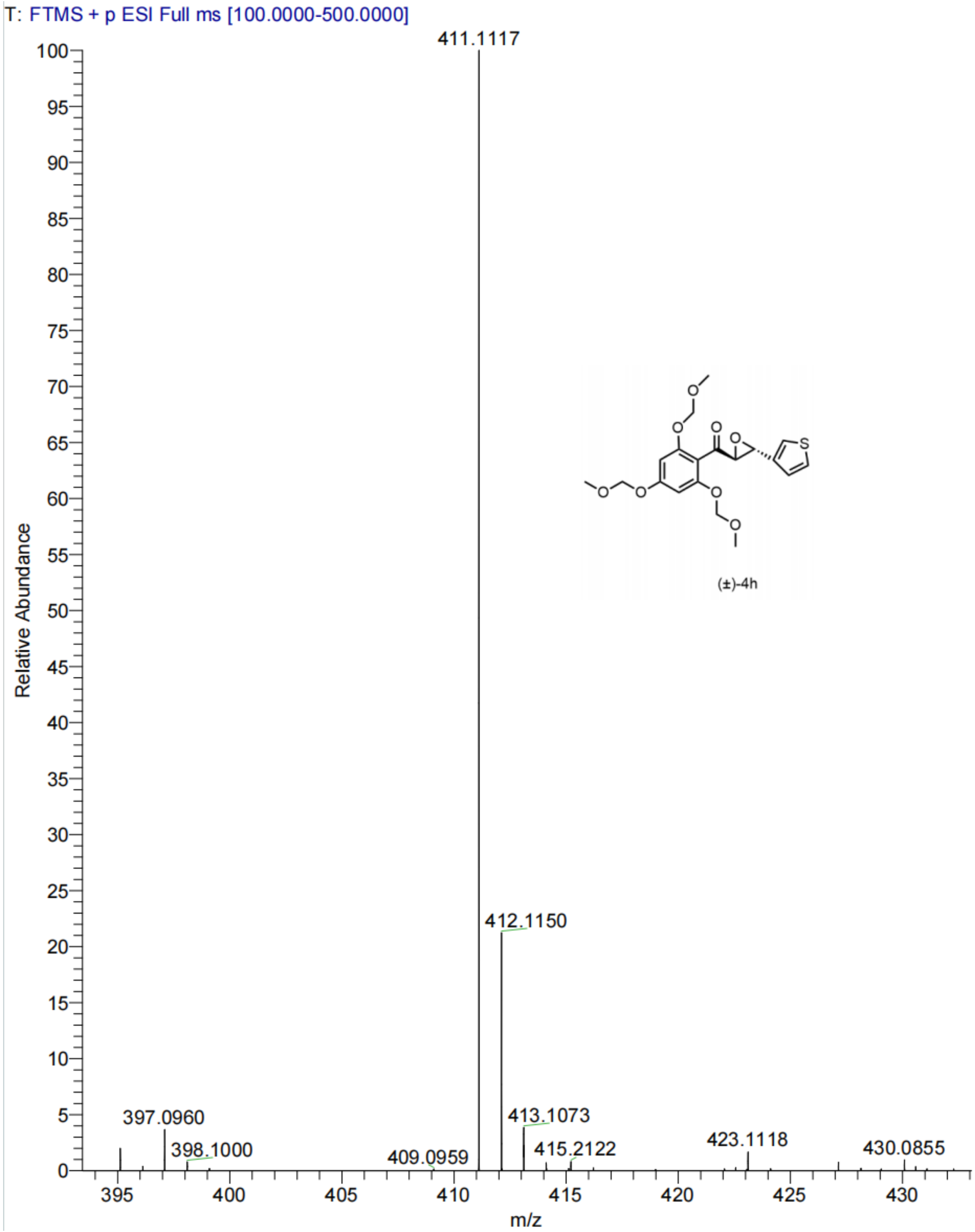

^1H NMR spectrum of compound (±)-**4i**

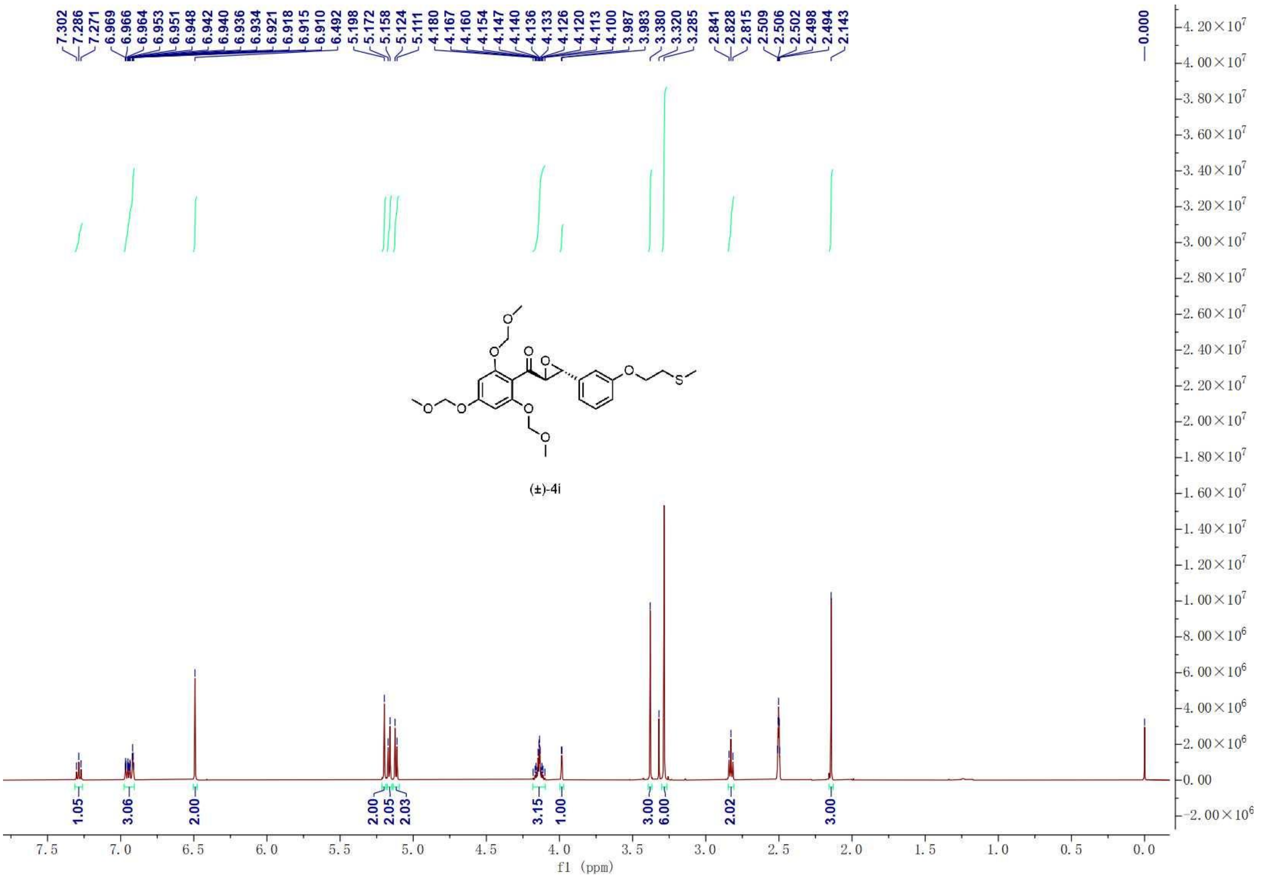

^{13}C NMR spectrum of compound (±)-**4i**

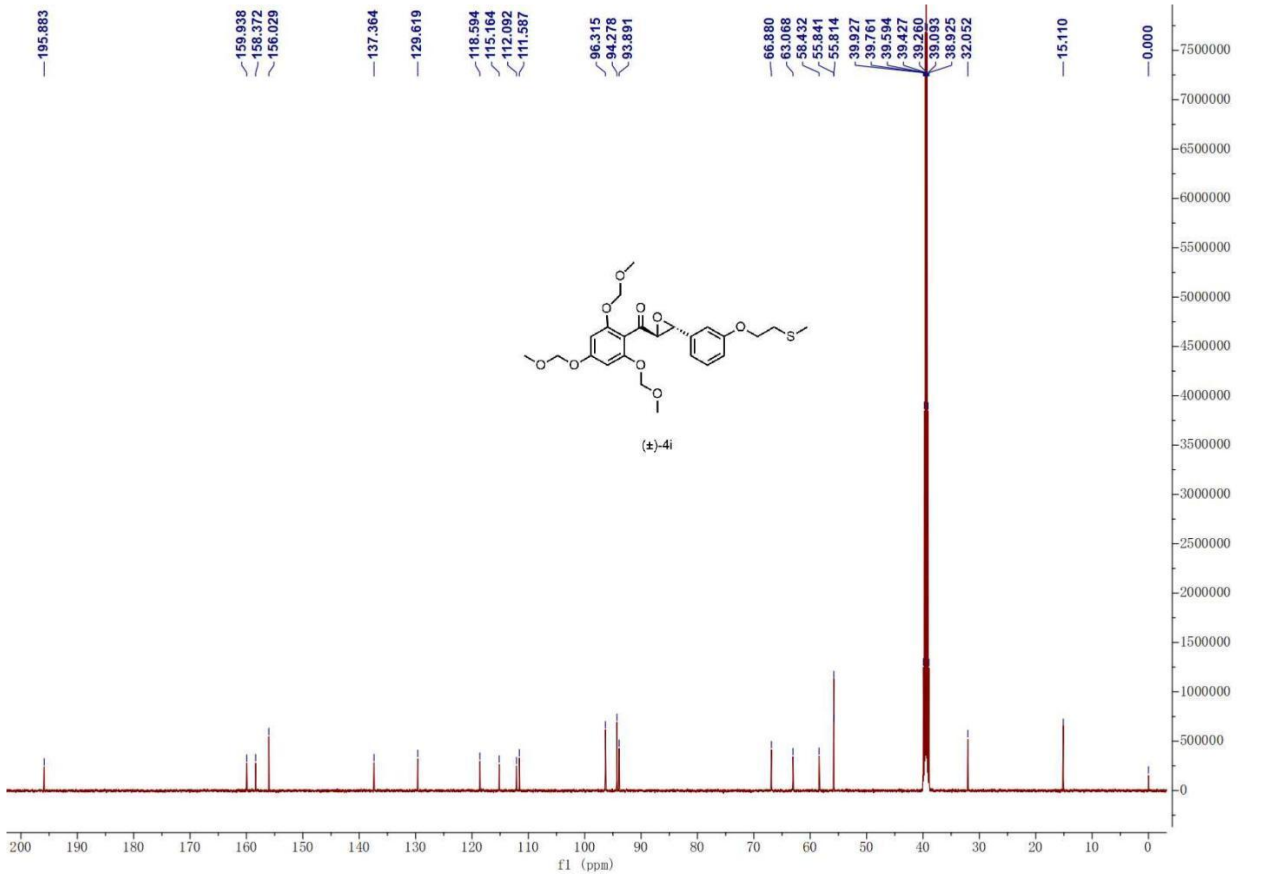

HRMS spectrum of compound (±)-**4i**

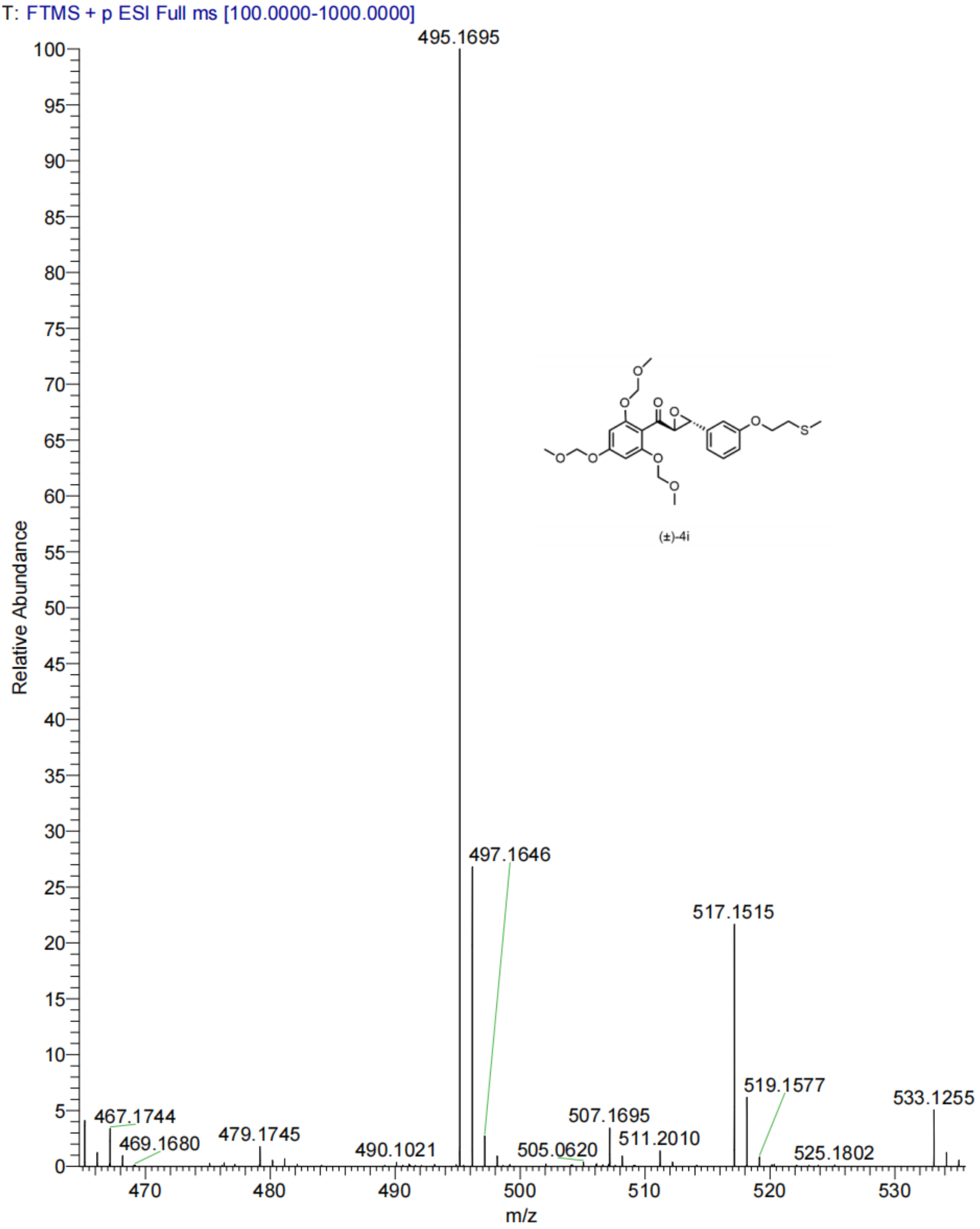

^{1}H NMR spectrum of compound (±)-**4j**

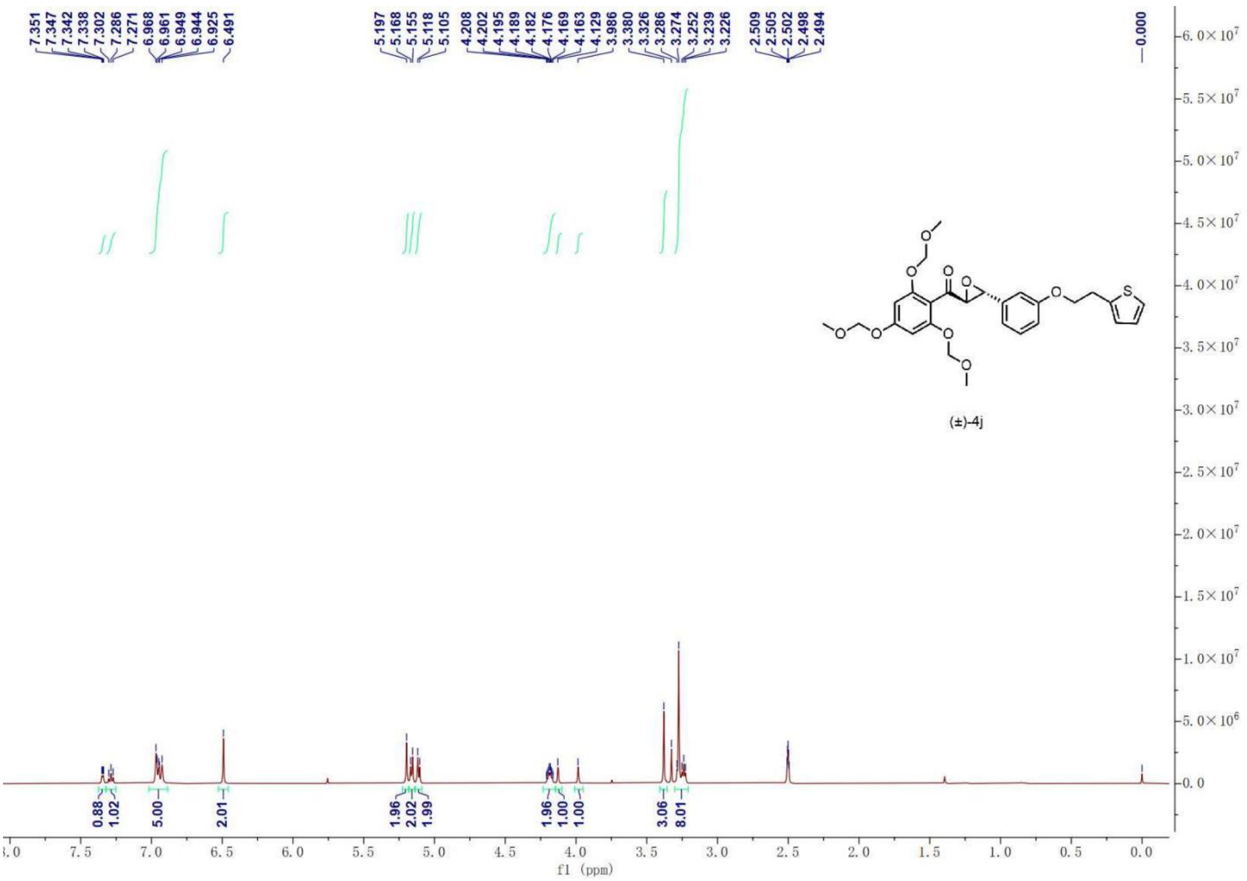

^{13}C NMR spectrum of compound (±)-**4j**

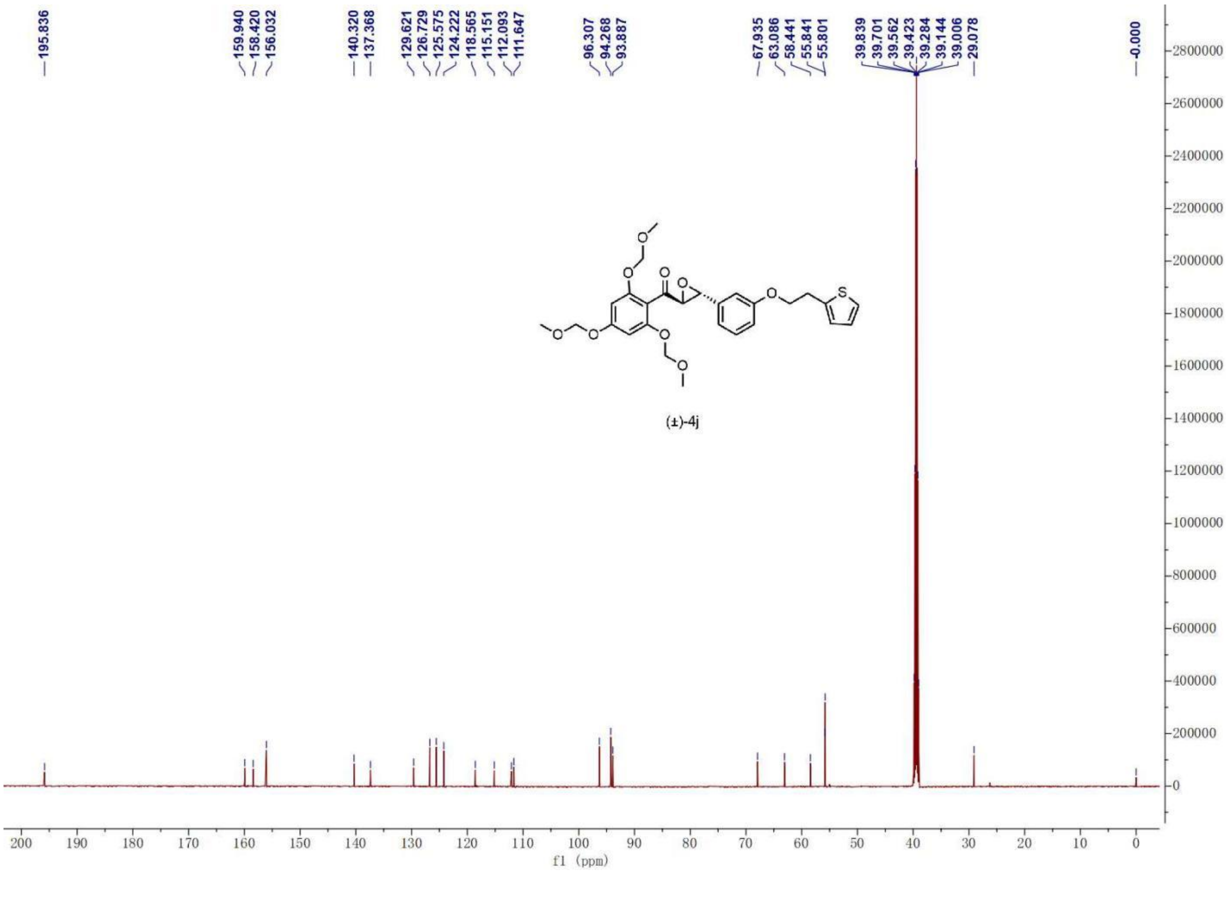

HRMS spectrum of compound (±)-**4j**

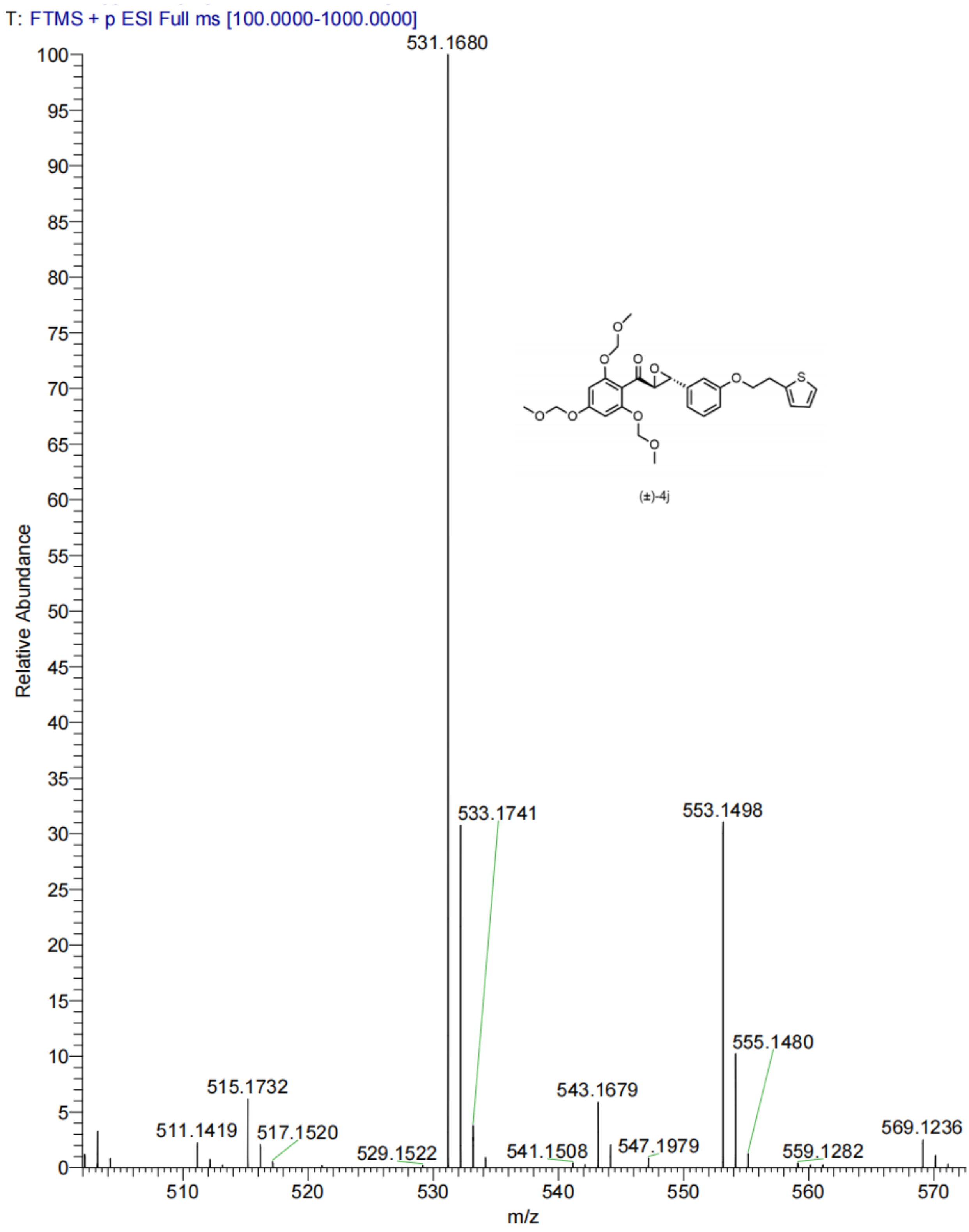

^{1}H NMR spectrum of compound (±)-**4k**

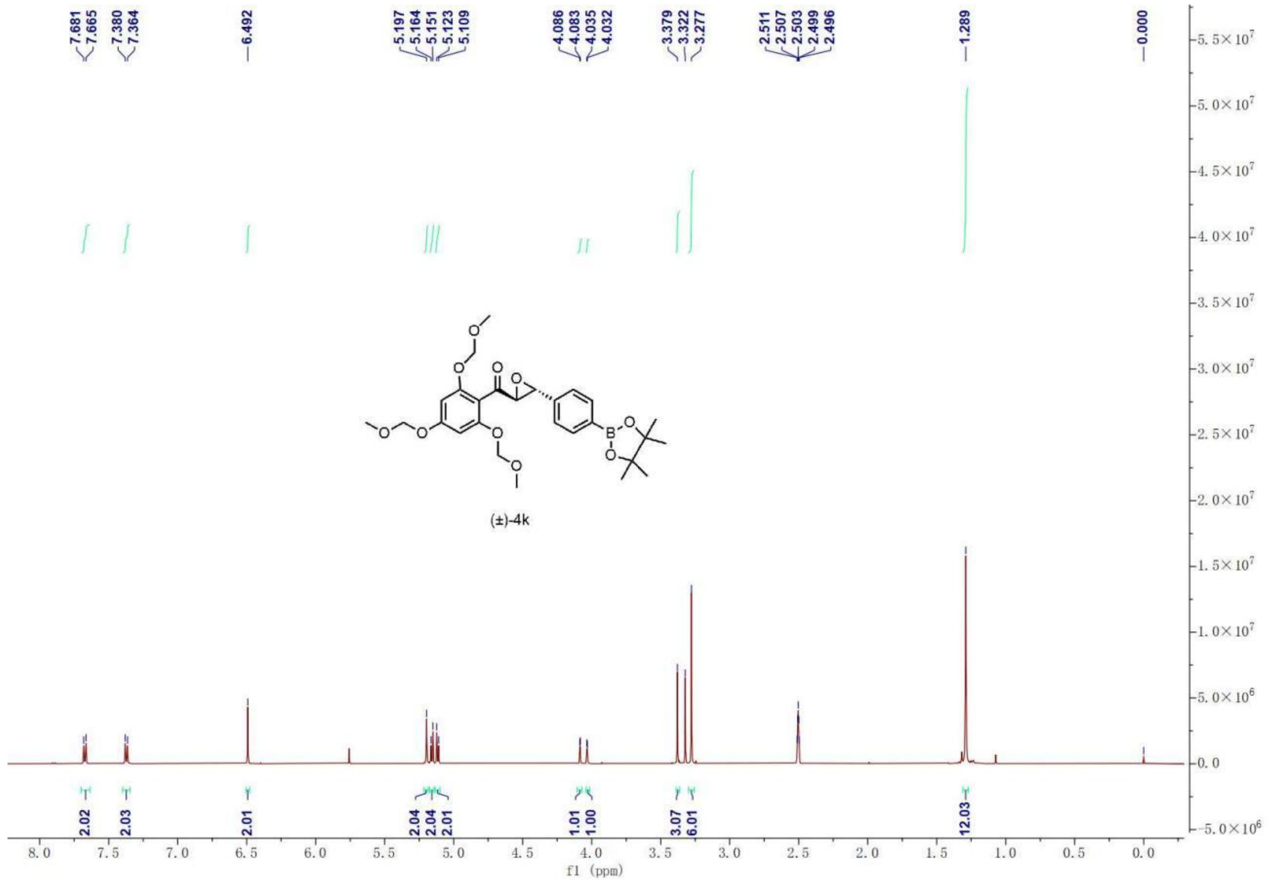

^{13}C NMR spectrum of compound (±)-**4k**

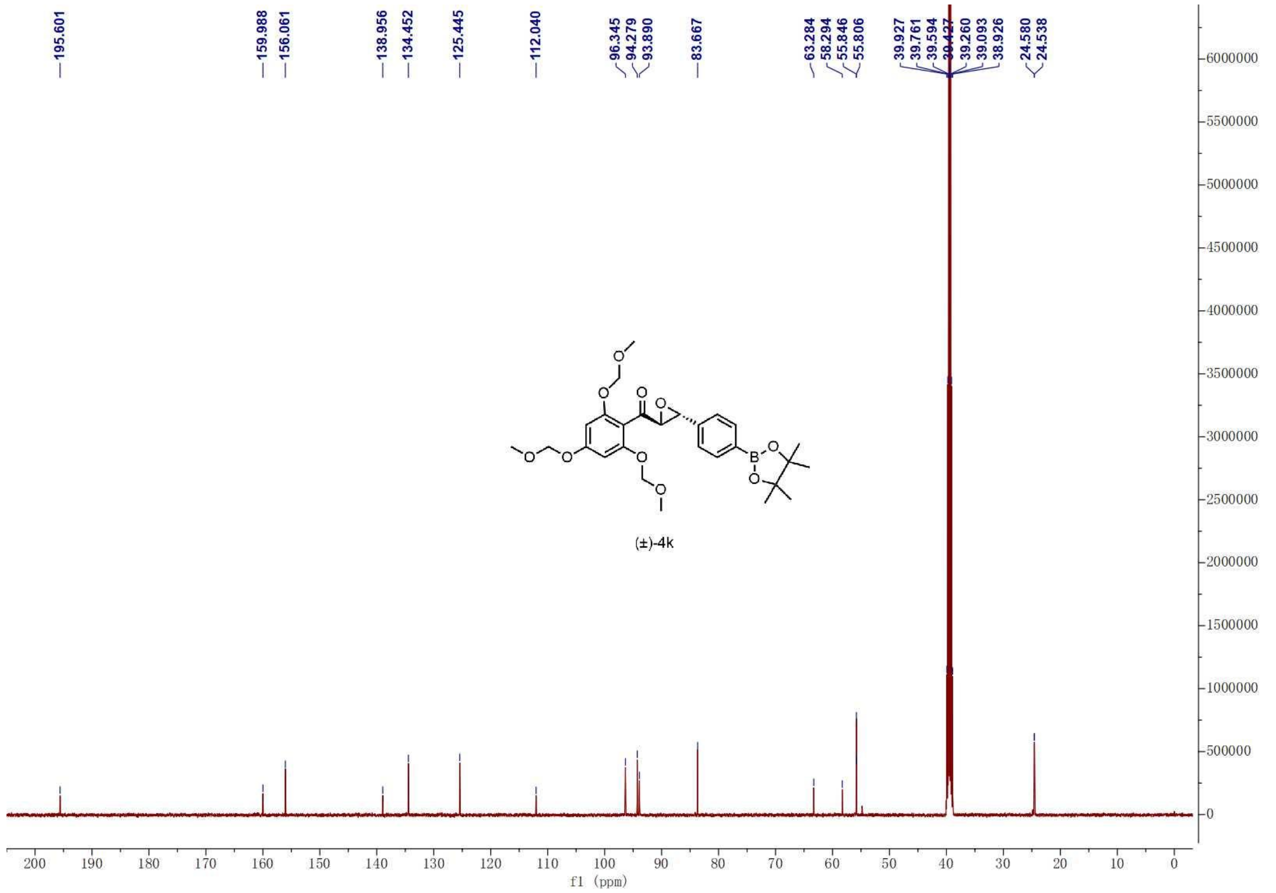

HRMS spectrum of compound (±)-**4k**

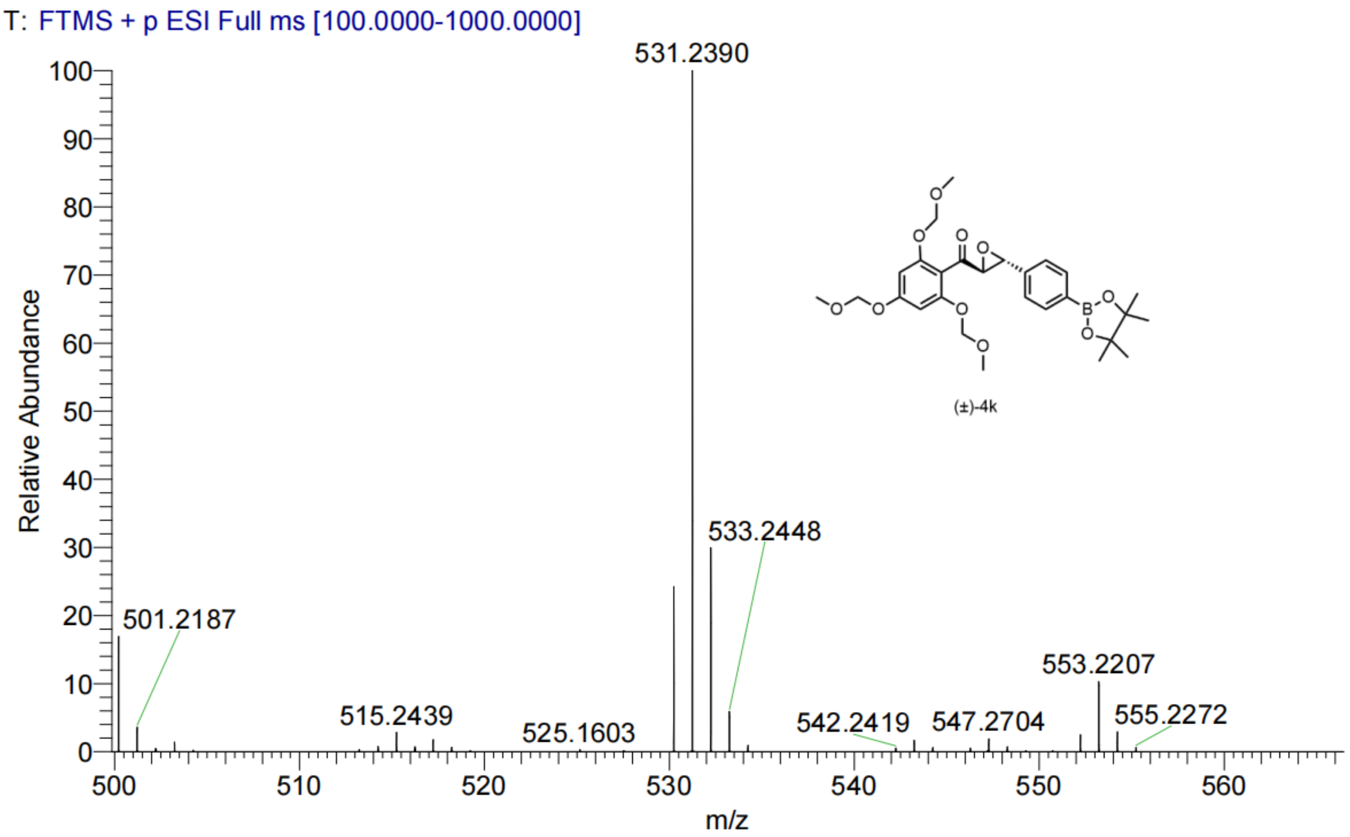

^{1}H NMR spectrum of compound (±)-**4l**

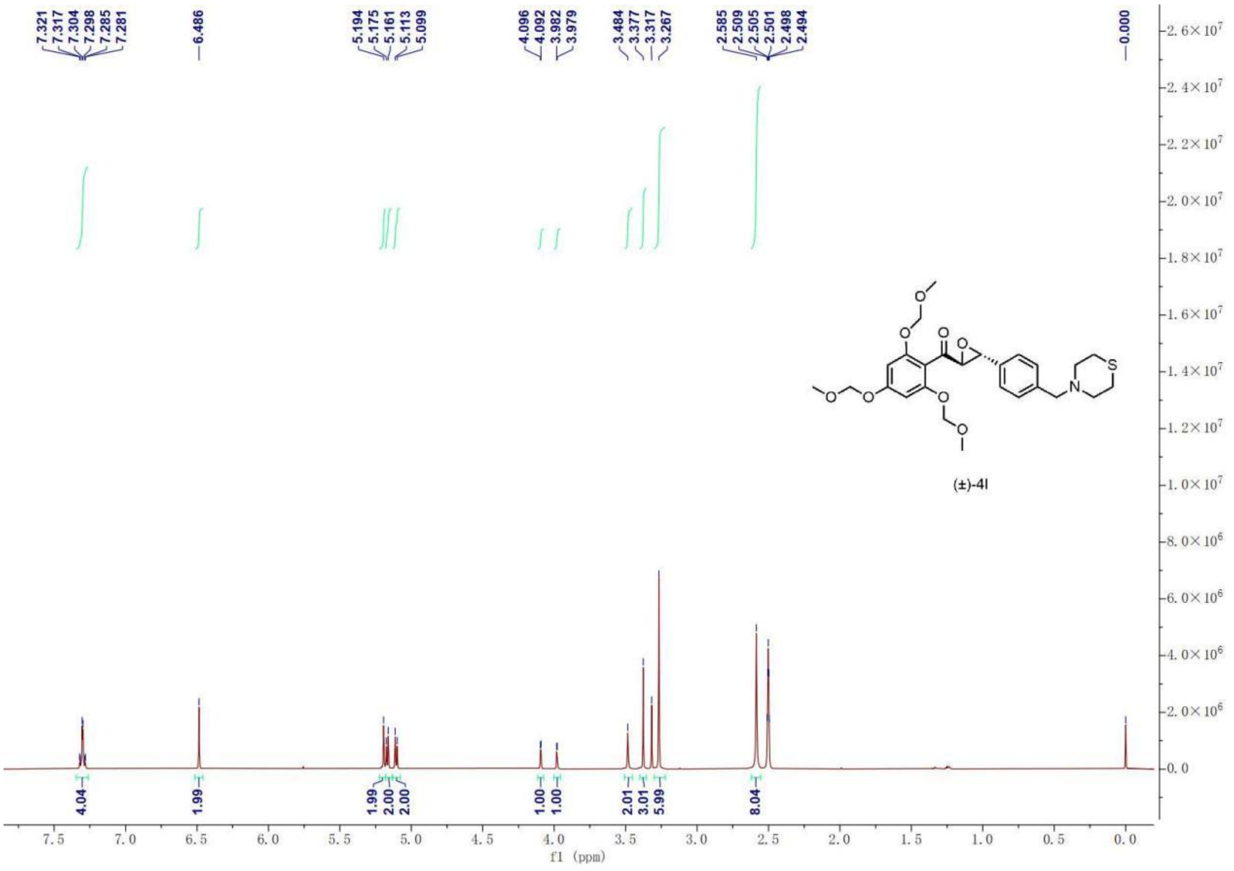

^{13}C NMR spectrum of compound (±)-**4l**

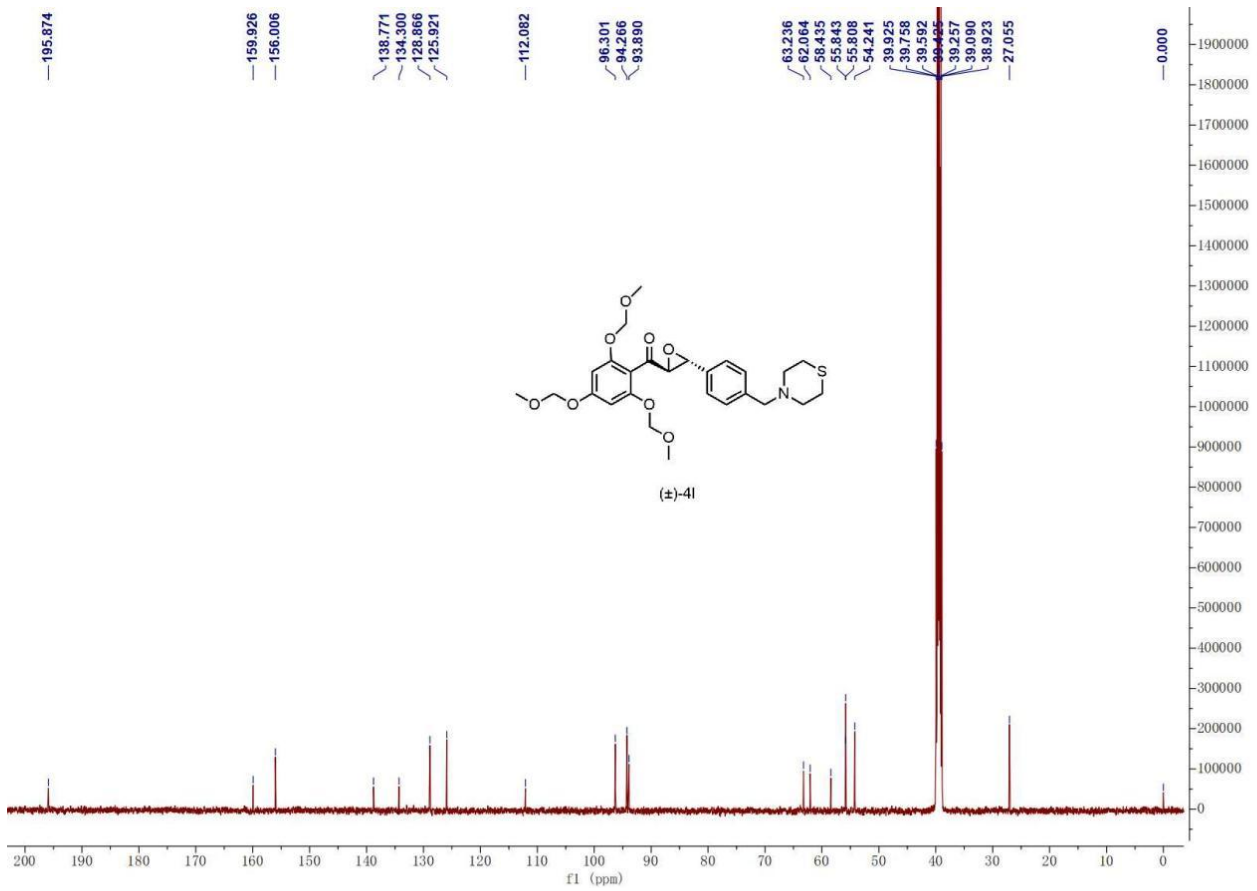

HRMS spectrum of compound (±)-**4l**

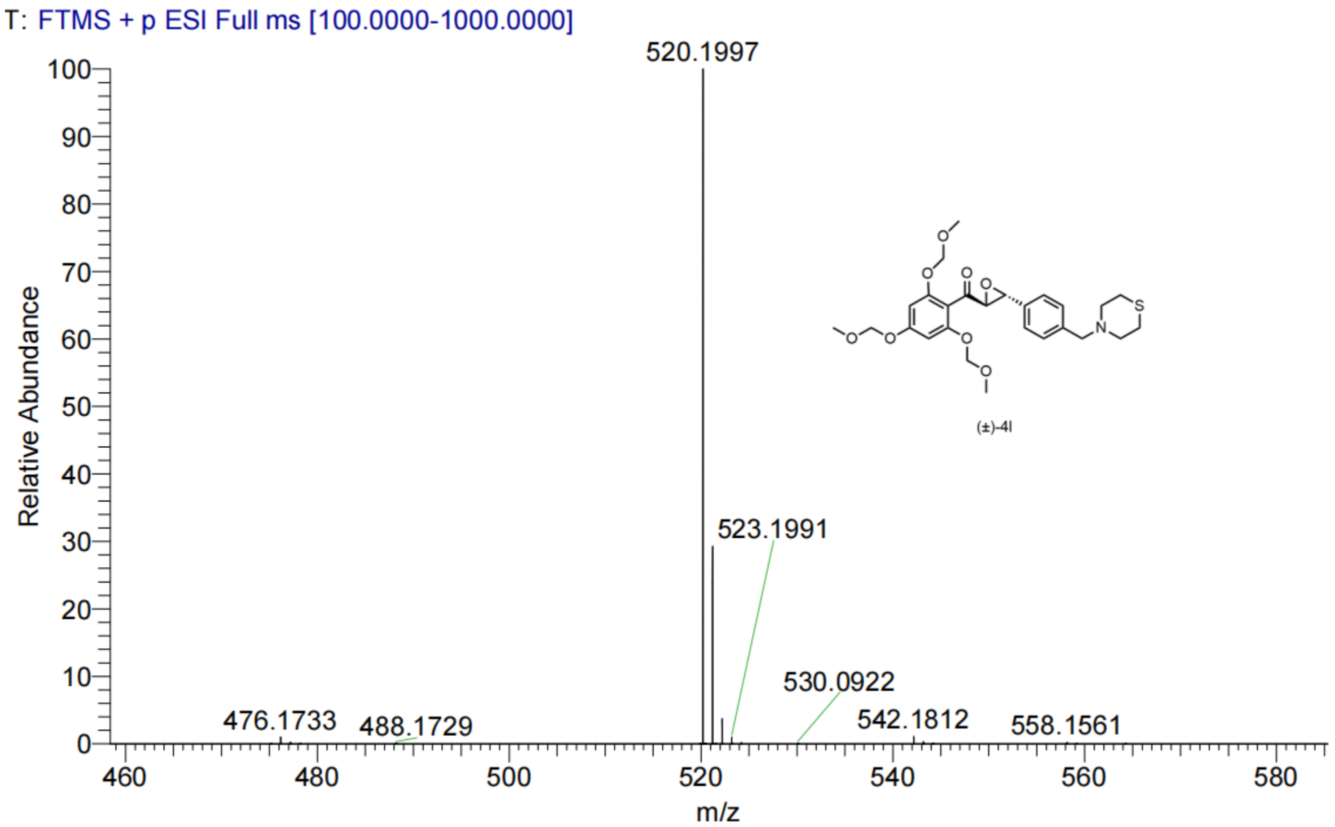

^{1}H NMR spectrum of compound *trans*-(±)-taxifolin

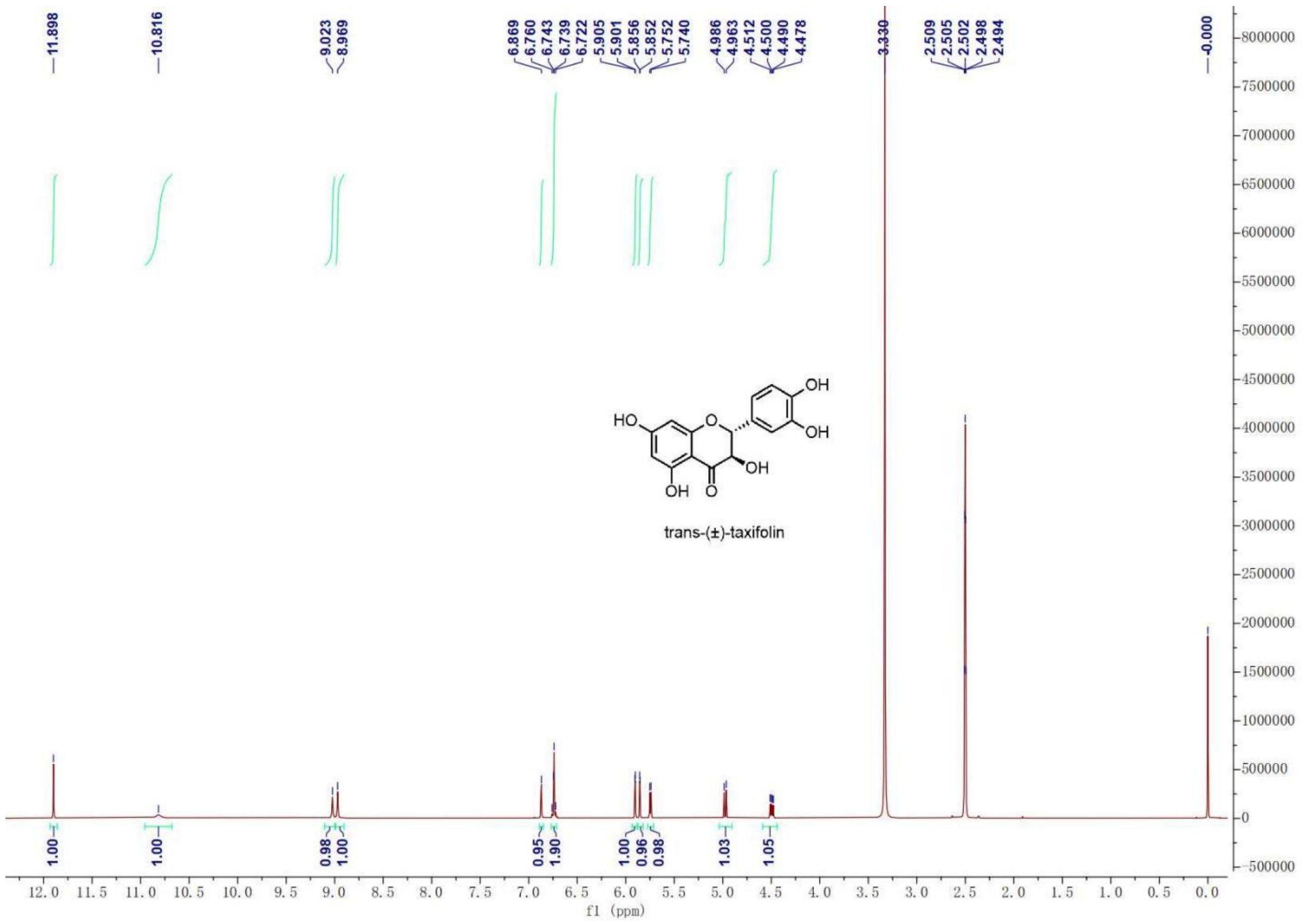

^{1}H NMR spectrum of compound (±)-**TD-1**

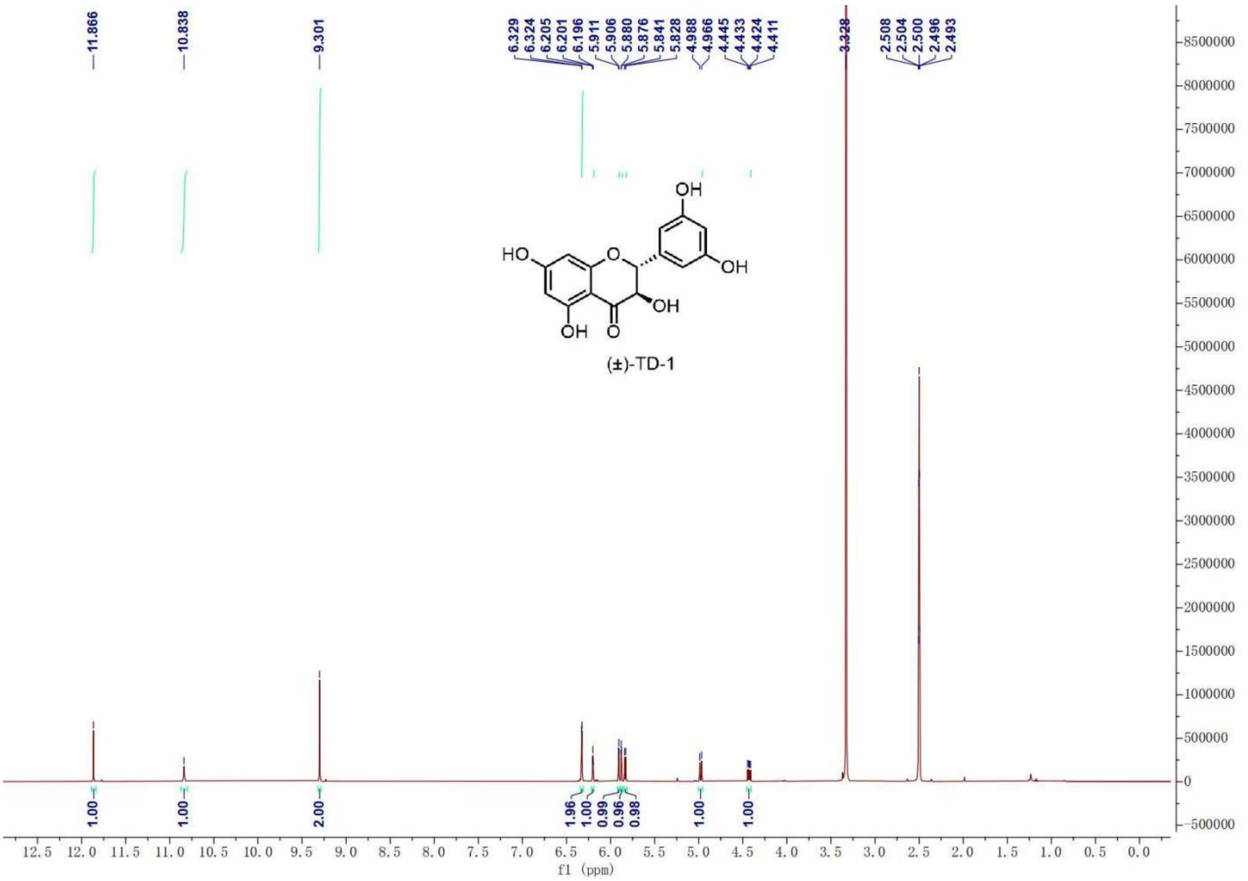

^{1}H NMR spectrum of compound (±)-**TD-2**

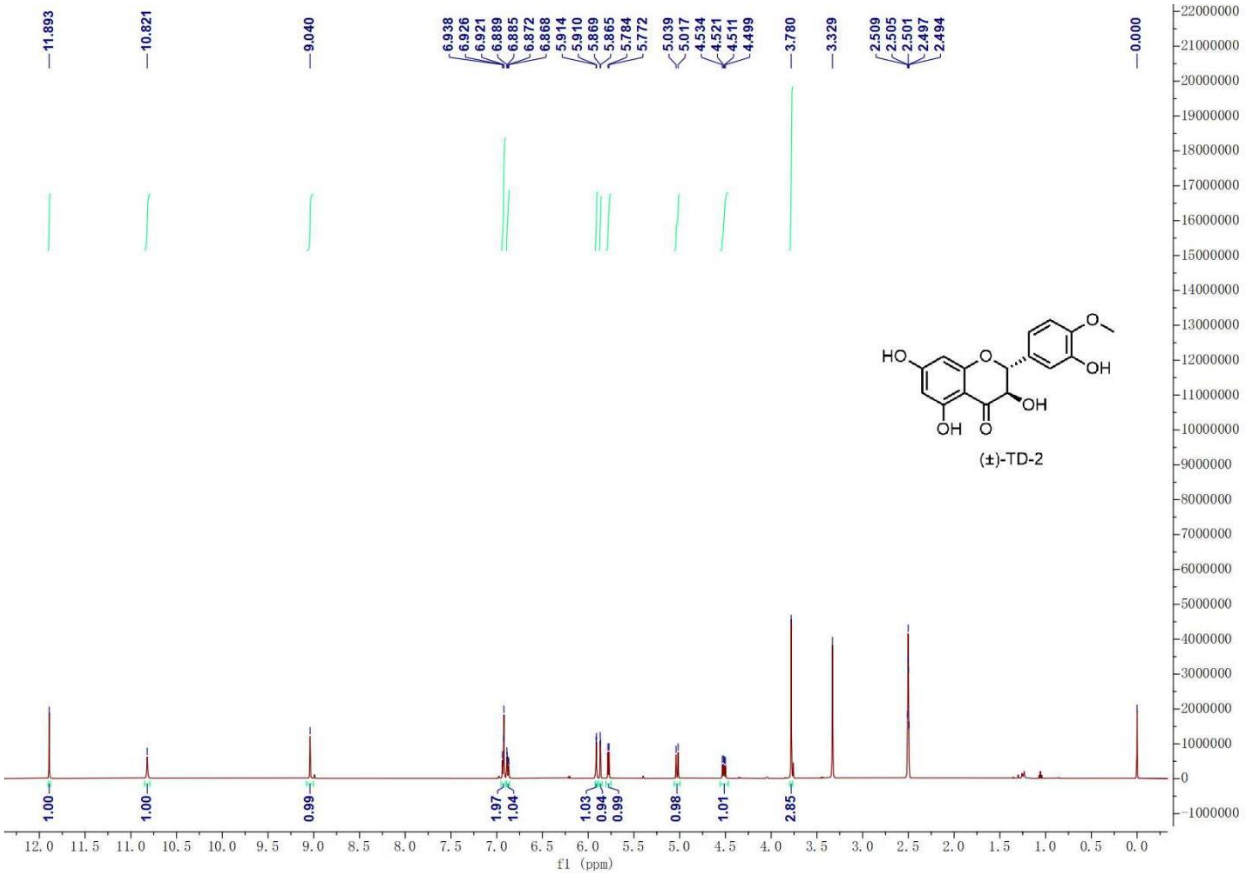

^{1}H NMR spectrum of compound (±)-**TD-3**

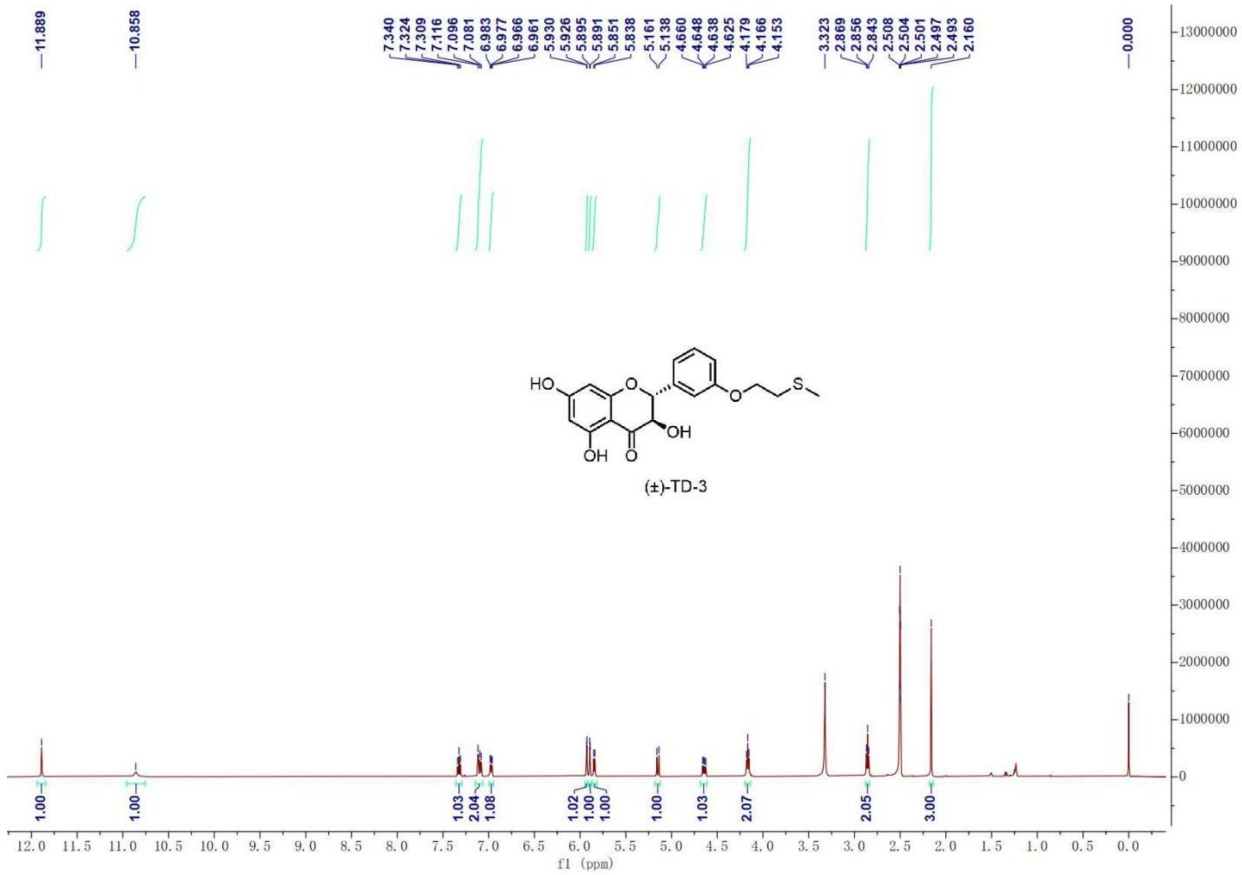

^{13}C NMR spectrum of compound (±)-**TD-3**

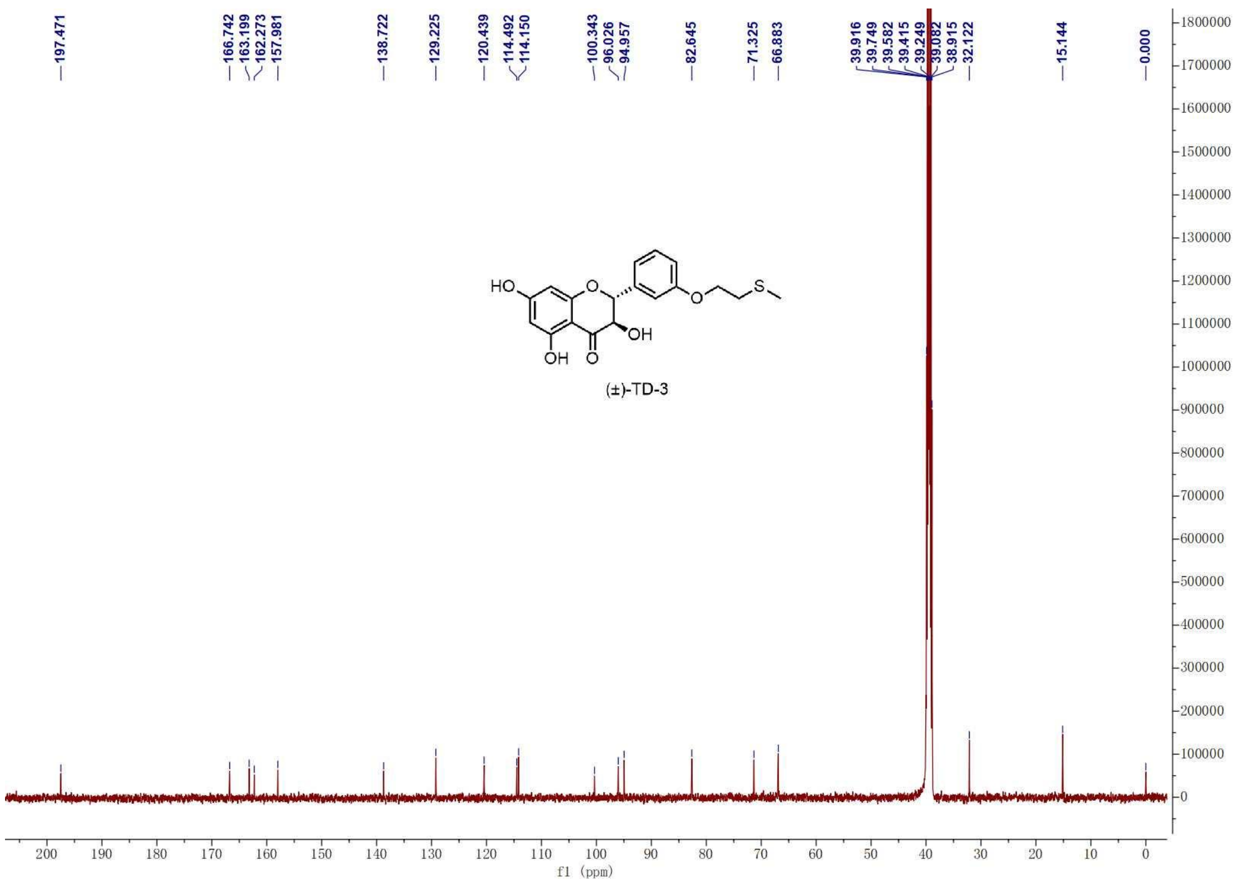

HRMS spectrum of compound (±)-**TD-3**

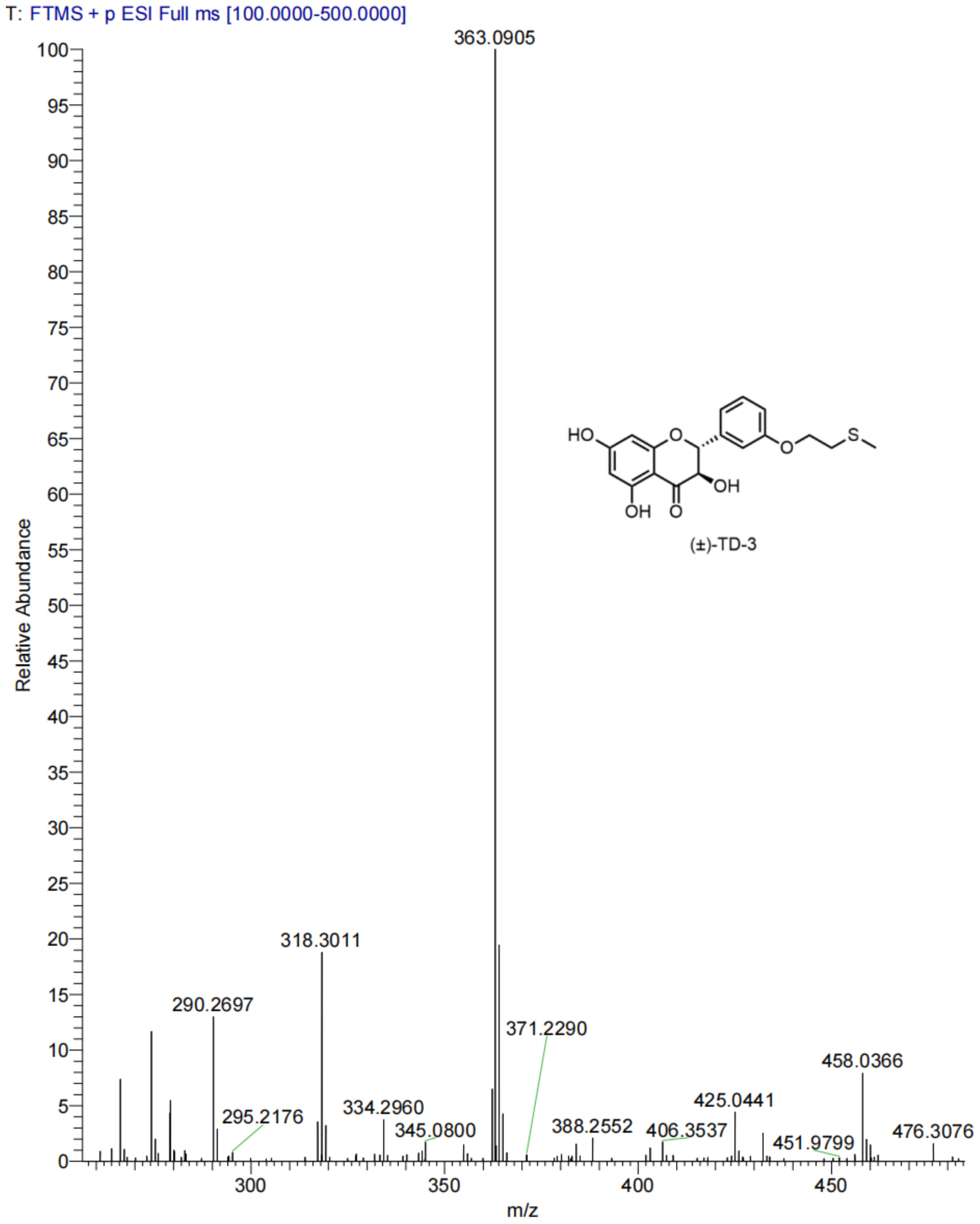

^{1}H NMR spectrum of compound (±)-**TD-4**

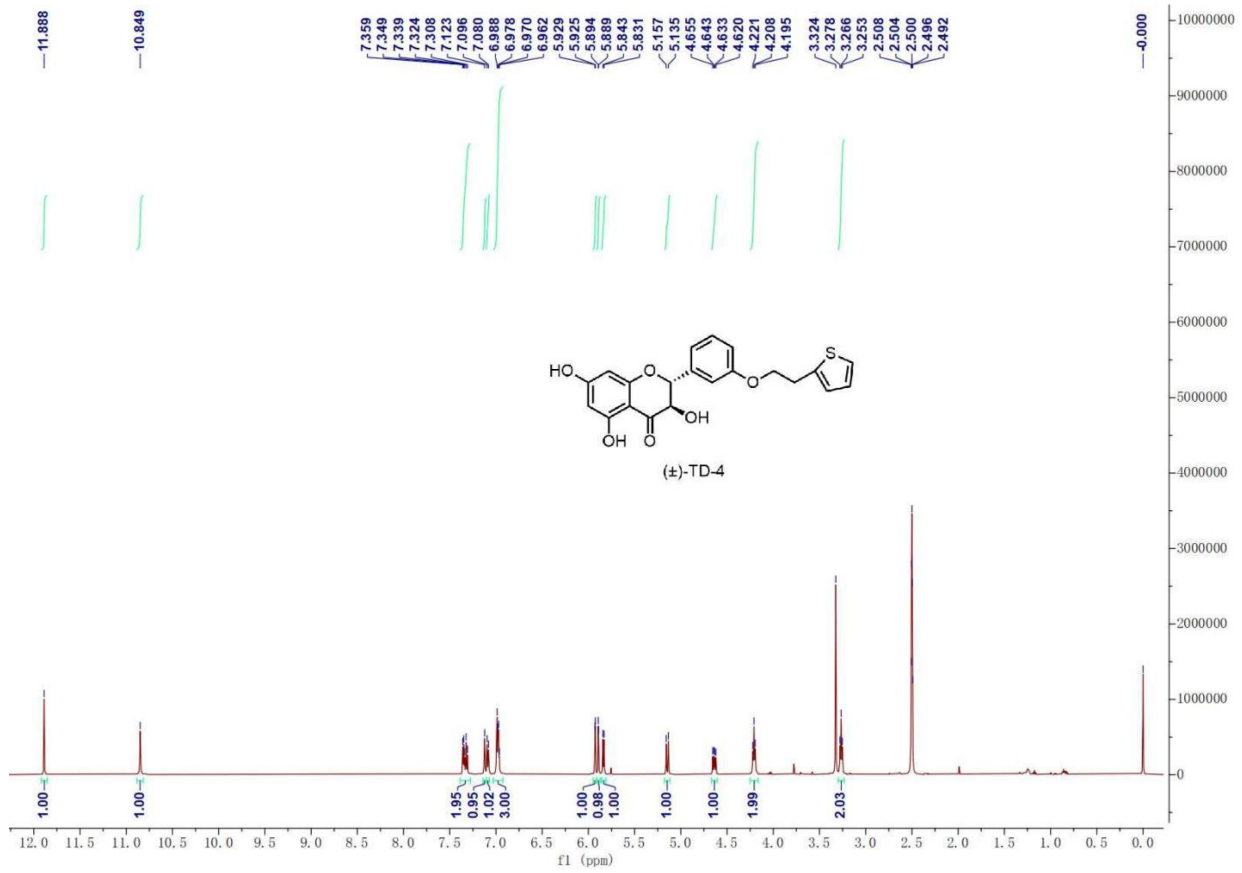

^{13}C NMR spectrum of compound (±)-**TD-4**

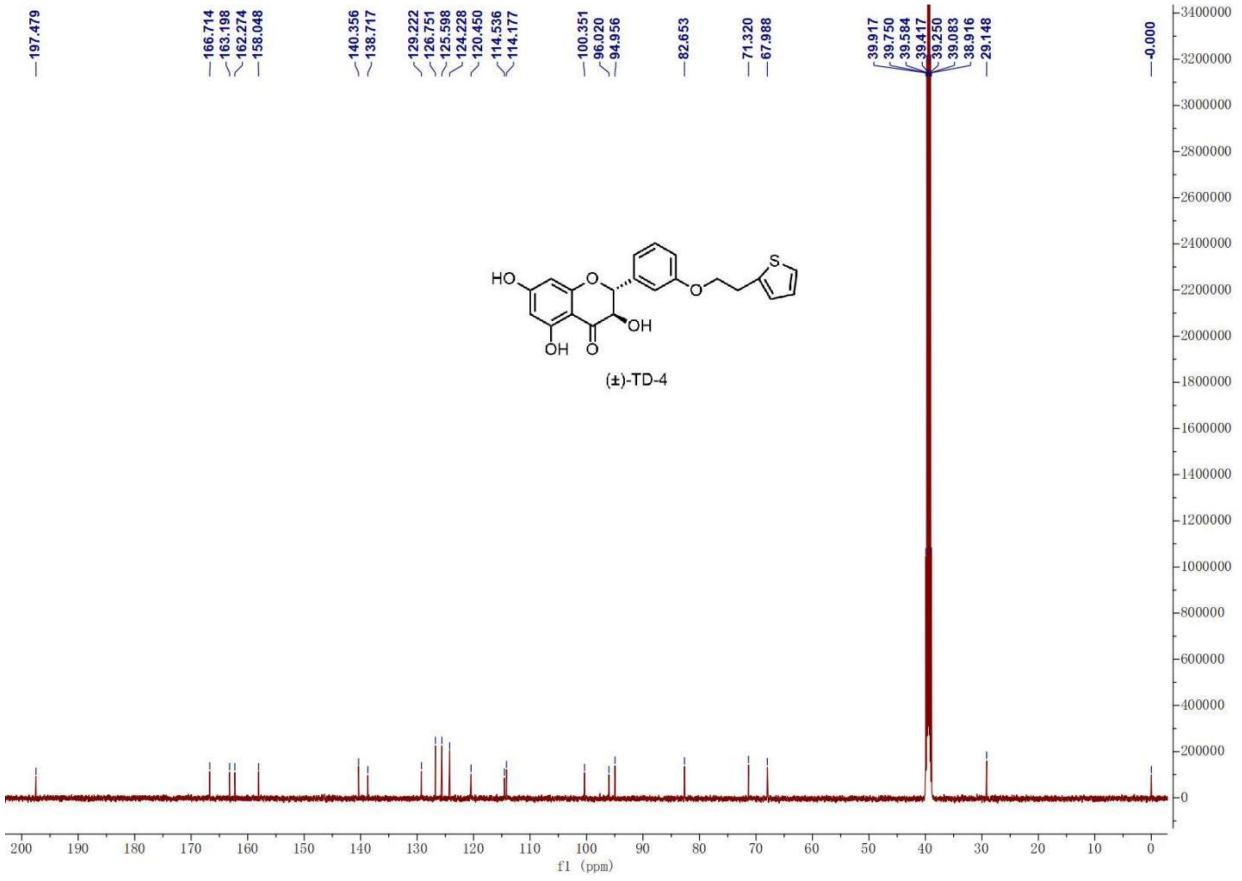

HRMS spectrum of compound (±)-**TD-4**

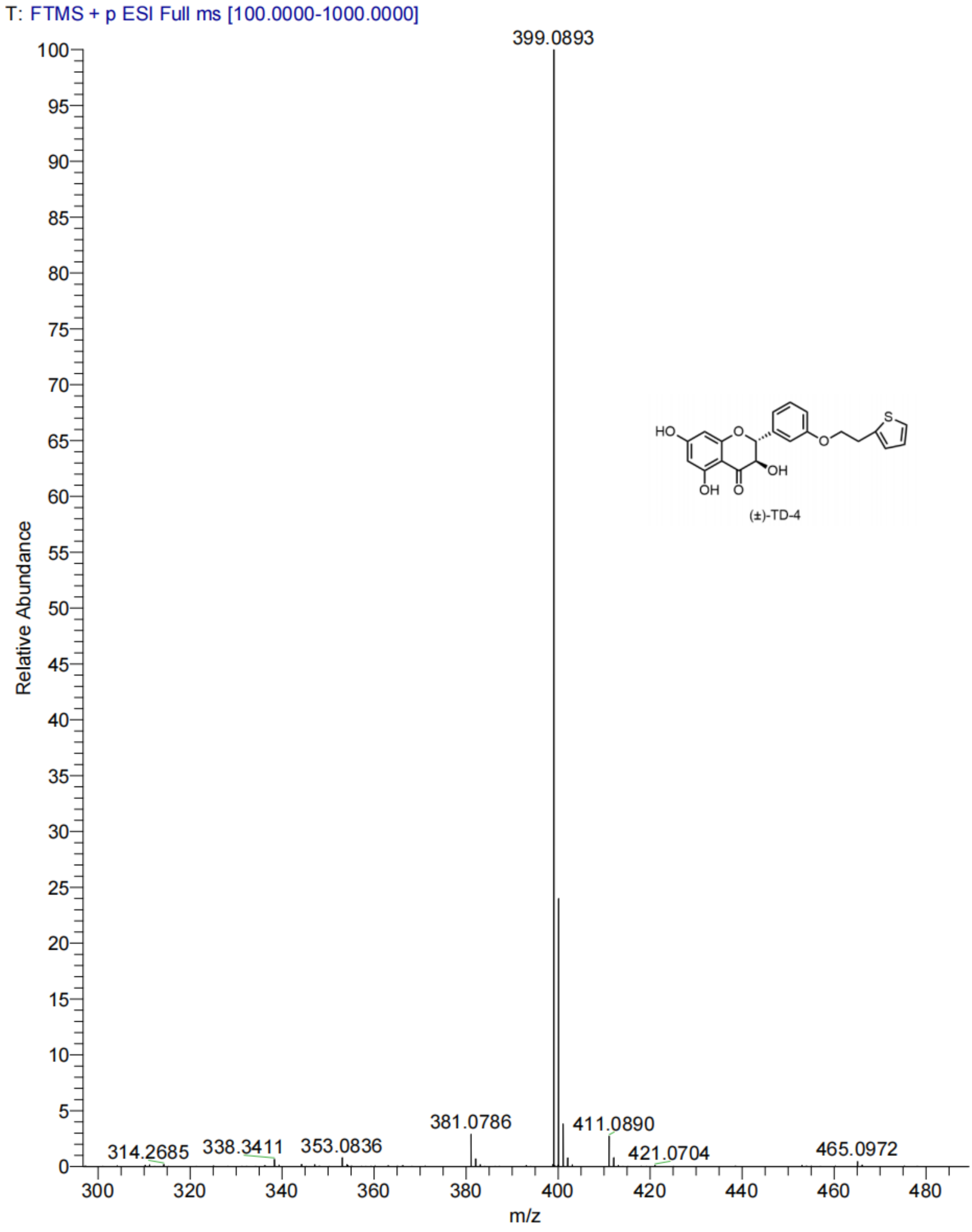

Supplement: File 1 — Reaction conditions screening, experimental section with characterization data and copies of spectra. [file Beilstein_J_Org_Chem-22-443-s001.pdf]
